# Four of a Kind: A Complete Collection of ADP-Ribosylated Histidine Isosteres Using Cu(I)- and Ru(II)-Catalyzed Click Chemistry

**DOI:** 10.1021/acs.joc.3c00827

**Published:** 2023-07-19

**Authors:** Hugo Minnee, Hayley Chung, Johannes Gregor Matthias Rack, Gijsbert A. van der Marel, Herman S. Overkleeft, Jeroen D. C. Codée, Ivan Ahel, Dmitri V. Filippov

**Affiliations:** † Bio-Organic Synthesis, Leiden Institute of Chemistry, Leiden University, Leiden 2300 RA, The Netherlands; ‡ Sir William Dunn School of Pathology, University of Oxford, South Parks Road, Oxford OX1 3RE, U.K.

## Abstract

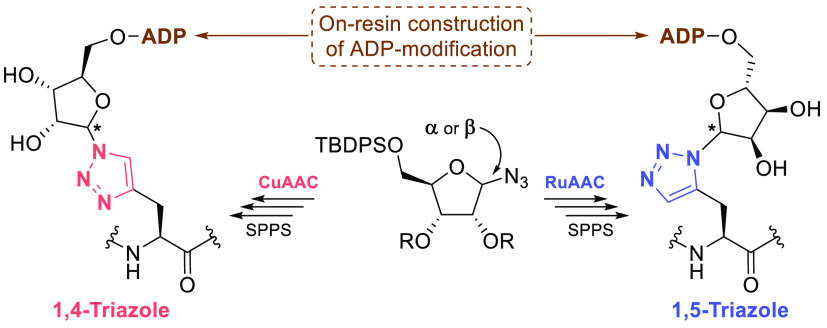

Adenosine diphosphate ribosylation (ADP-ribosylation) is a crucial post-translational modification involved in important regulatory mechanisms of numerous cellular pathways including histone maintenance and DNA damage repair. To study this modification, well-defined ADP-ribosylated peptides, proteins, and close analogues thereof have been invaluable tools. Recently, proteomics studies have revealed histidine residues to be ADP-ribosylated. We describe here the synthesis of a complete set of triazole-isosteres of ADP-ribosylated histidine to serve as probes for ADP-ribosylating biomachinery. By exploiting Cu(I)- and Ru(II)-catalyzed click chemistry between a propargylglycine building block and an α- or β-configured azidoribose, we have successfully assembled the α- and β-configured 1,4- and 1,5-triazoles, mimicking N(τ)- and N(π)-ADP-ribosylated histidine, respectively. The ribosylated building blocks could be incorporated into a peptide sequence using standard solid-phase peptide synthesis and transformed on resin into the ADP-ribosylated fragments to provide a total of four ADP-ribosyl triazole conjugates, which were evaluated for their chemical and enzymatic stability. The 1,5-triazole analogues mimicking the N(π)-substituted histidines proved susceptible to base-induced epimerization and the ADP-ribosyl α-1,5-triazole linkage could be cleaved by the (ADP-ribosyl)hydrolase ARH3.

## Introduction

Adenosine diphosphate ribosylation is a highly versatile and dynamic post-translational modification (PTM) in which the well-known redox co-factor nicotinamide dinucleotide adenine (NAD^+^) is used to covalently link an adenosine diphosphate ribose (ADPr) molecule to a nucleophilic amino acid functionality. It is a ubiquitously expressed modification that allows spatiotemporal regulation of important cellular pathways including adipogenesis,^1^ DNA damage repair, gene expression,^2^ and apoptosis.^3^ ADP ribosylation is affected by a family of (ADP-ribosyl)transferase enzymes termed PARPs. Most members transfer a single ADPr moiety to a nucleophilic acceptor, which is referred to as mono-ADP-ribosylation (MARylation), although a small subset of PARPs (PARP1, 2, 5a and 5b) are able to mediate poly-ADP-ribosylation (PARylation) to create a linear polymer^4^ with occasional branches.^5^ The resulting poly-ADPr chains can be truncated by poly(ADP-ribosyl) glycohydrolase (PARG)^6,7^ to yield a MARylated protein, after which a collection of (ADP-ribosyl)hydrolases and macrodomain proteins with distinct substrate specificity remove the final protein-linked ADPr moiety.^8−12^

The most common amino acid residue to be ADP-ribosylated is serine,^13,14^ but glutamate, aspartate,^1,15−17^ arginine,^18^ cysteine,^13,19^ lysine, and more recently tyrosine^13,20^ and histidine^21,22^ have been found to be ADP-ribosylated as well. Synthetic, well-defined MARylated peptides and ADPr-oligomers have been shown to be valuable molecular tools to investigate ADP-ribosylation, informing on the exact structure of ADPr polymers and modified peptides, the chemical and enzymatic stability of the PTMs, and the binding with interaction partners.^23−26^ Various isosteres of ADP-ribosylated amino acids have been introduced as ADPr chemical biology tools with special attention being paid to stabilizing the glycosidic linkage that connects the ADPr moiety to a protein and to expedite synthetic accessibility. Examples of the isosteric replacements for native ADPr-peptides include ADP-ribosylated glutamine and asparagine^27^ and *N*-methyl aminooxy functionalized peptides^28^ serving as base-stable substitutes for their glutamate and aspartate counterparts. Likewise, the urea functionality of citrulline has been introduced as a mimic for the guanidine group of arginine.^27^ Click chemistry has been implemented to generate non-natural MARylated oligopeptides^29−31^ and even full-length proteins, as was demonstrated by the synthesis of ADP-ribosylated ubiquitin, carrying the ADPr moiety at specific arginine residues.^32^

**Figure 1:**
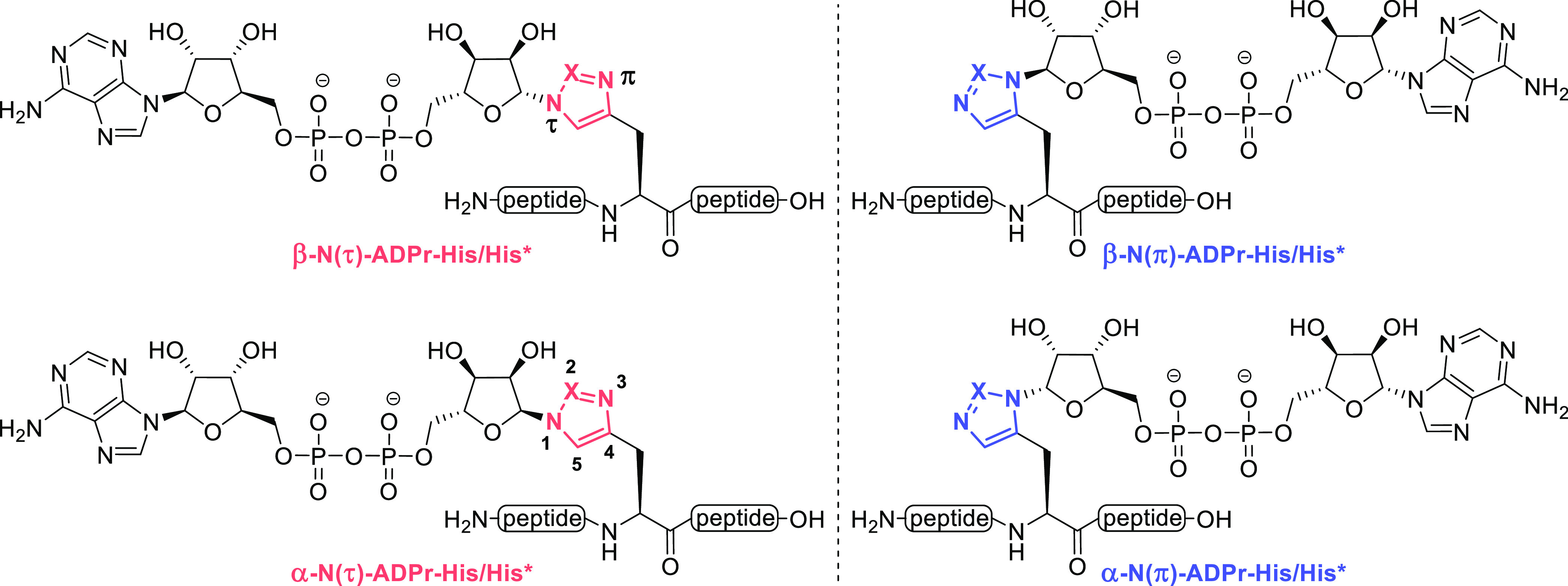
All four possible chemical structures for ADP-ribosylated histidine residues (X=CH), including the pros (“near,” π) and tele (“far,” τ) terminology for the imidazolium nitrogen atoms. The 1,4- and 1,5-disubstituted triazole-based isosteres (X=N, referred to as His*) mimic their N(τ)- and N(π)-ADP-ribosylated histidine counterparts, respectively. The numbering nomenclature of triazolyl-functionalities is highlighted.

The imidazole ring of histidine has recently been identified as a potential ADP-ribosylation site.^21^ Proteomics studies have however been unable to elucidate the nature of the ribosyl–histidine linkage. The imidazolyl side chain of histidine carries two possible modification sites that are commonly referred to as the N(π)- and N(τ)-positions (Figure 1). In addition, the chirality of the ribosyl anomeric center is unknown. Although all ADP-ribosyl linkages identified to date are α-configured (as a result of the substitution of the NAD^+^ nicotinamide with inversion of stereochemistry), it cannot a priori be ruled that the linkage to the histidine imidazolyl group cannot be β-configured. For example, ADPr-Arg has been shown to spontaneously anomerize under physiological conditions via an endocyclic ring-opening pathway to transform the α-ribosyl linkage into the corresponding β-ribose.^33^ We have previously reported on the synthesis of triazolyl-linked N(τ)-ADP-ribosylated conjugates that function as histidine isosteres, indicated here as ADPr-His*. We generated peptides with ADPr-His* via a convergent synthesis, introducing the 1,4-triazole moieties by exploiting the highly regioselective Cu(I)-catalyzed azide–alkyne cycloaddition (CuAAC) of azido-ADP-ribose to a propargylglycine residue, pre-installed in the peptide chain by solid-phase peptide synthesis.^31^ We also showed that the glycosidic linkage of these isosteres could be cleaved by ARH3, an (ADP-ribosyl)hydrolase, capable of clipping off ADPr moieties from ADPr-Ser,^23^*O-*acetyl-ADPr,^34^ α-NAD^+^,^35^ poly-ADPr,^36^ as well as ADPr-5′-P DNA.^37^ The 1,4-triazole ADPr-His* was however a relatively weak substrate for ARH3.^31^

To expand the set of probes to investigate ADPr-histidine biology, we report here on the assembly of the full set of four possible ADP-ribosyl triazole histidine mimics and describe a synthetic methodology to access both 1,4-triazoles, resembling N(τ)-ADPr-histidine and 1,5-triazoles mimicking the N(π)-ADPr- histidine, having either the α- or β-ribosyl configuration (Figure 1). We have incorporated the (α/β)-1,4/1,5-triazole ADPr-His* mimics in a peptide fragment originating from histone PARylation factor 1 (HPF1), as the histidine residue in this peptide has been identified as a ribosylation site in recent proteomics studies.^21^ The peptides have been used for stability studies to probe the integrity of the fragments under conditions typically used in proteomics workflows, and we have subjected the ADPr-His* peptides to a panel of (ADP ribosyl)hydrolases. These studies have revealed the 1,5-triazole analogues to be less stable under basic conditions and to be better substrates for ARH3 than their 1,4-counterparts.

## Results and Discussion

**Figure 2:**
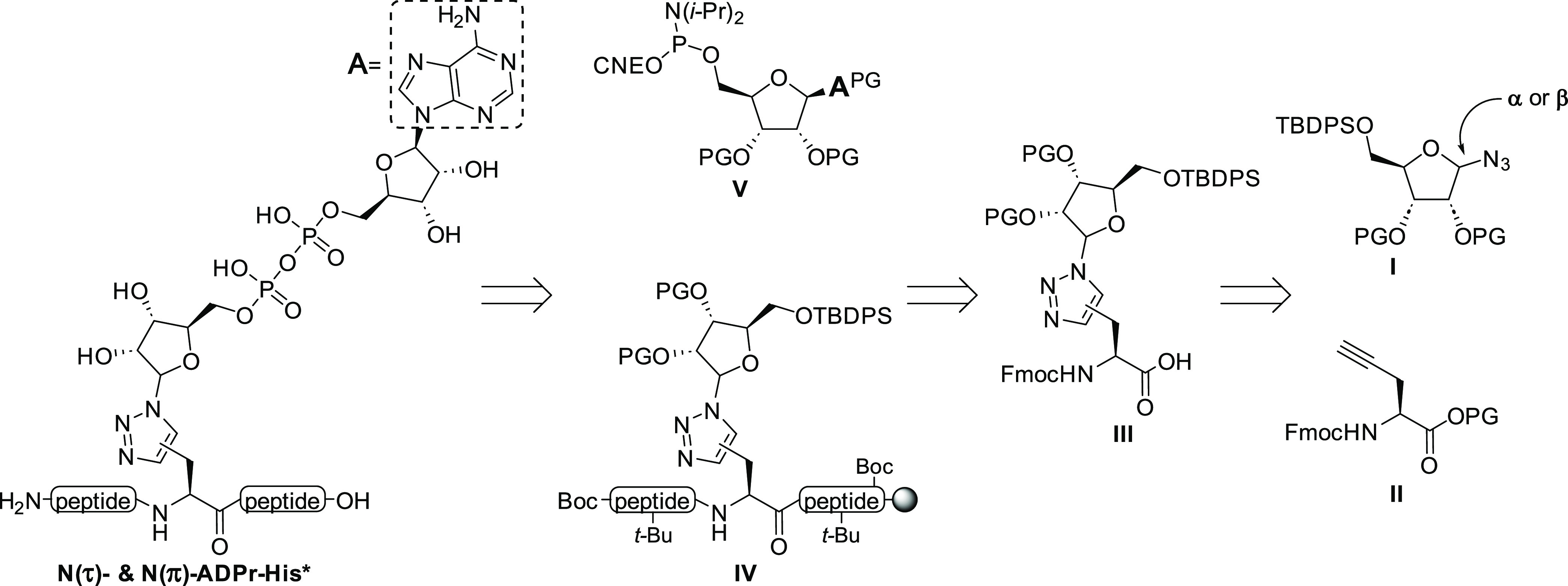
Retrosynthetic analysis of 1,4- and 1,5-disubstituted triazole-based isosteres for N(τ)- and N(π)-ADP-ribosylated histidine, respectively, referred to as N(τ)- and N(π)-ADPr-His*. HPF1_221–233_ (T-F-H*-G-A-G-L-V-V-P-V-D-K, where H* refers to the triazole isostere) includes the histidine modification site that was recently identified in proteomic experiments.^21^ Here, adenine is abbreviated as **A** and PG is used to depict an unspecified protecting group.

We previously generated the α/β-(1,4)-triazole His-mimetics through a regioselective CuAAC-reaction, and we reasoned that we could exploit the less common Ru(II)-catalyzed azide–alkyne cycloaddition (RuAAC)^38^ to access their (α/β)-1,5-triazole counterparts. While the RuAAC has been successfully applied to peptides before,^39^ we found the conditions to be incompatible with the late-stage conjugation of an azido-ADP-ribose to an oligopeptide carrying an alkyne click handle and only observed degradation of the azido-ADP-ribose. Therefore, an alternative approach toward the 1,5-disubstituted triazole-isosteres for ADPr-histidine was required and we opted for a stepwise SPPS approach where a suitably protected ribosyl azide **I** was first conjugated to fluorenylmethyloxycarbonyl (Fmoc)-propargylglycine **II** (Figure 2). The resulting Fmoc-building block **III** could then be used in a SPPS protocol for the incorporation in a peptide sequence of choice **IV** through standard Fmoc-based peptide chemistry. Next, on resin phosphorylation and pyrophosphate formation following a well-established P(III)-P(V) coupling strategy^40^ with suitably protected adenosine amidite **V** and deprotection should enable the synthesis of the ADPr-His* mimetics. Using Cu(I)- or Ru(II)-catalyzed click chemistry in combination with either α- and β-azidoribose grants access to the full set of (α/β)-N(τ)/N(π)-ADPr-His* isosteres.

### β-Ribosyltriazolylalanine Building Blocks **9** and **11**

**Figure 3:**
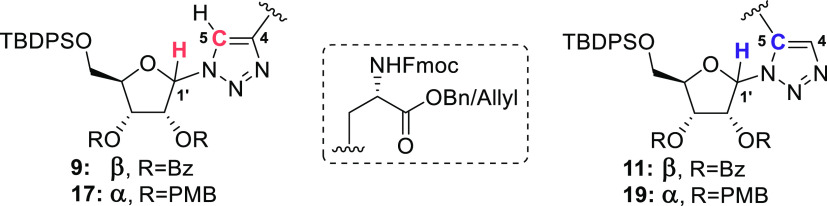
Characteristic proton–carbon three-bond couplings that have been observed in HMBC measurements for the here-described 1,4- and 1,5-disubstituted triazoles building blocks are highlighted in red and blue, respectively. No correlation between H-5 and C-1′ was observed for any of the 1,4-triazoles in the HMBC data sets.

**Scheme 1:**
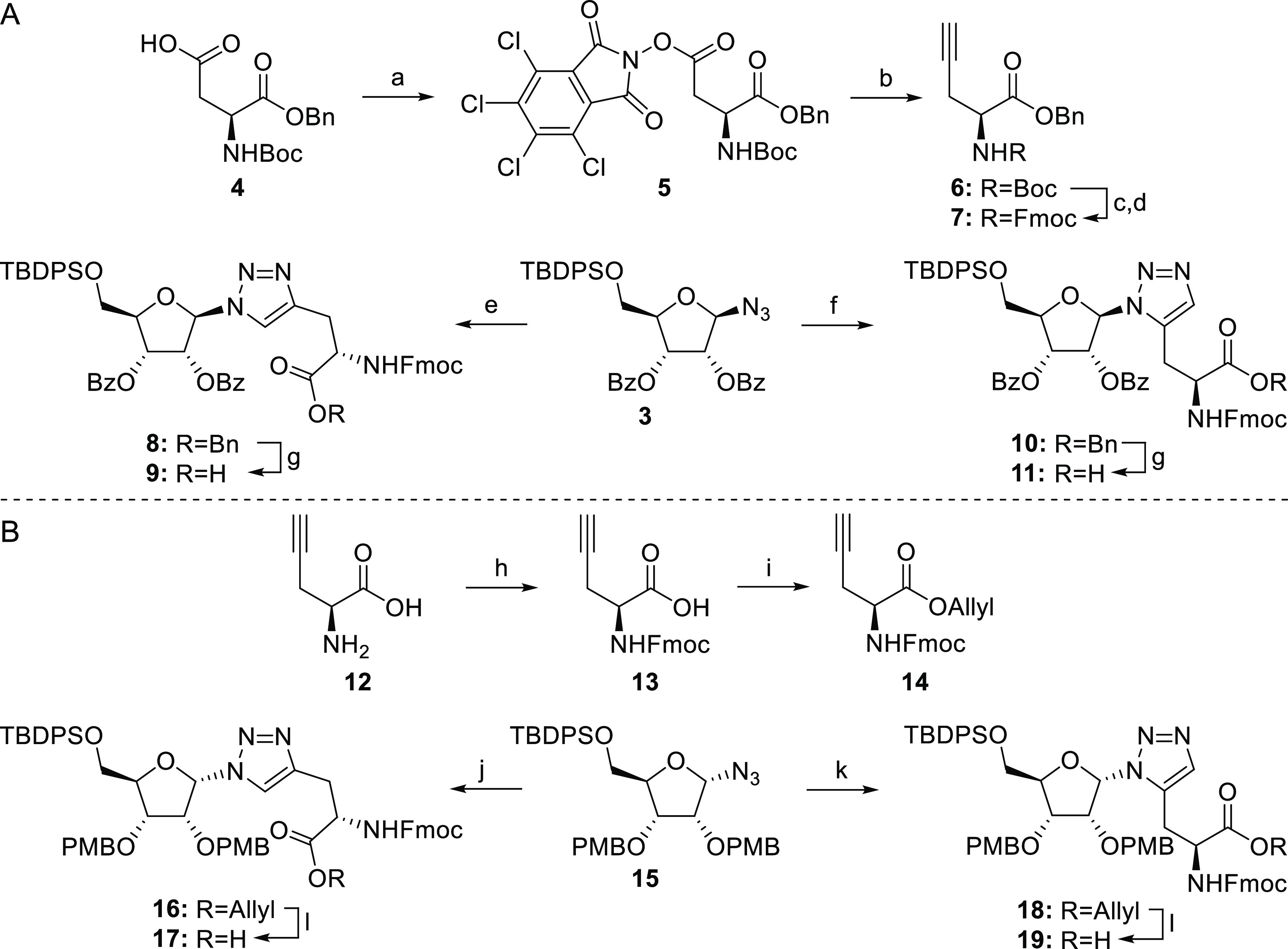
Preparation of Triazolyl-Linked Ribosylated Building Blocks through Cu(I)- and Ru(II)-Catalyzed Cycloadditions between Suitably Protected Propargylglycine Analogues with β-Configured Azido-Ribofuranoside **3** (A) or α-Configured Azido-Ribofuranoside **15** (B) Reagents and conditions: (a) *N*-hydroxytetrachlorophthalimide, DCC, DMAP, DCM, rt, 16 h (81%). (b) Ethynylmagnesium bromide, 4,4-dimethoxy-2,2′-bipyridine, NiCl_2_, ZnCl_2_, LiCl, THF/DMF (1:1), rt, 16 h (73%). (c) TFA, DCM, rt, 2 h. (d) Fmoc-OSu, NaHCO_3_, H_2_O/MeCN (1:1), rt, 16 h (91% over 2 steps). (e) **7**, CuSO_4_, sodium ascorbate, DMF, rt, 1 h (71%). (f) **7**, Cp*ClRu(COD), THF, microwave, 100 °C, 5 min (85%). (g) H_2_ (1 atm), Pd/C, MeOH, rt, 3.5 h (80% for **9**, 90% for **11**). (h) Fmoc-OSu, NaHCO_3_, H_2_O/THF (1:1), rt, 16 h (quant). (i) AllylOH, DMAP, DIC, DCM, rt, 45 min (81%). (j) **14**, CuSO_4_, sodium ascorbate, DMF, rt, 1 h (93%). (k) **14**, Cp*ClRu(COD), THF, microwave, 100 °C, 1 h (60%). (l) Pd(PPh_3_)_4_, DMBA, DCM, rt, 1.5 h (96% for **17**, 85% for **19**).

The preparation of 1,4- and 1,5-disubstituted triazole building blocks that are compatible with Fmoc-based SPPS commenced with the synthesis of β-configured azide **3**, which was derived from the commercially available ribose tetraacetate over four steps according to previously reported literature procedures (Scheme S1).^31,41^ The required propargylglycine could be accessed from commercially available Boc–Asp–OBn **4** through a radical-mediated^42,43^ decarboxylative alkynylation, as described by Baran et al. for the synthesis of homo-propargylglycine from Boc–Glu–OBn.^44^ To this end, Boc–Asp–OBn **6** was transformed in redox-active ester **5** via a Steglich esterfication (Scheme 1A). Ethynylzinc chloride was prepared *in situ* from its Grignard precursor and an equimolar mixture of zinc chloride and lithium chloride in THF. To ensure the efficient and consistent formation of alkyne **6**, it was found that the bipyridine–Ni(II) complex solution and a large excess of the ethynylzinc chloride had to be added to phthalimide **5** in quick succession. This way the fully protected propargylglycine **6** was obtained in 73% yield. Protecting group manipulations then provided Fmoc-propargylglycine benzyl ester **7**, which could be conjugated to β-configured azide **3** in a Cu(I)- or Ru(II)-catalyzed cycloaddition. Successive addition of CuSO_4_ and sodium ascorbate to a solution containing equimolar amounts of azide **3** and propargylglycine **7** in DMF provided a single product (**8**) that was conveniently isolated by silica gel column chromatography. Formation of 1,4-disubstituted triazole **8** was confirmed using heteronuclear multiple bond correlation (HMBC) NMR spectroscopy, which revealed a clear coupling between the ribosyl H-1′ and the tertiary C-5 of the triazole unit (Figure 3).

To generate the alternative 1,5-triazole, azide **3** was clicked to alkyne **7** using the chloro(pentamethylcyclo-pentadienyl)(cyclo octadiene)ruthenium (II) (Cp*RuCl(COD)) catalyst. An almost immediate conversion of the click partners **3** and **7** was realized by microwave heating the components in THF at 100 °C in the presence of the Cp*RuCl(COD) catalyst to provide 1,5-triazole **10** in 85% yield.^45^ The isolated product clearly differed from the previously isolated 1,4-regioisomer **8** according to both ^1^H and ^13^C NMR. HMBC measurements revealed a strong coupling between the ribosyl H-1′ and the quaternary C-5 of the triazole moiety proving the regioselective formation of the 1,5-disubstituted triazole **10** (Figure 3). Deprotection of the benzyl esters in **8** and **10** provided the β-configured building blocks **9** and **11** for the planned Fmoc-based SPPS endeavors.

### α-Ribosyltriazolylalanine Building Blocks **17** and **19**

In the syntheses of the corresponding 1,4- and 1,5-triazole α-ribosyl building blocks, non-participating benzyl-type protecting groups are required to install the α-azido ribosyl linkages. Therefore, the benzyl ester **7** described above cannot be used to generate the triazole amino acid and we switched to the use of an allyl ester to mask the amino acid carboxylate. Since glutamic acid allyl esters are not readily commercially available, the route described above for the benzyl ester could not be followed and we generated the required building block **14** from commercially available propargylglycine **12** (Scheme 1B). Introduction of the Fmoc group under basic conditions in a H_2_O/THF solvent system gave carboxylic acid **13**, which was converted into **14** via a Steglich esterification with DIC/DMAP and allyl alcohol. α-Configured azide **15** was prepared as described previously,^31^ and both click partners could be joined by applying the same reaction conditions as discussed above to give 1,4-triazole **16** in good yield. The RuAAC-reaction to generate the 1,5-regioisomer **18** however required some optimization. Incomplete conversion of azide **15** was observed when a small excess of alkyne **14** (1.3 equiv) was used, and even when an extended reaction time (>1 h) was used, the yield did not exceed 35%. Using a 2-fold excess of alkyne **14** eventually led to the formation of 1,5-triazole **18** in a satisfactory yield of 60%. The regiochemistry in **16** and **18** was again substantiated by HMBC data. Removal of the allyl ester functionality in **16** and **18** was mediated using a catalytic amount of tetrakis(triphenylphosphine)palladium(0) in the presence of 1,3-dimethylbarbituric acid (DMBA) as an allyl scavenger to provide the desired α-configured SPPS building blocks **17** and **19**. With both anomeric configurations of ribosylated 1,4- and 1,5-triazoles in hand, we next set out to generate the set of target ADPr-His* peptides using SPPS.

### Solid-Phase Synthesis of ADPr-His* Peptides

**Scheme 2:**
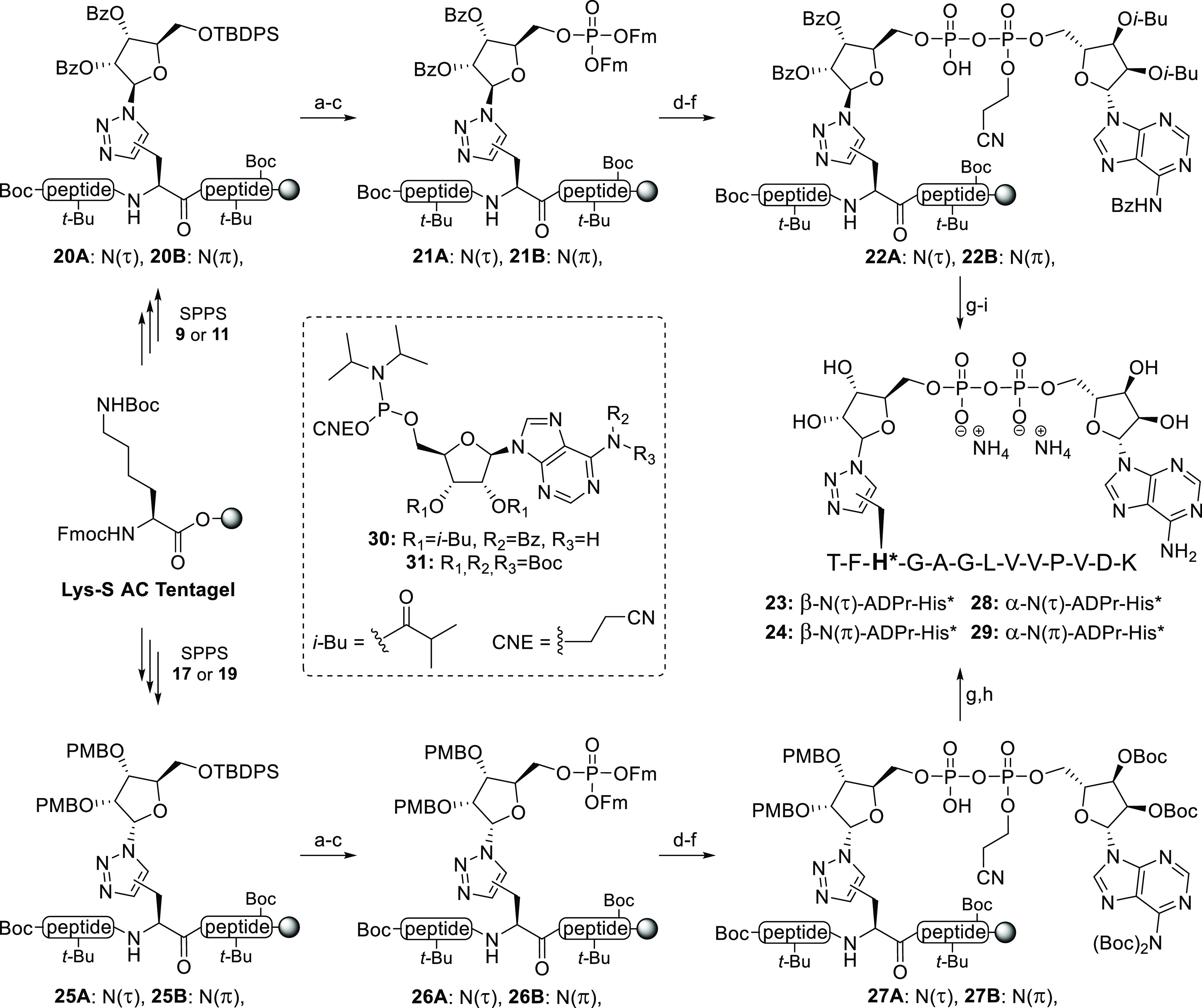
Incorporation of Ribosylated Amino Acids **9, 11, 17**, and **19** in a Peptide Fragment Originating from HPF1, Where H* Refers to the Triazole Isostere, Resulting in (τ)- and (π)-His* Isosteres in Both α- and β-Configuration Reagents and conditions: (a) HF-pyridine, pyridine, rt, 2 × 45 min. (b) (FmO)_2_PN(*i-*Pr)_2_, ETT, MeCN, rt, 30 min. (c) CSO, MeCN, rt, 30 min. (d) DBU, DMF, rt, 2 × 15 min. (e) **30** or **31**, ETT, MeCN, rt, 30 min. (f) CSO, MeCN, rt, 30 min. (g) DBU, DMF, rt, 2 × 10 min. (h) TFA, DCM, rt, 1 h (36 and 25% over 8 steps for **28** and **29**, respectively). (i) NH_4_OH (28%), rt, 16 h (26 and 9% over 9 steps for **23** and **24**, respectively).

As described above, peptides from histone PARylation factor 1 (HPF1) have been identified as the ADP-ribosylation site in recent proteomic studies,^21^ and therefore, we incorporated the α/β-N(τ)/N(π)-ADPr-His* analogues in the corresponding peptide fragment derived from this protein.^31^ Starting from Tentagel S AC resin, preloaded with *tert*-butyloxycarbonyl (Boc)-protected lysine, β-ribosyltriazolides **9** and **11** were incorporated using standard Fmoc-based SPPS conditions to provide fully protected intermediates **20A/B** (Scheme 2). Next, we planned to install the pyrophosphate moiety by unmasking the primary alcohol, installing the primary phosphate, and extending this using a P(V)-P(III) coupling with a suitably protected adenosine phosphoramidite. Initially, we explored the removal of the silyl protecting group with tetrabutylammonium fluoride (TBAF) in THF,^46^ but LC-MS analysis indicated that desilylation was accompanied by loss of the 2′- and/or 3′-benzoyl moieties. This also implied that the C-terminal ester linkage to the resin might be at risk. Fortunately, HF-pyridine proved to be an adequate and the milder alternative, leaving the ester functionalities unscathed while efficiently removing the TBDPS group. The liberated alcohol was conjugated to a 9-fluorenylmethyl (Fm)-protected phosphoramidite^47^ in the presence of 5-(ethylthio)-tetrazole (ETT) as an activator. The resulting phosphotriester was subsequently oxidized with (1S)-(+)-(10-camphorsulfonyl)-oxaziridine (CSO) to provide protected phosphates **21A/B**. 1,8-Diazabicyclo[5.4.0]undec-7-ene (DBU) allowed for effective removal of the Fm-moieties, after which construction of the pyrophosphate was realized with adenosine amidite **30**(48) using P(III)-P(V) coupling chemistry under the *aegis* of ETT.^40^ CSO-mediated oxidation of the phosphate-phosphite intermediate into the pyrophosphate provided fully protected ADPr-conjugates **22A/B**. After deprotection of the 2-cyanoethyl group with DBU, the oligopeptide was treated with 50% trifluoroacetic acid (TFA) to ensure cleavage of the construct from the Tentagel resin while simultaneously removing the *t*-Bu and Boc protecting groups. Global deprotection of the remaining base sensitive groups was affected by the treatment of the crude material with aqueous ammonia overnight, and this was followed by purification using preparative HPLC (NH_4_OAc buffered) to yield the β-configured N(τ)- and N(π)-ADPr-His* peptides **23** and **24** in 36 and 25% yield, respectively.

In a similar manner as described above, the α-configured building blocks **17** and **19** were incorporated in the peptide backbone, after which the resin-bound intermediates were desilylated with HF-pyridine and phosphorylated in a two-step fashion to give the Fm-phosphates **26A/B**. After treatment with DBU, the liberated phosphate was coupled to fully Boc-protected adenosine amidite **31** to yield ADPr-peptides **27A/B**.^49^ After the elimination of the 2-cyanoethyl functionality, all remaining protecting groups were successfully removed during the final cleavage with TFA. Identical preparative HPLC conditions allowed for the isolation of the two remaining α-configured N(τ)- and N(π)-histidine isosteres **28** and **29**.

**Figure 4:**
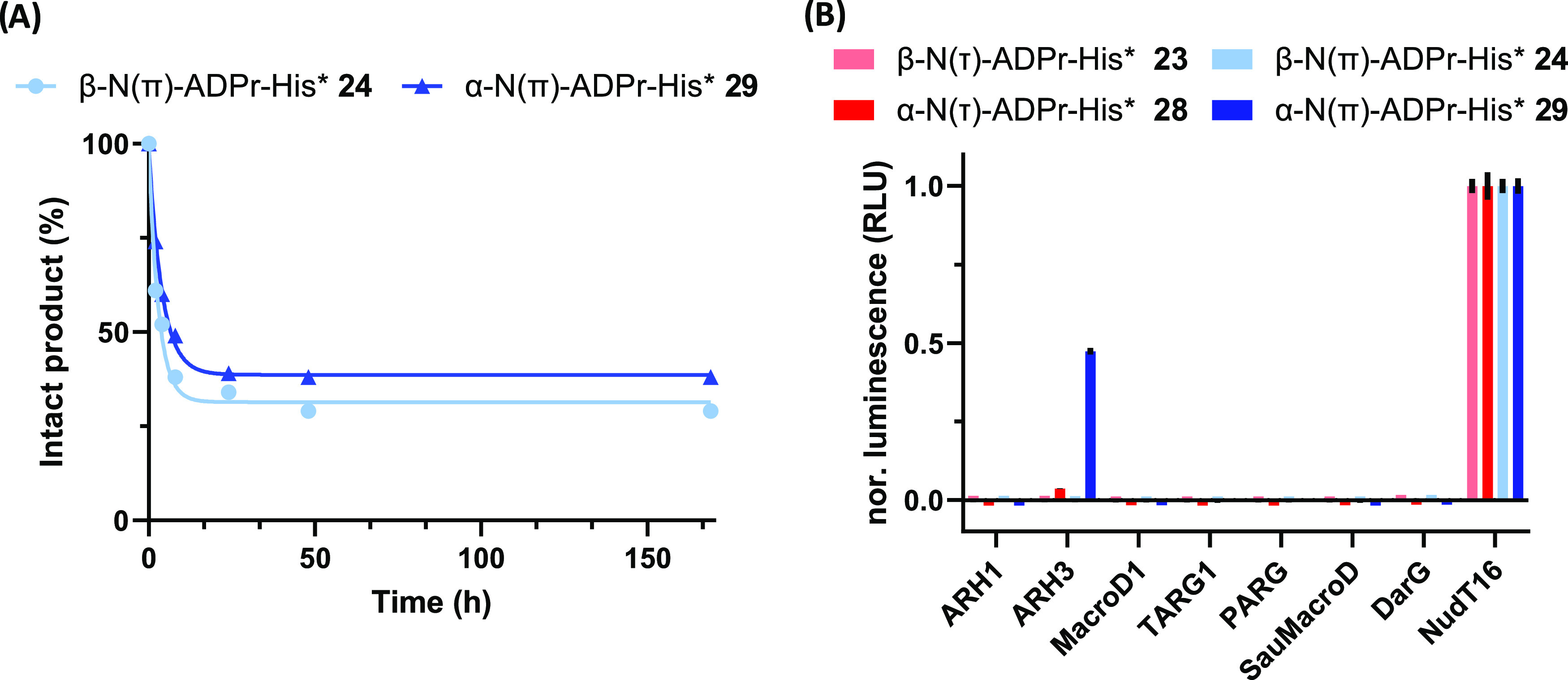
(A) Chemical stability of 1,5-disubstitued triazoles **24** and **29** under basic conditions (NaOH, 0.1 M). Samples were extracted at different time points (2, 4, 8, 24, 48, and 169 h) and quenched with TFA prior to LC-MS injection. Peptide degradation was quantified by analyzing the UV-trace (260 nm) using Xcalibur software. Including an exponential one-phase decay trendline (*R*^2^ values are 0.986 and 0.997 for **24** and **29**, respectively). (B) Enzymatic hydrolysis of the ribosyl linkages in ADP-ribosylated histidine peptides **23, 24, 28**, and **29**. Enzymatic turnover of the various peptides was assessed by monitoring AMP release directly (NudT16) or converting released ADPr via NudT5 to AMP. AMP was measured using the AMP-Glo assay (Promega). Samples are background corrected and normalized to NudT16 activity.

With all four ADPr-His* conjugates available, we next evaluated their stability under various conditions in a liquid-chromatography mass-spectrometry (LC-MS) based assay. The conditions surveyed (NH_2_OH, TFA, and NaOH) were selected because of their frequent occurrence in ADP-ribosylome-focused proteomics studies. In these studies, NH_2_OH elimination steps are implemented to identify acidic ADP-ribosylated residues^1,16^ while basic conditions are applied for the pre-fractionation of peptides, which is generally followed by a subsequent acid treatment.^13,22^ All four isosteres remained unaffected under the TFA (0.1 M) or NH_2_OH (0.5 M) conditions for at least 24 h, as no sign of degradation was observed by LC-MS. After 24 h of 0.1 M NaOH treatment, LC-MS analysis showed additional peaks for the β-N(τ)-ADPr-His* **23** and α-N(τ)-ADPr-His* **28** peptides. These peaks (5 and 12%) corresponded to products having an identical mass as the parent compounds, indicating an isomerization reaction. As the newly formed peaks did not correspond to the anomeric counterparts of the starting compounds, we assume these products to originate from epimerization of the His* α-carbon, as histidine has been observed to be relatively prone to epimerization. The steric hindrance in the more crowded 1,5-triazoles possibly makes these peptides more susceptible to the base-mediated epimerization than their 1,4-counterparts. We monitored the isomerization of the 1,5-triazoles β-N(π)-ADPr-His* **24** and α-N(π)-ADPr-His* **29** in a follow-up time course experiment (Figure 4A) and observed rapid consumption of the starting peptides leading to a mixture of products in which 38% and 30% of the original peptides **24** and **29**, respectively, were present after 12 h.

Finally, we assessed the susceptibility of the peptides toward (ADP-ribosyl)hydrolase-mediated hydrolysis (Figure 4B). Each of triazole conjugates **23, 24, 28**, and **29** was incubated with a set of purified human (ADP-ribosyl)hydrolases. Any ADPr freed by the hydrolase in these reactions was converted by the NudT5 enzyme into adenosine monophosphate (AMP and quantified using the AMP-Glo assay (Promega)).^50^ As a positive control, the samples were treated with NudT16, which is able to hydrolyze the pyrophosphate linkage of both free and peptide-bound ADPr.^51^ None of the hydrolases were able to cleave the *N*-glycosidic linkage of any of the four isosteres, except for ARH3, which was capable of hydrolyzing α-N(π)-ADPr-His* **29** and very weakly α-N(τ)-ADPr-His* **28**. Both β-anomers **23** and **24** remained unaffected by ARH3 under the given conditions. The slow but enzyme-dependent cleavage of α-N(τ)-ADPr-His* **28** corresponds to the hydrolysis we described in our previous study.^31^ Of note, the hydrolysis rate for α-N(π)-ADPr-His* **29** was substantially higher than its N(τ)-regioisomer **28**, leading to ∼50% conversion within the 1 h reaction. This may indicate that N(π)-ADPr-His is the naturally occurring isomer and that ARH3 could be the hydrolase involved in trimming ADP-ribosyl units from ADP-ribosylated histidine-containing proteins. It should be pointed out, however, that ARH3 is a rather promiscuous hydrolase^23,35^ and the susceptibility to ARH3-mediated cleavage of the 1,5-triazoles can also be due to enhanced lability of these substrates as inferred from the stability studies described above.

## Conclusions

A comprehensive SPPS-based methodology toward both 1,4- and 1,5-disubstituted triazole ADPr-peptide conjugates has been developed to mimic ADP-ribosyl histidine peptides. To this end, the regioselectivity of Cu(I)- and Ru(II)-catalyzed click reactions has been exploited to join a propargylglycine and an α- or β-configured azido ribosyl building block to furnish a complete set of (α/β)-N(τ)/N(π)-ADPr-His mimetics. Incorporation of these novel building blocks in a peptide sequence of interest was accomplished uneventfully using standard Fmoc-based SPPS conditions. Desilylation of the resulting resin-bound intermediates was performed with a slightly acidic HF-pyridine instead of TBAF, which we previously employed,^46^ to minimize the degradation of the ester linkages. The adenosine diphosphate moiety could be readily introduced through phosphoramidite chemistry to effectively deliver, after a sequence of deprotection steps, the target triazolyl peptide conjugates **23**, **24**, **28**, and **29** in good to satisfactory yields. The stability of the peptides under nucleophilic (0.5 M NH_2_OH), acidic (0.1 M TFA), and basic (0.1 M NaOH) conditions, commonly employed in ADP-ribosyl proteomics protocols, was assessed, revealing the 1,5-triazoles to be sensitive to base-assisted epimerization. A NudT-based luminescent assay enabled the quantification of (ADP-ribosyl)hydrolase-mediated degradation of the *N*-glycosidic linkage in the triazole constructs. Both α-N(τ)-ADPr-His* **28 and** α-N(π)-ADPr-His* **29** proved to be susceptible to ARH3-mediated hydrolysis, with the 1,5-triazole **29** being significantly more labile than its 1,4-triazole counterpart **28**. These findings may suggest the α-N(π)-His to be the naturally occurring isomer, although the chemical stability studies also point to the intrinsic lability of the 1,5-triazole conjugates. Our findings warrant the development of a synthetic methodology to access ADP-ribosylated histidines, featuring the natural linkages, to determine whether the observed hydrolytic activity of ARH3 is of real biological relevance and applies to the natural imidazolyl–glycosidic linkages. It is expected that the methodology described here can be transposed to many other peptide sequences to effectively deliver ADPr-His* tools for (structural) biology purposes.

## Data Availability

The data underlying this study are available in the published article and its online Supporting Material.

## Supporting Information Available

Procedures for the expression of plasmids and the following protein purification; procedure for the (ADP-ribosyl)hydrolase activity assay; chemical procedures including the relevant ^1^H, ^13^C, ^31^P, HMBC NMR spectra for new compounds; spectra of the LC-MS-based stability assay performed for peptides **23**, **24**, **28**, and **29** (PDF)

## References

[ref1] Huang D.; Camacho C. V.; Setlem R.; Ryu K. W.; Parameswaran B.; Gupta R. K.; Kraus W. L. Functional Interplay between Histone H2B ADP-Ribosylation and Phosphorylation Controls Adipogenesis. Mol. Cell 2020, 79, 934–949. 10.1016/j.molcel.2020.08.002. 32822587 PMC7502539

[ref2] Dantzer F.; Santoro R. The Expanding Role of PARPs in the Establishment and Maintenance of Heterochromatin. FEBS J. 2013, 280, 3508–3518. 10.1111/febs.12368. 23731385

[ref3] Mashimo M.; Kato J.; Moss J. ADP-Ribosyl-Acceptor Hydrolase 3 Regulates Poly (ADP-Ribose) Degradation and Cell Death during Oxidative Stress. Proc. Natl. Acad. Sci. U.S.A. 2013, 110, 18964–18969. 10.1073/pnas.1312783110. 24191052 PMC3839768

[ref4] Miwa M.; Saitô H.; Sakura H.; Saikawa N.; Watanabe F.; Matsushima T.; Sugimura T. A 13C NMR Study of Poly(Adenosine Diphosphate Ribose) and Its Monomers: Evidence of Alpha-(1″ Leads to 2′) Ribofuranosy1 Ribofuranoside Risidue. Nucleic Acids Res. 1977, 4, 3997–4005. 10.1093/nar/4.11.3997. 593897 PMC343216

[ref5] Miwa M.; Ishihara M.; Takishima S.; Takasuka N.; Maeda M.; Yamaizumi Z.; Sugimura T.; Yokoyama S.; Miyazawa T. The Branching and Linear Portions of Poly(Adenosine Diphosphate Ribose) Have the Same Alpha(1 Leads to 2) Ribose-Ribose Linkage. J. Biol. Chem. 1981, 256, 2916–2921. 10.1016/S0021-9258(19)69701-2. 6782097

[ref6] Lin W.; Amé J.-C.; Aboul-Ela N.; Jacobson E. L.; Jacobson M. K. Isolation and Characterization of the CDNA Encoding Bovine Poly(ADP-Ribose) Glycohydrolase. J. Biol. Chem. 1997, 272, 11895–11901. 10.1074/jbc.272.18.11895. 9115250

[ref7] Slade D.; Dunstan M. S.; Barkauskaite E.; Weston R.; Lafite P.; Dixon N.; Ahel M.; Leys D.; Ahel I. The Structure and Catalytic Mechanism of a Poly(ADP-Ribose) Glycohydrolase. Nature 2011, 477, 616–620. 10.1038/nature10404. 21892188 PMC3184140

[ref8] Jankevicius G.; Hassler M.; Golia B.; Rybin V.; Zacharias M.; Timinszky G.; Ladurner A. G. A Family of Macrodomain Proteins Reverses Cellular Mono-ADP-Ribosylation. Nat. Struct. Mol. Biol. 2013, 20, 508–514. 10.1038/nsmb.2523. 23474712 PMC7097781

[ref9] Rosenthal F.; Feijs K. L. H.; Frugier E.; Bonalli M.; Forst A. H.; Imhof R.; Winkler H. C.; Fischer D.; Caflisch A.; Hassa P. O.; Lüscher B.; Hottiger M. O. Macrodomain-Containing Proteins Are New Mono-ADP-Ribosylhydrolases. Nat. Struct. Mol. Biol. 2013, 20, 502–507. 10.1038/nsmb.2521. 23474714

[ref10] Moss J.; Tsai S. C.; Adamik R.; Chen H. C.; Stanley S. J. Purification and Characterization of ADP-Ribosylarginine Hydrolase from Turkey Erythrocytes. Biochemistry 1988, 27, 5819–5823. 10.1021/bi00415a063. 3179279

[ref11] Fontana P.; Bonfiglio J. J.; Palazzo L.; Bartlett E.; Matic I.; Ahel I. Serine ADP-Ribosylation Reversal by the Hydrolase ARH3. eLife 2017, 6, e28533 10.7554/eLife.28533. 28650317 PMC5552275

[ref12] Sharifi R.; Morra R.; Denise Appel C.; Tallis M.; Chioza B.; Jankevicius G.; Simpson M. A.; Matic I.; Ozkan E.; Golia B.; Schellenberg M. J.; Weston R.; Williams J. G.; Rossi M. N.; Galehdari H.; Krahn J.; Wan A.; Trembath R. C.; Crosby A. H.; Ahel D.; Hay R.; Ladurner A. G.; Timinszky G.; Williams R. S.; Ahel I. Deficiency of Terminal ADP-Ribose Protein Glycohydrolase TARG1/C6orf130 in Neurodegenerative Disease. EMBO J. 2013, 32, 1225–1237. 10.1038/emboj.2013.51. 23481255 PMC3642678

[ref13] Buch-Larsen S. C.; Hendriks I. A.; Lodge J. M.; Rykær M.; Furtwängler B.; Shishkova E.; Westphall M. S.; Coon J. J.; Nielsen M. L. Mapping Physiological ADP-Ribosylation Using Activated Ion Electron Transfer Dissociation. Cell Rep. 2020, 32, 108176 10.1016/j.celrep.2020.108176. 32966781 PMC7508052

[ref14] Suskiewicz M. J.; Zobel F.; Ogden T. E. H.; Fontana P.; Ariza A.; Yang J.-C.; Zhu K.; Bracken L.; Hawthorne; Ahel D.; Neuhaus D.; Ivan A. HPF1 Completes the PARP Active Site for DNA Damage-Induced ADP-Ribosylation. Nature 2020, 579, 598–602. 10.1038/s41586-020-2013-6. 32028527 PMC7104379

[ref15] Burzio L. O.; Riquelme P. T.; Koide S. S. ADP Ribosylation of Rat Liver Nucleosomal Core Histones. J. Biol. Chem. 1979, 254, 3029–3037. 10.1016/S0021-9258(17)30178-3. 218926

[ref16] Zhang Y.; Wang J.; Ding M.; Yu Y. Site-Specific Characterization of the Asp- and Glu-ADP-Ribosylated Proteome. Nat. Methods 2013, 10, 981–984. 10.1038/nmeth.2603. 23955771

[ref17] Gagné J.-P.; Ethier C.; Defoy D.; Bourassa S.; Langelier M.-F.; Riccio A. A.; Pascal J. M.; Moon K.-M.; Foster L. J.; Ning Z.; Figeys D.; Droit A.; Poirier G. G. Quantitative Site-Specific ADP-Ribosylation Profiling of DNA-Dependent PARPs. DNA Repair 2015, 30, 68–79. 10.1016/j.dnarep.2015.02.004. 25800440

[ref18] Pedrioli D. M. L.; Leutert M.; Bilan V.; Nowak K.; Gunasekera K.; Ferrari E.; Ralph I.; Malmström L.; Hottinger M. O. Comprehensive ADP-Ribosylome Analysis Identifies Tyrosine as an ADP-Ribose Acceptor Site. EMBO Rep. 2018, 19, e45310 10.15252/embr.201745310. 29954836 PMC6073207

[ref19] Vyas S.; Matic I.; Uchima L.; Rood J.; Zaja R.; Hay R. T.; Ahel I.; Chang P. Family-Wide Analysis of Poly(ADP-Ribose) Polymerase Activity. Nat. Commun. 2014, 5, 4426 10.1038/ncomms5426. 25043379 PMC4123609

[ref20] Bartlett E.; Bonfiglio J. J.; Prokhorova E.; Colby T.; Zobel F.; Ahel I.; Matic I. Interplay of Histone Marks with Serine ADP-Ribosylation. Cell Rep. 2018, 24, 3488–3502. 10.1016/j.celrep.2018.08.092. 30257210 PMC6172693

[ref21] Hendriks I. A.; Larsen S. C.; Nielsen M. L. An Advanced Strategy for Comprehensive Profiling of ADP-Ribosylation Sites Using Mass Spectrometry-Based Proteomics. Mol. Cell. Proteomics 2019, 18, 1010–1026. 10.1074/mcp.TIR119.001315. 30798302 PMC6495254

[ref22] Larsen S. C.; Hendriks I. A.; Lyon D.; Jensen L. J.; Nielsen M. L. Systems-Wide Analysis of Serine ADP-Ribosylation Reveals Widespread Occurrence and Site-Specific Overlap with Phosphorylation. Cell Rep. 2018, 24, 2493–2505. 10.1016/j.celrep.2018.07.083. 30157440

[ref23] Voorneveld J.; Rack J. G. M.; Ahel I.; Overkleeft H. S.; van der Marel G. A.; Filippov D. V. Synthetic α- and β-Ser-ADP-Ribosylated Peptides Reveal α-Ser-ADPr as the Native Epimer. Org. Lett. 2018, 20, 4140–4143. 10.1021/acs.orglett.8b01742. 29947522 PMC6038095

[ref24] Kliza K. W.; Liu Q.; Roosenboom L. W. M.; Jansen P. W. T. C.; Filippov D. V.; Vermeulen M. Reading ADP-Ribosylation Signaling Using Chemical Biology and Interaction Proteomics. Mol. Cell 2021, 81, 4552–4567. 10.1016/j.molcel.2021.08.037. 34551281

[ref25] Cohen M. S. Catching Mono- and Poly-ADP-Ribose Readers with Synthetic ADP-Ribose Baits. Mol. Cell 2021, 81, 4351–4353. 10.1016/j.molcel.2021.10.016. 34739826

[ref26] Voorneveld J.; Kloet M. S.; Wijngaarden S.; Kim R. Q.; Moutsiopoulou A.; Verdegaal M.; Misra M.; Đikić I.; van der Marel G. A.; Overkleeft H. S.; Filippov D. V.; van der Heden van Noort G. J. Arginine ADP-Ribosylation: Chemical Synthesis of Post-Translationally Modified Ubiquitin Proteins. J. Am. Chem. Soc. 2022, 144, 20582–20589. 10.1021/jacs.2c06249. 36318515 PMC9673145

[ref27] Kistemaker H. A. V.; Nardozza A. P.; Overkleeft H. S.; van der Marel G. A.; Ladurner A. G.; Filippov D. V. Synthesis and Macrodomain Binding of Mono-ADP-Ribosylated Peptides. Angew. Chem., Int. Ed. 2016, 55, 10634–10638. 10.1002/anie.201604058. 27464500

[ref28] Moyle P. M.; Muir T. W. Method for the Synthesis of Mono-ADP-Ribose Conjugated Peptides. J. Am. Chem. Soc. 2010, 132, 15878–15880. 10.1021/ja1064312. 20968292 PMC3010531

[ref29] Zhu A.; Li X.; Bai L.; Zhu G.; Guo Y.; Lin J.; Cui Y.; Tian G.; Zhang L.; Wang J.; Li X. D.; Li L. Biomimetic α-Selective Ribosylation Enables Two-Step Modular Synthesis of Biologically Important ADP-Ribosylated Peptides. Nat. Commun. 2020, 11, 5600 10.1038/s41467-020-19409-1. 33154359 PMC7645758

[ref30] Li L.; Li Q.; Ding S.; Xin P.; Zhang Y.; Huang S.; Zhang G. ADP-Ribosyl-N3: A Versatile Precursor for Divergent Syntheses of ADP-Ribosylated Compounds. Molecules 2017, 22, 1346 10.3390/molecules22081346. 28805740 PMC6152188

[ref31] Minnee H.; Rack J. G. M.; van der Marel G. A.; Overkleeft H. S.; Codée J. D. C.; Ahel I.; Filippov D. V. Mimetics of ADP-Ribosylated Histidine through Copper(I)-Catalyzed Click Chemistry. Org. Lett. 2022, 24, 3776–3780. 10.1021/acs.orglett.2c01300. 35587229 PMC9171823

[ref32] Liu Q.; Kistemaker H. A. V.; Bhogaraju S.; Dikic I.; Overkleeft H. S.; van der Marel G. A.; Ovaa H.; van der Heden van Noort G. J.; Filippov D. V. A. General Approach Towards Triazole-Linked Adenosine Diphosphate Ribosylated Peptides and Proteins. Angew. Chem., Int. Ed. 2018, 57, 1659–1662. 10.1002/anie.201710527. 29215186

[ref33] Oppenheimer N. J. Structural Determination and Stereospecificity of the Choleragen-Catalyzed Reaction of NAD + with Guanidines. J. Biol. Chem. 1978, 253, 4907–4910. 10.1016/S0021-9258(17)34632-X. 209022

[ref34] Ono T.; Kasamatsu A.; Oka S.; Moss J. The 39-KDa Poly(ADP-Ribose) Glycohydrolase ARH3 Hydrolyzes O-Acetyl-ADP-Ribose, a Product of the Sir2 Family of Acetyl-Histone Deacetylases. Proc. Natl. Acad. Sci. U.S.A. 2006, 103, 16687–16691. 10.1073/pnas.0607911103. 17075046 PMC1636516

[ref35] Stevens L. A.; Kato J.; Kasamatsu A.; Oda H.; Lee D.-Y.; Moss J. The ARH and Macrodomain Families of α-ADP-Ribose-Acceptor Hydrolases Catalyze α-NAD + Hydrolysis. ACS Chem. Biol. 2019, 14, 2576–2584. 10.1021/acschembio.9b00429. 31599159 PMC8388552

[ref36] Oka S.; Kato J.; Moss J. Identification and Characterization of a Mammalian 39-KDa Poly(ADP-Ribose) Glycohydrolase. J. Biol. Chem. 2006, 281, 705–713. 10.1074/jbc.M510290200. 16278211

[ref37] Rack J. G. M.; Palazzo L.; Ahel I. (ADP-Ribosyl)Hydrolases: Structure, Function, and Biology. Genes Dev. 2020, 34, 263–284. 10.1101/gad.334631.119. 32029451 PMC7050489

[ref38] Boren B. C.; Narayan S.; Rasmussen L. K.; Zhang L.; Zhao H.; Lin Z.; Jia G.; Fokin V. V. Ruthenium-Catalyzed Azide–Alkyne Cycloaddition: Scope and Mechanism. J. Am. Chem. Soc. 2008, 130, 8923–8930. 10.1021/ja0749993. 18570425

[ref39] Empting M.; Avrutina O.; Meusinger R.; Fabritz S.; Reinwarth M.; Biesalski M.; Voigt S.; Buntkowsky G.; Kolmar H. “Triazole Bridge”: Disulfide-Bond Replacement by Ruthenium-Catalyzed Formation of 1,5-Disubstituted 1,2,3-Triazoles. Angew. Chem., Int. Ed. 2011, 50, 5207–5211. 10.1002/anie.201008142. 21544910

[ref40] Gold H.; van Delft P.; Meeuwenoord N.; Codée J. D. C.; Filippov D. V.; Eggink G.; Overkleeft H. S.; van der Marel G. A. Synthesis of Sugar Nucleotides by Application of Phosphoramidites. J. Org. Chem. 2008, 73, 9458–9460. 10.1021/jo802021t. 18991380

[ref41] Štimac A.; Kobe J. An Improved Preparation of 2,3,5-Tri-O-Acyl-β-d-Ribofuranosyl Azides by the Lewis Acid-Catalysed Reaction of β-d-Ribofuranosyl Acetates and Trimethylsilyl Azide: An Example of Concomitant Formation of the α Anomer by Trimethylsilyl Triflate Catalysis. Carbohydr. Res. 1992, 232, 359–365. 10.1016/0008-6215(92)80068-C.

[ref42] Yan M.; Lo J. C.; Edwards J. T.; Baran P. S. Radicals: Reactive Intermediates with Translational Potential. J. Am. Chem. Soc. 2016, 138, 12692–12714. 10.1021/jacs.6b08856. 27631602 PMC5054485

[ref43] Mao Y.; Zhao W.; Lu S.; Yu L.; Wang Y.; Liang Y.; Ni S.; Pan Y. Copper-Catalysed Photoinduced Decarboxylative Alkynylation: A Combined Experimental and Computational Study. Chem. Sci. 2020, 11, 4939–4947. 10.1039/D0SC02213F. 34122950 PMC8159226

[ref44] Smith J. M.; Qin T.; Merchant R. R.; Edwards J. T.; Malins L. R.; Liu Z.; Che G.; Shen Z.; Shaw S. A.; Eastgate M. D.; Baran P. S. Decarboxylative Alkynylation. Angew. Chem., Int. Ed. 2017, 56, 11906–11910. 10.1002/anie.201705107. PMC5792189 28636185

[ref45] Pradere U.; Roy V.; McBrayer T. R.; Schinazi R. F.; Agrofoglio L. A. Preparation of Ribavirin Analogues by Copper- and Ruthenium-Catalyzed Azide-Alkyne 1,3-Dipolar Cycloaddition. Tetrahedron 2008, 64, 9044–9051. 10.1016/j.tet.2008.07.007. 34321698 PMC8315236

[ref46] Voorneveld J.; Rack J. G. M.; van Gijlswijk L.; Meeuwenoord N. J.; Liu Q.; Overkleeft H. S.; van der Marel G. A.; Ahel I.; Filippov D. V. Molecular Tools for the Study of ADP-Ribosylation: A Unified and Versatile Method to Synthesise Native Mono-ADP-Ribosylated Peptides. Chem. – Eur. J. 2021, 27, 10621–10627. 10.1002/chem.202100337. 33769608 PMC8360141

[ref47] Caron J.; Lepeltier E.; Reddy L. H.; Lepêtre-Mouelhi S.; Wack S.; Bourgaux C.; Couvreur P.; Desmaële D. Squalenoyl Gemcitabine Monophosphate: Synthesis, Characterisation of Nanoassemblies and Biological Evaluation. Eur. J. Org. Chem. 2011, 2011, 2615–2628. 10.1002/ejoc.201100036.

[ref48] Kistemaker H. A. V.; Meeuwenoord N. J.; Overkleeft H. S.; van der Marel G. A.; Filippov D. V. Solid-Phase Synthesis of Oligo-ADP-Ribose. Curr. Protoc. Nucleic Acid Chem. 2016, 64, 4.68.1–4.68.27. 10.1002/0471142700.nc0468s64. 31820580

[ref49] Hananya N.; Daley S. K.; Bagert J. D.; Muir T. W. Synthesis of ADP-Ribosylated Histones Reveals Site-Specific Impacts on Chromatin Structure and Function. J. Am. Chem. Soc. 2021, 143, 10847–10852. 10.1021/jacs.1c05429. 34264659

[ref50] Rack J. G. M.; Ahel I. A Simple Method to Study ADP-Ribosylation Reversal: From Function to Drug Discovery. Methods Mol. Biol. 2023, 2609, 111–132. 10.1007/978-1-0716-2891-1_8. 36515833

[ref51] Palazzo L.; Thomas B.; Jemth A.-S.; Colby T.; Leidecker O.; Feijs K. L. H.; Zaja R.; Loseva O.; Puigvert J. C.; Matic I.; Helleday T.; Ahel I. Processing of Protein ADP-Ribosylation by Nudix Hydrolases. Biochem. J. 2015, 468, 293–301. 10.1042/BJ20141554. 25789582 PMC6057610

