# Supplementary material for: Four of a Kind: A Complete Collection of ADP-Ribosylated Histidine Isosteres Using Cu(I)- and Ru(II)-Catalyzed Click Chemistry
Source: J Org Chem. 2023 Jul 19;88(15):10801–9. doi: 10.1021/acs.joc.3c00827 (PMC10407933; doi:10.1021/acs.joc.3c00827)
Supplement: Supplementary file 1 — jo3c00827_si_001.pdf [file jo3c00827_si_001.pdf]

Supporting Information

of

Four of a kind: a complete collection of ADP-ribosylated histidine isosteres using Cu(I)- and Ru(II)-catalyzed click chemistry

Hugo Minnee[1], Hayley Chung[1], Johannes Gregor Matthias Rack[2,3], Gijsbert A. van der Marel[1], Herman S. Overkleeft[1], Jeroen D.C. Codée[1], Ivan Ahel[2] and Dmitri V. Filippov[1*]

[1]Bio-Organic Synthesis, Leiden Institute of Chemistry, Leiden University
P.O. Box 9502, 2300 RA Leiden, The Netherlands
[2]Sir William Dunn School of Pathology, University of Oxford,
South Parks Road, Oxford, OX1 3RE, UK
[3]Current address: MRC Centre for Medical Mycology, School of Biosciences, University of Exeter, Geoffrey Pope Building, Exeter, EX4 4QD, UK

E-mail: filippov@chem.leidenuniv.nl

Table of Contents

Experimental procedures S2
Expression plasmids and protein purification S2
(ADP-ribosyl)hydrolase activity assay S2
Chemical synthesis S2
Solid phase peptide synthesis S3
On-resin phosphorylation, pyrophosphate construction and final deprotection S3
Chemical degradation assay S11
References S16
NMR spectra S19

Experimental procedures

Expression plasmids and protein purification

The construction of the expression plasmids and the purification procedures were described earlier.[1–4] Briefly, expression plasmids were transferred into Rossetta (DE3) cells and grown at 37 °C to an OD_{600} of 0.6 in LB medium supplemented with 1% (w/v) D-glucose and appropriate antibiotics. For (ADP-ribosyl)hydrolases (ARH1 and ARH3) the medium was further enriched by addition of 2 mM $MgSO_4$. Expression was induced with 0.4 mM isopropyl β-D-1- thioglactopyranoside (IPTG) and cultured were allowed to grow overnight at 17 °C. Cultures were harvested by centrifugation, pellets resuspended in lysis buffer (50 mM TrisHCl [pH 8], 500 mM NaCl and 25 mM imidazole) and stored at -20 °C until use. Proteins were purified by Ni^{2+}-NTA chromatography (Jena Bioscience) according to the manufacturer's protocol using the following buffers: all buffers contained 50 mM TrisHCl (pH 8) and 500 mM NaCl; additionally, the lysis buffer contained 25 mM, the washing buffer 40 mM, and the elution buffer 500 mM imidazole. Proteins were dialyzed overnight against 50 mM TrisHCl (pH 8), 200 mM NaCl, 1 mM dithiothreitol and 5% (v/v) glycerol and stored at -80 °C. For the purification of ARH1 and ARH3 all purification buffers were additionally supplemented with 10 mM $MgCl_2$.

(ADP-ribosyl)hydrolase activity assay

The peptide demodification assay was described earlier.[5,6] Briefly, peptide concentrations for the assay were estimated using absorbance at λ_{260nm} using the molar extinction coefficient of ADP-ribose (15,400 M^{-1} cm^{-1}). 10 µM indicated peptide were demodified by incubation with 0.5 µM hydrolase for 60 min at 30 °C in assay buffer (50 mM TrisHCl [pH 8], 200 mM NaCl, 10 mM MgCl2, 1 mM dithiothreitol and 0.2 µM human NUDT5)[5]. Reactions were stopped and analysed by performing the AMP-Glo™ assay (Promega) according to the manufacturer's protocol. Luminescence was recorded on a SpectraMax M5 plate reader (Molecular Devices) and data analysed with GraphPad Prism 7. Control reactions were carried out in absence of peptide.

Chemical synthesis

All chemicals were used as received unless stated otherwise. Boc-L-aspartic acid α-benzyl ester **4** was obtained from Chem Impex (catalog# 00390) and L-propargylglycine was obtained from Santa Cruz (catalog# SC-286102A). Dowex 50WX8 hydrogen form (100-200 mesh) was purchased at Sigma Aldrich and washed with H_2SO_4 (5 M, 3x) and MeOH (3x) prior to use. Molecular sieves were flamedried (3x) in vacuo before use. Solvents were dried over activated 4Å molsieves for 24 h except for MeCN and MeOH which were dried over 3Å molsieves. A solution of HCl (0.2 M in HFIP) was freshly prepared prior to the reaction by dissolving HCl (37%, 0.1 ml) to HFIP (5.9 ml). Reactions were performed under N_2 atmosphere unless stated otherwise. A Julabo FT902 cryostat was used for low temperature glycosylation reactions. Reaction mixtures were concentrated under reduced pressure using rotary evaporators at 40-45 ˚C unless state otherwise. Microwave syntheses were performed with the Biotage® Initiator+ microwave system using the 2.0-5.0 ml microwave vial types sealed with microwave caps (Screening devices, product# 130-3531) under the 'normal' absorption level setting. Reactions were monitored by thin layer chromatography (TLC) analysis using silica gel 60 F254 coated aluminum sheets from Merck. TLC plates were visualized with ultraviolet light (254 nm) or sprayed with H_2SO_4 (20% v/v in MeOH), potassium permanganate (1 g $KMnO_4$, 5 g K_2CO_3, in 200 ml H_2O) or ceric ammonium molybdate (1 g $Ce(NH_4)_4(SO_4)_4{\bullet}2H_2O$, 2.5 g $(NH_4)_6Mo_7O_{24}{\bullet}4H_2O$, 10 ml H_2SO_4 in 90 ml H_2O). Infrared (IR) values are reported in cm^{-1}. ^1H NMR, ^{13}C NMR and ^{31}P NMR spectra were recorded on Bruker AV-300 (300 mHz), AV-400 (400 mHz) or AV-500 (500 mHz) spectrometer. ^{13}C NMR spectra are acquired via the attached proton test (APT) experiment and are presented with even signals (C_q and CH_2) pointing upwards and odd signals (CH and CH_3) pointing downwards. The chemical shifts are noted as δ-values in parts per million (ppm) relative to the tetramethylsilane signal (δ = 0 ppm) or solvent signal of D_2O (δ = 4.79 ppm) for ^1H NMR and relative to the solvent signal of $CDCl_3$ (δ = 77.16 ppm) for ^{13}C NMR. Phosphorylation reactions were monitored with ^{31}P NMR using an acetone-D_6 insert for a locking signal and the resulting spectra were indirectly calibrated with H_3PO_4.

Structural assignments were made with additional information from gCOSY, gHSQC, and gHMBC experiments HRMS samples were prepared in either MeOH, MeCN or milliQ grade H_2O with an approximate concentration of 1 mM and measured on a Thermo Scientific LTQ Orbitrap XL.

Solid phase peptide synthesis

Fmoc-Asp(*t*-Bu)-OH, Fmoc-Val-OH, Fmoc-Pro-OH, Fmoc-Leu-OH, Fmoc-Gly-OH, Fmoc-Ala-OH and Fmoc-Phe-OH were all obtained from Merck Novabiochem. Boc-Thr(tBu)-OH was acquired from BLD pharmatech GmbH. Lysine(Boc) was purchased pre-loaded on tentagel® S AC resin from RAPP Polymere GmbH.

The Fmoc-N-Ala-Gly-Leu-Val-Val-Pro-Val-Asp-Lys-S AC linked Tentagel® sequence was prepared using a Liberty Blue peptide synthesizer via 9-fluorenylmethoxycarbonyl (Fmoc) based solid phase peptide chemistry at a 250 µmol scale. A 4 fold excess of the amino acids relative to the resin loaded amino acid was added in each prolongation step. A total of 4 equivalents of the additives Diisopropylcarbodiimide (DIC) and OxymaPure were added simultaneously. The coupling was established in the microwave reaction chamber at 90 °C for 2.5 minutes. After each coupling the peptide was subjected three consecutive times to a 20 v/v% piperidine solution in DMF at 90 °C for 1 minute to remove the Fmoc protection group.

The Fmoc-N-Ala-Gly-Leu-Val-Val-Pro-Val-Asp-Lys-Tentagel® sequence was completed at a 50 µmol scale by hand in a fritted syringe. After each step the resin was rinsed with the corresponding solvent (3x 3 ml) unless stated otherwise. Fmoc proctecting groups were removed by treatment with piperidine twice (10v/v% in DMF, 3 ml) for 3 and 7 min. Ribosylated amino acids **9**, **11**, **17** and **19** (0.1 mmol, 2 eq.) were coupled overnight in the presence DIPEA (40 µl, 4.5 eq.) and HCTU (0.1 mmol, 2 eq.) in DMF (3 ml). Fmoc-Phe-OH and Boc-Thr(*t*-Bu)-OH (0.25 mmol, 5 eq) were coupled for 45 min with HCTU (0.25 mmol, 5 eq) and DIPEA (90 µl, 10 eq.) in DMF (3 ml).

On-resin phosphorylation, pyrophosphate construction and final deprotection

TBDPS deprotection was achieved by treating the resin (50 µmol) with HF•pyridine (70 wt%, 1 ml) in pyridine (3 ml) twice for 45 min. The resin was washed with DMF (3x 3ml), DCM (3x 5 ml), Et_2O (3x 5 ml) and anhydrous MeCN (3x 3 ml) and flushed with nitrogen to minimize traces of water. Subsequently, the desilylated intermediate was treated with $(FmO)_2PN(i\text{-}Pr)_2$ (0.25 mmol, 5 eq.)[7] and ETT (0.25 mmol, 5 eq.) in anhydrous MeCN (3 ml) for 30 min. CSO (1 mmol, 20 eq.) in anhydrous MeCN (2 ml) was added and the resin was shaken again for 30 min. Then the newly introduced phosphate was deprotected with DBU (10v/v% in DMF, 2 ml) twice for 15 min and thoroughly washed with DCM (3x 5 ml), Et_2O (3x 5 ml) and anhydrous MeCN (3x 3 ml) before treating with either **30**[8] or **31**[9] (0.2 mmol, 4 eq.) in the presence of ETT (0.4 mmol, 8 eq.) in anhydrous MeCN (3 ml) for 30 min. The P(III)-P(V) intermediate was oxidized again with CSO (1 mmol, 20 eq.) in anhydrous MeCN (2 ml) for 30 min. The resin was subsequently shaken with DBU (10v/v% in anhydrous DMF, 2 ml) twice for 10 min each to remove the cyanoethyl group. The modified oligopeptide was cleaved from the resin using TFA/TIS/DCM (50:50:2.5, 4 ml) for 1 h. The solution was poured into a Falcon® tube containing ice cold Et_2O (40 ml) and the resulting suspension was centrifuged (5 min, 3000 RCF) using an Eppendorf centrifuge 5702 followed by removal of the supernatant. The partially protected precursors of **23** and **24** were stirred overnight in NH_4OH (28wt%, 8 ml) to remove benzoyl and *iso*-butyryl protecting groups, after which the solution was diluted with milliQ grade H_2O (5 ml) and washed with Et_2O (4x 10 ml). The H_2O fraction was collected, concentrated under reduced pressure and the resulting crude residues were subjected to preparative RP-HPLC (NH_4OAc buffered system). In contrast, crude residues of **28** and **29** were neutralized with NH_4OH (28wt%, 5 ml), diluted with milliQ grade H_2O (5 ml) and washed with Et_2O (4x 10 ml). The water layer was then collected, concentrated under reduced pressure and subjected to preparative RP-HPLC.

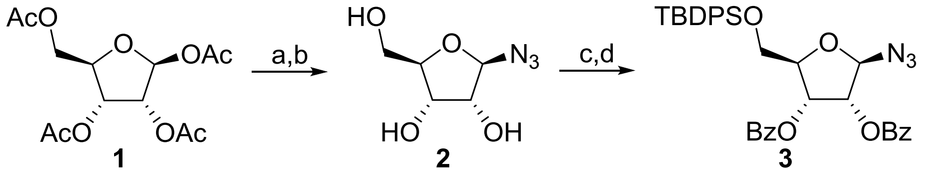

Scheme S1. Preparation of β-configured azido-ribofuranoside **3** using neighboring group participation. Reagents and conditions: a) $TMSN_3$, $SnCl_4$, DCM, rt, 1.5 h (97%). b) NaOMe, MeOH, rt, 1.5 h (92%). c) TBDPSCl, pyridine, rt, 16 h. d) BzCl, pyridine, rt, 2 h (82% over 2 steps).

1-Azido-2,3-bis-*O*-benzoyl-5-*O*-*tert*-butyldiphenylsilyl-β-D-ribo-furanoside (3).

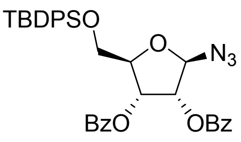

Compound **2**[10,11] (7.0 g, 40 mmol) was co-evaporated with dioxane (3x) and dissolved in anhydrous pyridine (150 ml). TBDPSCl (15.0 ml, 56.0 mmol) was added and the reaction mixture was stirred overnight at rt. BzCl (14.0 ml, 120 mmol) was added and the yellow suspension was stirred for 2h before it was quenched with H_2O (5 ml). The mixture was diluted with EtOAc (150 ml) and washed with $NaHCO_3$ (sat., 3x 40 ml). The combined organic fractions were dried over $MgSO_4$, filtered and concentrated under reduced pressure. Purification of the crude residue by silica gel column chromatography (pentane/Et_2O = 85:15) provided title compound **3** (20.5 g, 33.0 mmol, 82%) as a clear oil. $\mathbf{R_f}$ = 0.5 (pentane/EtOAc = 20:80). **^{1}H NMR** (300 MHz, $CDCl_3$): δ 8.13 (dd, *J* = 13.6, 7.0 Hz, 4H), 8.04 – 7.77 (m, 4H), 7.66 – 7.33 (m, 12H), 6.14 (dd, J = 5.8, 4.4 Hz, 1H), 5.88 – 5.78 (m, 2H), 4.71 (dt, J = 6.3, 3.4 Hz, 1H), 4.20 (dd, *J* = 11.5, 3.3 Hz, 1H), 4.09 (dd, *J* = 11.5, 3.6 Hz, 1H),, 1.32 (s, 9H). **^{13}C{^{1}H} NMR** (75 MHz, $CDCl_3$): δ 165.1, 165.0, 135.7, 135.6, 135.6, 135.5, 134.8, 133.5, 133.3, 132.8, 132.7, 129.9, 129.8, 129.8, 129.7, 129.6, 129.4, 129.0, 128.9, 128.4, 128.3, 127.9, 127.8, 127.7, 127.7, 127.7, 127.6, 93.0, 82.9, 75.4, 71.5, 63.3, 26.7, 26.6, 19.1. **HRMS** $[C_{35}H_{35}N_3O_6Si + NH_4]^+$ = 639.2629 found, 639.2633 calculated; $[C_{35}H_{35}N_3O_6Si + Na]^+$ = 644.2184 found, 644.2187 calculated.

Boc-L-Asp(O-hydroxyphtalamide)-OBn (5).

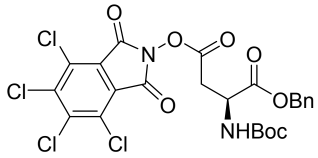

A solution of commercially available **4** (6.47 g, 20.0 mmol), *N*-hydroxy-tetrachlorophtalimide (7.28 g, 24.2 mmol) and DMAP (0.25 g, 2.0 mmol) in anhydrous DCM (200 ml) was purged with nitrogen. DIC (4.0 ml, 25.8 mmol) was added dropwise and the yellow suspension was stirred overnight at rt. The reaction mixture was filtered, washed with additional DCM and concentrated under reduced pressure. Purification of the crude residue by silica gel column chromatography (DCM/acetone = 97.5:2.5) yielded title compound **5** (9.86 g, 15.9 mmol, 80%) as a yellow solid. $\mathbf{R_f}$ = 0.4 (pentane/EtOAc = 80:20). **^{1}H NMR** (300 MHz, $CDCl_3$): δ 7.40 – 7.29 (m, 5H), 5.52 (d, J = 8.0 Hz, 1H), 5.33 – 5.13 (m, 2H), 4.74 (dt, J = 8.4, 4.7 Hz, 1H), 3.38 (dd, *J* = 17.2, 4.8 Hz, 1H), 3.27 (dd, *J* = 17.0, 5.0 Hz, 1H). , 1.45 (s, 9H). **^{13}C{^{1}H} NMR** (75 MHz, $CDCl_3$): δ 169.8, 167.0, 157.2, 155.3, 141.3, 135.0, 130.7, 128.7, 128.7, 124.7, 68.2, 50.1, 34.2, 28.4. **HRMS** $[C_{24}H_{20}Cl_4N_2O_8 + Na]^+$ = 628.9835 found, 628.9837 calculated.

Boc-L-Pra-OBn (6).

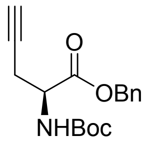

Nickel/ligand solution: $NiCl{\cdot}6H_2O$ (52 mg, 0.22 mmol) and 4,4-dimethoxy-2,2'-bipyridine (43 mg, 0.20 mmol) were dissolved in anhydrous DMF (10 ml) and stirred under argon atmosphere for 30 min prior to use.

Ethynylzinc chloride solution: a solution of $ZnCl_2$ (0.74 g, 5.4 mmol) and LiCl (0.24 g, 5.6 mmol) in anhydrous THF (5.5 ml) was stirred homogenous in a flame-dried round-bottom flask. Ethynylmagnesium bromide solution (0.5 M, 11 ml) was added and stirred for 30 minutes.

Compound **7** (0.62 g, 1.0 mmol) was co-evaporated with toluene and kept under argon atmosphere. The nickel/ligand solution (9.5 ml) and ethynylzinc chloride solution (11 ml) were added in quick succession and the resulting black mixture was stirred overnight. The reaction was quenched with HCl (1 M, 10 ml), diluted

with H_2O (60 ml) and extracted with Et_2O (3x 50 ml). The combined organic fractions were washed with brine (3x 100 ml), dried over $MgSO_4$ and concentrated under reduced pressure. Purification of the crude residue by silica gel column chromatography (pentane/EtOAc = 90:10) yielded title compound **6** (0.22 g, 0.74 mmol, 73%) as an off-white oil. $\mathbf{R_f}$ = 0.6 (pentane/EtOAc = 80:20). The obtained spectra were in full accordance with literature experimental data.[12]

Fmoc-L-Pra-OBn (7).

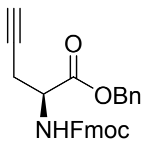

Compound **6** (0.38 g, 1.2 mmol) was dissolved in TFA/DCM (1:1, 6.1 ml) and stirred for 2h at rt. The yellow suspension was concentrated under reduced pressure and the crude residue was co-evaporated with toluene (3x) before dissolving in sat. $NaHCO_3$/MeCN (1:1, 12 ml). Fmoc-OSu (1.1 g, 3.0 mmol) was added and the reaction mixture was stirred overnight at rt. The yellow suspension was extracted with DCM (3x 50 ml). The combined organic fractions were washed with brine (3x 100 ml), dried over $MgSO_4$, filtered and concentrated under reduced pressure. Purification of the crude residue by silica gel column chromatography (pentane/EtOAc = 20:80) yielded title compound **7** (0.47 g, 1.1 mmol, 91%) as a white solid. $\mathbf{R_f}$ = 0.6 (pentane/Et_2O = 50:50). **^{1}H NMR** (500 MHz, $CDCl_3$) δ 7.72 (d, J = 7.5 Hz, 2H), 7.58 (d, J = 7.5 Hz, 2H), 7.39 – 7.24 (m, 10H), 5.76 (d, J = 8.3 Hz, 1H), 5.26 – 5.15 (m, 2H)., 4.58 (dt, J = 8.8, 4.8 Hz, 1H), 4.37 (d, J = 7.3 Hz, 2H), 4.20 (t, J = 7.2 Hz, 1H), 2.77 – 2.74 (m, 1H), 2.01 (t, J = 2.5 Hz, 1H). **^{13}C{^{1}H} NMR** (126 MHz, $CDCl_3$): δ 170.4, 155.9, 144.1, 143.9, 141.5, 135.3, 128.8, 128.8, 128.5, 128.0, 127.3, 125.4, 120.2, 78.5, 72.2, 67.9, 67.5, 52.7, 47.3, 23.0. **HRMS** $[C_{27}H_{23}NO_4 + H]^+$ = 426.1695 found, 426.1700 calculated; $[C_{27}H_{23}NO_4 + NH_4]^+$ = 443.1961 found, 443.1965 calculated; $[C_{27}H_{23}NO_4 + Na]^+$ = 448.1519 found, 448.1514 calculated.

Fmoc-His*(1’-N(τ)-2’,3’-bis-*O*-benzoyl-5’-*O*-*tert*-diphenylsilyl-β-D-ribofuranosyl)-OBn (8).

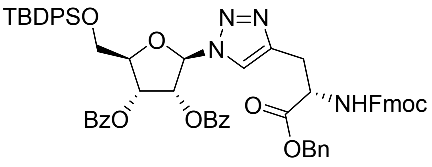

To a solution of **3** (0.19 g, 0.30 mmol) and **7** (0.10 g, 0.30 mmol) in DMF (3 ml) were added $CuSO_4{\cdot}5H_2O$ (75 mg, 0.30 mmol) and sodium ascorbate (0.18 g, 0.90 mmol) consecutively. The green suspension was stirred at rt for 1 h, diluted with H_2O (30 ml) and extracted with Et_2O (3x 20 ml). The combined organic fractions were washed with brine (1x 20 ml), dried over $MgSO_4$, filtered and concentrated under reduced pressure. Purification of the crude residue by silica gel column chromatography (pentane/EtOAc = 70:30) yielded title compound **8** (0.25 g, 0.24 mmol, 80%) as an off-white solid. $\mathbf{R_f}$ = 0.4 (pentane/EtOAc = 70:30). **^{1}H NMR** (500 MHz, $CDCl_3$): δ7.96 (dd, *J* = 8.4, 1.3 Hz, 2H), 7.92 (dd, *J* = 8.4, 1.3 Hz, 2H) , 7.73 (d, *J* = 7.6 Hz, 2H) , 7.69 – 7.65 (m, 2H), 7.60 – 7.54 (m, 3H) , 7.55 – 7.48 (m, 2H), 7.43 – 7.23 (m, 20H), 6.46 (d, J = 5.2 Hz, 1H), 6.10 (t, J = 5.3 Hz, 1H), 6.04 (dd, J = 5.4, 4.1 Hz, 1H), 5.92 (d, J = 8.1 Hz, 1H), 5.21 – 5.10 (m, 2H), 4.73 – 4.65 (m, 1H), 4.56 (q, *J* = 3.3 Hz, 1H), 4.36 (dd, J = 10.5, 7.3 Hz, 1H), 4.26 (dd, J = 10.5, 7.4 Hz, 1H), 4.19 (t, J = 7.4 Hz, 1H), 4.04 (dd, *J* = 11.7, 3.0 Hz, 1H), 4.00 (dd, *J* = 11.7, 3.3 Hz, 1H), 3.25 – 3.08 (m, 2H), 1.12 (s, 9H). **^{13}C{^{1}H} NMR** (126 MHz, $CDCl_3$): δ 171.0., 165.3, 164.9, 156.0, 144.0, 143.9, 143.3, 141.3, 141.3, 135.7, 135.6, 135.4, 133.8, 133.7, 133.0, 132.3, 130.2, 130.1, 130.0, 129.8, 129.0, 128.6, 128.6, 128.6, 128.5, 128.5, 128.4, 128.0, 127.7, 127.7, 127.2, 125.4, 125.3, 121.0, 120.0, 90.2, 84.2, 75.2, 71.9, 67.4, 67.3, 63.7, 53.5, 47.2, 29.8, 28.0, 27.0, 19.4. **HRMS** $[C_{62}H_{58}N_4O_{10}Si + H]+$ = 1047.3982 found, 1047.3995 calculated.

Fmoc-His*(1’-N(τ)-2’,3’-bis-*O*-benzoyl-5’-*O*-*tert*-diphenylsilyl-β-D-ribofuranosyl)-OH (9).

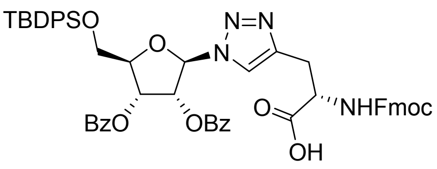

Compound **8** (2.8 g, 2.7 mmol) was dissolved in MeOH (54 ml) and purged with nitrogen for 15 minutes before adding Pd/C (10 wt%, 0.58 g, 0.50 mmol). The reaction mixture was bubbled with H_2 for 1 h and stirred overnight at rt under H_2 atmosphere. AcOH (0.5 ml) was added and the suspension was filtered over celite and washed with additional MeOH. The filtrate was concentrated under pressure and co-evaporated with toluene (3x). Purification of the

crude residue by silica gel column chromatography (DCM/actone + 1% AcOH: 95:5) yielded title compound **9** (2.06 g, 2.15 mmol, 80%) as a white foam. $\mathbf{R_f}$ = 0.3 (DCM/acetone + 1% AcOH = 90:10). **^{1}H NMR** (400 MHz, $CDCl_3$): δ 11.53 (bs, 1COOH), 7.92 (t, *J* = 7.5 Hz, 4H), 7.73 (s, 1H), 7.67 (d, *J* = 7.7 Hz, 6H), 7.58 – 7.40 (m, 4H), 7.39 – 7.19 (m, 14H), 6.51 (d, *J* = 4.8 Hz, 1H), 6.22 – 6.13 (m, 1H), 6.06 (t, *J* = 4.7 Hz, 1H), 4.70 (q, *J* = 6.0 Hz, 1H), 4.55 – 4.53 (m, 1H), 4.32 (dd, *J* = 10.4, 7.4 Hz, 1H), 4.21 (dd, *J* = 10.4, 7.5 Hz, 1H), 4.13 (t, *J* = 7.2 Hz, 1H), 4.06 – 3.92 (m, 2H), 3.41 – 3.25 (m, 2H), 1.12 (s, 9H). **^{13}C{^{1}H} NMR** (101 MHz, $CDCl_3$): δ 173.2, 165.1, 164.8, 156.1, 143.8, 143.7, 143.0, 141.1, 141.1, 135.6, 135.4, 133.6, 132.8, 132.1, 130.0, 129.8, 129.7, 129.0, 128.8, 128.4, 128.4, 128.2, 127.9, 127.9, 127.6, 127.1, 125.3, 125.2, 121.8, 119.8, 90.3, 84.2, 75.2, 71.7, 67.1, 63.4, 53.4, 46.9, 27.7, 26.9, 19.2. **HRMS** $[C_{55}H_{52}N_4O_{10}Si + H]^+$ = 957.3521 found, 957.3526 calculated.

Fmoc-His*(1'-N(π)-2',3'-bis-*O*-benzoyl-5'-*O*-*tert*-diphenylsilyl-β-D-ribofuranosyl)-OBn (10).

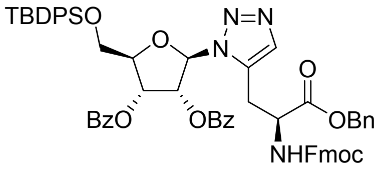

Compounds **3** (0.32 g, 0.50 mmol) and **7** (0.14 g, 0.32 mmol) were co-evaporated with toluene (3x) before dissolving in anhydrous THF (5 ml). The solution was purged with nitrogen gas for 15 min before adding C_p*RuCl(COD) (5 mg, 0.01 mmol). The yellow solution was heated to 100 °C in the microwave for 5 min and concentrated under reduced pressure. Purification of the crude residue by silica gel column chromatography (pentane/EtOAc = 60:40) yielded title compound **10** (0.30 g, 0.31 mmol, 85%) as a white solid. $\mathbf{R_f}$ = 0.5 (pentane/EtOAc = 70:30). **^{1}H NMR** (600 MHz, $CDCl_3$): δ 7.95 (d, *J* = 6.9 Hz, 2H), 7.90 (dd, *J* = 8.3, 1.3 Hz, 2H), 7.71 (d, *J* = 7.6 Hz, 2H), 7.64 – 7.55 (m, 4H), 7.54 – 7.41 (m, 4H), 7.40 – 7.30 (m, 7H), 7.31 – 7.21 (m, 13H), 6.49 – 6.44 (m, 1H), 6.32 (d, *J* = 3.3 Hz, 1H), 6.17 (t, *J* = 5.6 Hz, 1H), 5.67 (d, *J* = 7.4 Hz, 1H), 5.23 – 5.07 (m, 2H), 4.73 (q, *J* = 6.4 Hz, 1H), 4.59 (q, *J* = 5.0 Hz, 1H), 4.32 (dd, *J* = 10.5, 7.1 Hz, 1H), 4.12 (dd, *J* = 10.5, 7.2 Hz, 1H), 4.03 (t, *J* = 7.2 Hz, 1H), 3.94 – 3.84 (m, 2H), 3.45 (dd, *J* = 15.9, 5.8 Hz, 1H), 3.30 (dd, *J* = 15.6, 6.2 Hz, 1H), 1.02 (s, 9H). **^{13}C{^{1}H} NMR** (151 MHz, CDCl3): δ 170.2, 165.2, 165.1, 155.8, 143.6, 143.6, 141.3, 135.7, 135.6, 134.7, 133.8, 133.6, 133.5, 133.1, 133.0, 132.9, 129.9, 129.8, 129.8, 129.8, 129.1, 128.8, 128.8, 128.8, 128.7, 128.6, 128.5, 127.8, 127.8, 127.8, 127.8, 127.1, 125.1, 120.0, 88.0, 84.1, 75.1, 72.2, 68.0, 67.3, 64.0, 53.2, 47.0, 26.8, 19.2. **HRMS** $[C_{62}H_{58}N_4O_{10}Si + H]^+$ = 1047.3992 found, 1047.3995 calculated.

Fmoc-His*(1'-N(π)-2',3'-bis-*O*-benzoyl-5'-*O*-*tert*-diphenylsilyl-β-D-ribofuranosyl)-OH (11).

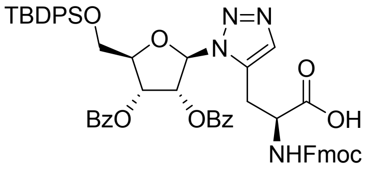

Compound **10** (5.0 g, 4.8 mmol) was dissolved in MeOH (95 ml) and purged with nitrogen for 15 minutes before adding Pd/C (10 wt%, 1.0 g, 0.90 mmol). The suspension was purged with H_2 and stirred overnight at rt overnight under H_2 atmosphere. AcOH (1 ml) was added and the reaction mixture was filtered over celite and washed with additional MeOH. The filtrate was concentrated under pressure. Purification of the crude residue by silica gel column chromatography (DCM/actone + 1% AcOH: 95:5) yielded title compound **11** (4.1 g, 4.3 mmol, 90%) as a yellowish foam. $\mathbf{R_f}$ = 0.3 (DCM/acetone + 1% AcOH = 90:10). **^{1}H NMR** (400 MHz, $CDCl_3$): δ 12.49 (bs, 1COOH), 7.91 (dd, *J* = 13.6, 7.7 Hz, 4H), 7.77 (s, 1H), 7.68 – 7.60 (m, 4H), 7.58 (d, 2H), 7.47 – 7.05 (m, 18H), 6.69 – 6.62 (m, 1H), 6.52 (d, *J* = 3.3 Hz, 1H), 6.25 (t, *J* = 5.4 Hz, 1H), 6.17 (d, *J* = 6.6 Hz, 1H), 4.77 (d, *J* = 6.5 Hz, 1H), 4.63 (q, *J* = 4.5 Hz, 1H), 4.31 – 4.22 (m, 1H), 4.11 – 3.83 (m, 4H), 3.58 (d, *J* = 11.9 Hz, 1H), 3.41 (d, *J* = 11.8 Hz, 1H), 1.04 (s, 9H). **^{13}C{^{1}H} NMR** (101 MHz, $CDCl_3$) δ 172.5, 165.2, 156.1, 143.7, 143.6, 141.2, 135.7, 135.6, 134.4, 133.6, 133.5, 133.4, 132.9, 132.8, 129.9, 129.8, 129.8, 129.1, 129.1, 128.8, 128.4, 128.3, 127.8, 127.8, 127.2, 127.1, 125.4, 125.2, 112.0, 88.1, 84.2, 74.9, 72.0, 67.4, 63.7, 53.3, 47.0, 26.8, 25.9, 19.2. **HRMS** $[C_{55}H_{52}N_4O_{10}Si + H]^+$ = 957.3520 found, 957.3526 calculated.

Fmoc-L-Pra-OH (13).

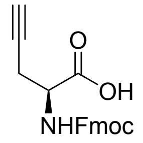

Commercially available L-propargylglycine **12** (150 mg, 1.33 mmol) and $NaHCO_3$ (278 mg, 3.32 mmol) were dissolved in milliQ water (7.4 ml). A solution of Fmoc-OSu (537 mg, 1.59 mmol) in THF (7.4 ml) was added to the reaction mixture and the suspension was stirred overnight at rt. The clear solution was acidified with HCl (1 M) until pH=1, diluted with H_2O (20 ml) and extracted with EtOAc (3x 30 ml). The combined organic fractions were washed with brine (30 ml), dried over $MgSO_4$, filtered and concentrated under reduced pressure. Purification of the crude residue by silica gel column chromatography (pentane/EtOAc + 1% AcOH= 80:20 → 60:40) yielded title compound **13** (445 mg, 1.33 mmol, quant.) as a white solid. $\mathbf{R_f}$ = 0.4 (pentane/EtOAc +1% AcOH = 50:50). The obtained spectra were in full accordance with literature experimental data.[13]

Fmoc-L-Pra-OAllyl (14).

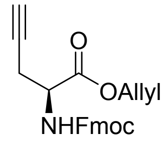

To a suspension of **13** (250 mg, 0.745 mmol) and DMAP (18 mg, 0.15 mmol) in anhydrous DCM (7.5 ml) was added allyl alcohol (0.080 ml, 1.1 mmol) followed by DIC (1.0 M, 1.1 ml, 1.1 mmol). The resulting solution was stirred at rt for 45 min and concentrated under reduced pressure. Purification of the crude residue by silica gel column chromatography (Pentane/Et_2O = 95:5 → 90:10) yielded title compound **14** (228 mg, 0.601 mmol, 81%) as a white solid. $\mathbf{R_f}$ = 0.5 (Pentane/EtOAc = 80:20). **^{1}H NMR** (300 MHz, $CDCl_3$): δ 7.75 (d, *J* = 7.5 Hz, 2H), 7.60 (d, *J* = 7.4 Hz, 2H), 7.44 – 7.24 (m, 4H), 6.00 – 5.81 (m, 1H), 5.72 (d, *J* = 8.3 Hz, 1H), 5.40 – 5.20 (m, 2H), 4.77 – 4.61 (m, 2H), 4.65 – 4.46 (m, 1H), 4.39 (d, *J* = 7.2 Hz, 2H), 4.23 (t, *J* = 7.2 Hz, 1H), 2.80 (dd, *J* = 4.9, 2.7 Hz, 2H), 2.07 (t, *J* = 2.6 Hz, 1H). **^{13}C{^{1}H} NMR** (75 MHz, $CDCl_3$): δ 170.1, 155.7, 143.9, 143.8, 141.4, 131.4, 127.8, 127.2, 125.2, 120.1, 119.1, 78.4, 72.1, 67.3, 66.5, 52.5, 47.2, 22.8. **HRMS** $[C_{23}H_{21}NO_4 + H]^+$ = 376.15406 found, 376.15433 calculated; $[C_{23}H_{21}NO_4 + NH_4]^+$ = 393.18088 found, 393.18058 calculated; $[C_{23}H_{21}NO_4 + Na]^+$ = 398.1363 found, 398.1360 calculated.

Fmoc-His*(1'-N(τ)-2',3'-bis-*O*-para-methoxybenzyl-5'-*O*-*tert*-diphenylsilyl-α-D-ribo-furanosyl)-OAllyl (16).

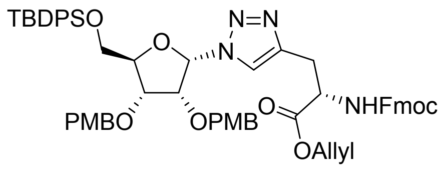

To a solution of **15**[11] (750 mg, 1.15 mmol) and **14** (560 mg, 1.49 mmol) in DMF (11.5 ml) were added $CuSO_4 \cdot 5H_2O$ (0.14 g, 0.57 mmol) and sodium ascorbate (0.68 g, 3.4 mmol) consecutively. The green suspension was stirred for 30 min at rt, diluted with water (100 ml) and extracted with Et_2O (3x 100 ml). The combined organic fractions were dried over $MgSO_4$, filtered and concentrated under reduced pressure. Purification of the crude residue by silica gel column chromatography (pentane/EtOAc = 90:10 → 70:30) yielded title compound **16** (1.10 g, 1.07 mmol, 93%) as a white foam. $\mathbf{R_f}$ = 0.6 (pentane/EtOAc = 60:40). **^{1}H NMR** (400 MHz, $CDCl_3$): δ 8.08 (s, 1H), 7.72 (d, *J* = 8.5 Hz, 2H), 7.65 – 7.54 (m, 6H), 7.47 – 7.32 (m, 8H), 7.27 (d, *J* = 1.2 Hz, 2H), 7.17 (d, *J* = 8.6 Hz, 2H), 7.03 (d, *J* = 8.6 Hz, 2H), 6.82 (dd, *J* = 19.4, 8.6 Hz, 4H), 6.55 (d, *J* = 6.0 Hz, 1H), 6.10 (d, *J* = 8.4 Hz, 1H), 5.90 – 5.75 (m, 1H), 5.29 – 5.13 (m, 2H), 4.75 (dt, *J* = 8.4, 5.3 Hz, 1H), 4.65 (d, *J* = 11.2 Hz, 1H), 4.63 – 4.52 (m, 2H), 4.53 – 4.43 (m, 2H), 4.41 – 4.18 (m, 5H), 4.24 – 4.16 (m, 2H), 3.77 (s, 3H), 3.77 – 3.69 (m, 4H), 3.58 (dd, *J* = 11.5, 2.4 Hz, 1H), 3.29 (d, *J* = 5.4 Hz, 2H), 0.99 (s, 9H). **^{13}C{^{1}H} NMR** (101 MHz, $CDCl_3$): δ 170.9, 159.5, 159.5, 156.1, 143.9, 143.9, 142.3, 141.2, 141.2, 135.6, 135.5, 132.9, 132.4, 131.6, 130.0, 129.7, 129.5, 129.4, 128.8, 127.9, 127.6, 127.2, 125.4, 125.3, 123.4, 120.0, 119.9, 118.7, 114.0, 113.9, 113.9, 89.1, 85.0, 77.4, 76.6, 72.7, 72.6, 67.3, 66.0, 63.8, 55.3, 55.2, 53.7, 47.1, 28.1, 26.8, 19.1. **HRMS** $[C_{60}H_{64}N_4O_{10}Si + H]^+$ = 1029.4458 found, 1029.4465 calculated; $[C_{60}H_{64}N_4O_{10}Si + Na]^+$ = 1051.4280 found, 1051.4284 calculated.

Fmoc-His*(1'-N(τ)-(2',3'-bis-*O*-para-methoxybenzyl-5'-*O*-*tert*-diphenylsilyl-α-D-ribo-furanosyl))-OH (17).

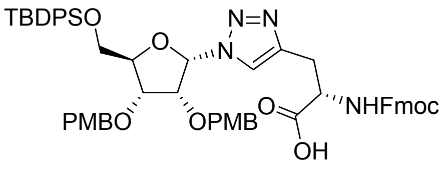

Compound **16** (1.10 g, 1.07 mmol) and DMBA (0.18 g, 1.2 mmol) were co-evaporated with toluene (3x) before dissolving in anhydrous DCM (22 ml). The solution was purged with argon for 15 min before adding $Pd(PPh_3)_4$ (25 mg, 0.021 mmol). After 1.5 h, the yellow solution was diluted with DCM (100 ml) and washed with citric acid (10wt%, 2x 100 ml). The H_2O layer was back-extracted with DCM (2x 75 ml. The combined organic fractions were dried over $MgSO_4$, filtered and concentrated under reduced pressure. Purification of the crude residue by silica gel column chromatography (DCM/acetone + 1% AcOH = 95:5 → 90:10) yielded title compound **17** (1.01 g, 1.02 mmol, 96%) as a white foam. **R$_f$** = 0.3 (DCM/acetone + 1% AcOH = 90:10). **^{1}H NMR** (400 MHz, $CDCl_3$): δ 10.92 (bs, COOH), 8.21 (s, 1H), 7.72 – 7.61 (m, 2H), 7.64 – 7.44 (m, 6H), 7.45 – 7.17 (m, 10H), 7.14 (d, *J* = 8.2 Hz, 2H), 7.02 (d, *J* = 8.2 Hz, 2H), 6.80 (dd, *J* = 20.2, 8.6 Hz, 2H), 6.53 (d, *J* = 5.9 Hz, 1H), 6.11 (d, *J* = 7.1 Hz, 1H), 4.74 (q, *J* = 5.9 Hz, 1H), 4.60 (d, *J* = 11.4 Hz, 1H), 4.52 – 4.39 (m, 2H), 4.38 – 4.27 (m, 3H), 4.22 – 4.07 (m, 4H), 3.76 – 3.63 (m, 7H), 3.58 – 3.52 (m, 1H), 3.47 – 3.40 (m, 2H), 0.97 (s, 9H). **^{13}C{^{1}H} NMR** (101 MHz, $CDCl_3$): δ 173.0, 159.5, 159.4, 156.0, 143.9, 143.8, 142.1, 141.1, 135.5, 135.4, 132.8, 132.4, 132.2, 132.2, 132.2, 132.1, 129.9, 129.6, 129.3, 128.7, 128.6, 128.5, 127.9, 127.8, 127.6, 127.6, 127.1, 127.1, 125.3, 125.3, 124.2, 119.8, 114.0, 113.9, 113.8, 89.3, 84.9, 76.4, 72.6, 72.5, 67.2, 63.7, 55.2, 55.1, 53.7, 47.0, 27.9, 26.8, 19.1. **HRMS** $[C_{57}H_{60}N_4O_{10}Si + H]^+$ = 989.4144 found, 989.4152 calculated.

Fmoc-His*(1'-N(π)-(2',3'-bis-*O*-para-methoxybenzyl-5'-*O*-*tert*-diphenylsilyl-α-D-ribo-furanosyl))-OAllyl (18).

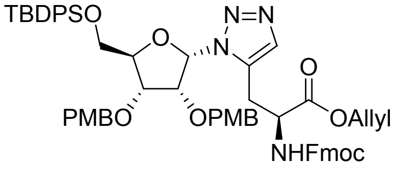

Compound **15** (1.07 g, 1.64 mmol) and **14** (1.23 g, 3.27 mmol) were co-evaporated with toluene (3x) before dissolving in anhydrous THF (25 ml). The solution was purged with argon gas for 15 min before adding C_p*RuCl(COD) (31 mg, 0.082 mmol). The yellow solution was heated to 100 °C in the microwave for 1 h and concentrated under reduced pressure. Purification of the crude residue by silica gel column chromatography (pentane/EtOAc = 90:10 → 70:30) yielded title compound **18** (1.01 g, 0.981 mmol, 60%) as a yellowish foam. **R$_f$** = 0.5 (pentane/EtOAc = 60:40). **^{1}H NMR** (500 MHz, $CDCl_3$): δ 7.72 (d, *J* = 7.5 Hz, 2H), 7.62 (td, *J* = 7.8, 1.5 Hz, 4H), 7.57 (s, 1H), 7.47 (dd, *J* = 13.1, 7.5 Hz, 2H), 7.42 – 7.21 (m, 10H), 7.15 (d, *J* = 8.4 Hz, 2H), 6.95 (d, *J* = 8.1 Hz, 2H), 6.80 (dd, *J* = 25.3, 8.6 Hz, 4H), 6.69 (d, *J* = 3.9 Hz, 1H), 5.82 – 5.70 (m, 1H), 5.65 (d, *J* = 8.2 Hz, 1H), 5.27 – 5.14 (m, 2H), 4.61 – 4.41 (m, 4H), 4.38 (dd, *J* = 7.6, 4.6 Hz, 1H), 4.35 – 4.29 (m, 2H), 4.26 (d, *J* = 7.4 Hz, 2H), 4.11 (t, *J* = 7.2 Hz, 1H), 4.05 – 3.92 (m, 3H), 3.81 – 3.68 (m, 7H), 3.45 (dd, *J* = 15.6, 8.9 Hz, 1H), 3.28 (dd, *J* = 15.5, 6.0 Hz, 1H), 1.02 (s, 9H). **^{13}C{^{1}H} NMR** (126 MHz, $CDCl_3$): δ 171.0, 159.6, 159.6, 155.7, 143.8, 143.8, 141.3, 141.3, 135.6, 135.6, 134.9, 134.4, 133.1, 132.7, 131.4, 129.9, 129.9, 129.9, 129.5, 129.2, 128.6, 127.9, 127.7, 127.1, 127.1, 125.1, 120.0, 119.0, 114.0, 113.9, 91.9, 82.5, 77.3, 76.3, 72.9, 72.6, 67.1, 66.1, 62.7, 55.3, 55.3, 53.7, 47.0, 26.9, 26.3, 19.3. **HRMS** $[C_{60}H_{64}N_4O_{10}Si + H]^+$ = 1029.44563 found, 1029.44645 calculated; $[C_{60}H_{64}N_4O_{10}Si + Na]^+$ = 1051.4282 found, 1051.4284 calculated.

Fmoc-His*(1'-N(π)-(2',3'-bis-*O*-para-methoxybenzyl-5'-*O*-*tert*-diphenylsilyl-α-D-ribo-furanosyl))-OH (19).

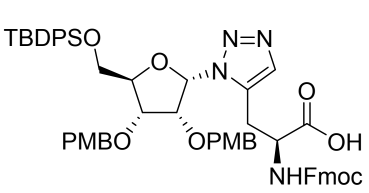

A solution of **18** (1.75 g, 1.70 mmol) and DMBA (0.29 g, 1.9 mmol) in anhydrous DCM (22 ml) was purged with argon for 15 min before adding $Pd(PPh_3)_4$ (39 mg, 0.034 mmol). After 1.5 h, the yellow solution was diluted with DCM (100 ml) and washed with citric acid (10wt%, 2x 100 ml). The H_2O layer was back-extracted with DCM (2x 75 ml. The combined organic fractions were dried over $MgSO_4$, filtered and concentrated under reduced pressure. Purification of the crude residue by silica gel column chromatography (DCM/acetone + 1% AcOH = 97.5:2.5 → 95:5) yielded title compound **19** (1.43 g, 1.45 mmol, 85%) as yellowish foam. **R$_f$** = 0.4 (DCM/acetone + 1% AcOH = 90:10). **^{1}H NMR** (500 MHz, $CDCl_3$): δ 9.76 (bs, COOH), 7.69 (d, *J* = 7.6 Hz, 2H), 7.66 – 7.56 (m, 5H), 7.51 – 7.44 (m, 2H), 7.44 – 7.35 (m, 2H), 7.36 – 7.27 (m, 6H), 7.26 – 7.17 (m, 2H), 7.12 (d, *J* = 8.2 Hz, 2H), 6.94 (d, *J* = 8.3 Hz, 2H), 6.77 (dd,

J = 22.6, 8.6 Hz, 4H), 6.62 (d, *J* = 3.4 Hz, 1H), 5.77 (d, *J* = 7.9 Hz, 1H), 4.60 (q, *J* = 7.4 Hz, 1H), 4.52 (d, *J* = 6.7 Hz, 1H), 4.40 (d, *J* = 11.5 Hz, 1H), 4.33 – 4.22 (m, 5H), 4.13 – 4.03 (m, 2H), 4.02 – 3.92 (m, 2H), 3.73 (s, 3H), 3.69 (s, 4H), 3.53 – 3.45 (m, 1H), 3.37 (dd, *J* = 15.6, 6.1 Hz, 1H), 1.00 (s, 9H). **$^{13}C\{^1H\}$ NMR** (126 MHz, $CDCl_3$): δ 173.6, 159.6, 159.5, 155.9, 143.8, 141.3, 141.3, 135.7, 135.6, 135.6, 135.2, 133.1, 132.6, 132.4, 132.4, 132.3, 132.2, 129.9, 129.6, 129.2, 128.8, 128.7, 128.6, 127.9, 127.7, 127.7, 127.2, 127.2, 125.2, 125.2, 119.9, 114.0, 113.9, 91.8, 82.6, 77.1, 76.1, 72.8, 72.4, 67.2, 62.9, 55.3, 55.3, 53.5, 47.1, 26.9, 26.4, 19.3. **HRMS** $[C_{57}H_{60}N_4O_{10}Si + H]^+$ = 989.4148 found, 989.4152 calculated.

β-N(τ)-ADPr-His*: TF[H*]GAGLVVPVDK (H* = Triazolyl ADPr) (23).

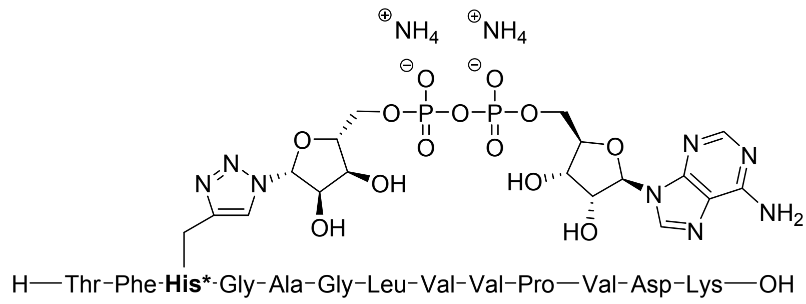

Title compound **23** was synthesized according to the general resin based procedure described above on a 50 µmol scale by incorporating 1,4-disubstituted triazole **9**. The 5'-phosphoryl-ribofuranose intermediate was coupled to adenosine amidite **30**.[8] Purification of the crude residue (50 µmol) by preparative HPLC (NH_4OAc buffer) and subsequent lyophilization yielded title compound **23** (24.7 mg, 13.1 µmol, 26%) as a fluffy white powder. The obtained spectra were in full accordance with previously reported experimental data.[11] **LC-MS** R_t = 5.30 min (10-50% $MeCN/H_2O$, TFA). **^1H NMR** (400 MHz, D_2O): δ 8.44 (s, 1H), 8.17 (s, 1H), 8.03 (s, 1H), 7.24 – 7.11 (m, 3H), 7.04 (d, *J* = 7.2 Hz, 2H), 6.01 (dd, *J* = 7.8, 5.2 Hz, 2H). **^{31}P NMR** (162 MHz, D_2O): δ -10.51, -10.63, -10.73, -10.86. **HRMS** $[C_{76}H_{118}N_{22}O_{30}P_2 + 2H]^{2+}$ = 941.4000 found, 941.4003 calculated; $[C_{76}H_{118}N_{22}O_{30}P_2 + 3H]^{3+}$ = 627.9360 found, 627.9359 calculated.

β-N(π)-ADPr-His*: TF[H*]GAGLVVPVDK (H* = Triazolyl ADPr) (24).

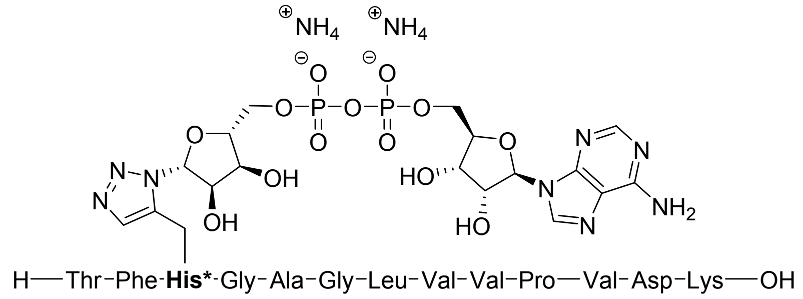

Title compound **24** was synthesized according to the general resin based procedure described above on a 50 µmol scale by incorporating 1,4-disubstituted triazole **11**. The 5'-phosphoryl-ribofuranose intermediate was coupled to adenosine amidite **30**.[8] Purification of the crude residue (50 µmol) by preparative HPLC (NH_4OAc buffer) and subsequent lyophilization yielded title compound **24** (8.0 mg, 4.3 µmol, 9%) as a fluffy white powder. **LC-MS** R_t = 4.41 min (10-50% $MeCN/H_2O$, TFA). **^1H NMR** (400 MHz, D_2O): δ 8.44 (s, 1H), 8.19 (s, 1H), 7.49 (s, 1H), 7.25 – 7.17 (m, *J* = 6.9, 6.5 Hz, 3H), 7.10 (d, *J* = 7.2 Hz, 2H), 6.04 (d, *J* = 5.5 Hz, 1H), 5.92 (d, *J* = 3.8 Hz, 1H). **^{31}P NMR** (162 MHz, D_2O): δ -10.67. **HRMS** $[C_{76}H_{118}N_{22}O_{30}P_2 + 2H]^{2+}$ = 941.40000 found, 941.4003 calculated; $[C_{76}H_{118}N_{22}O_{30}P_2 + 3H]^{3+}$ = 627.9362 found, 627.9359 calculated.

α-N(τ)-ADPr-His*: TF[H*]GAGLVVPVDK (H* = Triazolyl ADPr) (28).

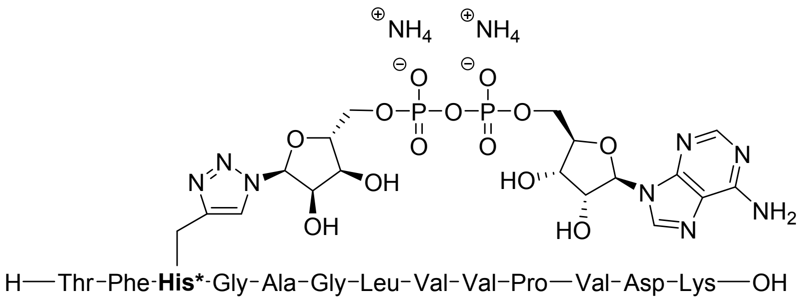

Title compound **28** was synthesized according to the general resin based procedure described above on a 50 µmol scale by incorporating 1,4-disubstituted triazole **17**. The 5'-phosphoryl-ribofuranose intermediate was coupled to adenosine amidite **31**.[9] Purification of the crude residue (50 µmol) by preparative HPLC (NH_4OAc buffer) and subsequent lyophilization yielded title compound **28** (33.2 mg, 17.7 µmol, 35%) as a fluffy white powder. The obtained spectra were in full accordance with previously reported experimental data.[11] **LC-MS** R_t = 4.42 min (10-50% MeCN/H_2O, TFA). **^{1}H NMR** (400 MHz, D_2O): δ 8.48 (s, 1H), 8.17 (s, 1H), 7.95pp (s, 1H), 7.27 – 7.16 (m, 3H), 7.10 (d, *J* = 8.0 Hz, 2H), 6.31 (d, *J* = 5.3 Hz, 1H), 6.07 (d, *J* = 5.8 Hz, 1H). **^{31}P NMR** (162 MHz, D_2O): δ -10.32, -10.45, -10.70, -10.83. **HRMS** $[C_{76}H_{118}N_{22}O_{30}P_2 + 2H]^{2+}$ = 941.4001 found, 941.4003 calculated; $[C_{76}H_{118}N_{22}O_{30}P_2 + 3H]^{3+}$ = 627.9361 found, 627.9359 calculated.

α-N(π)-ADPr-His*: TF[H*]GAGLVVPVDK (H* = Triazolyl ADPr) (29).

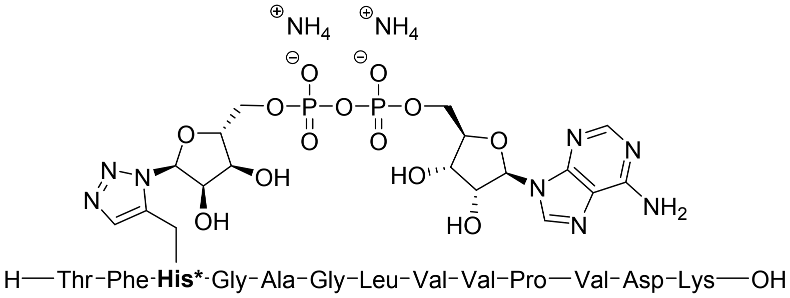

Title compound **29** was synthesized according to the general resin based procedure described above on a 50 µmol scale by incorporating 1,4-disubstituted triazole **19**. The 5'-phosphoryl-ribofuranose intermediate was coupled to adenosine amidite **31**.[8] Purification of half of the crude residue (25 µmol) by preparative HPLC (NH_4OAc buffer) and subsequent lyophilization yielded title compound **29** (11.9 mg, 6.37 µmol, 25%) as a fluffy white powder. **LC-MS** R_t = 4.38 min (10-50% MeCN/H_2O, TFA). **^{1}H NMR** (400 MHz, D_2O): δ 8.49 (s, 1H), 8.20 (s, 1H), 7.44 (s, 1H), 7.28 – 7.18 (m, 4H), 7.13 (d, *J* = 7.1 Hz, 2H), 6.35 (d, *J* = 5.2 Hz, 1H), 6.05 (d, *J* = 5.7 Hz, 1H). **^{31}P NMR** (162 MHz, D_2O): δ -10.42, -10.54, -10.64, -10.77. **HRMS** $[C_{76}H_{118}N_{22}O_{30}P_2 + 2H]^{2+}$ = 941.3998 found, 941.4003 calculated; $[C_{76}H_{118}N_{22}O_{30}P_2 + 3H]^{3+}$ = 627.9359 found, 627.9359 calculated.

Chemical degradation assay

A stock solution of peptides **23**, **24**, **28** and **29** (1 mg/ml) was prepared in milliQ grade water. The stock solution was mixed with an equal volume of an aqueous solution of TFA (0.2 M), NH_2OH (1 M) or NaOH (0.2 M) and shaken at rt. Samples (20 µl) were taken at various time points for LC-MS analysis. In case of TFA and NH_2OH, samples were simply diluted with milliQ grade water (80 µl) before injection. NaOH samples were acidified with TFA (0.2 M, 40 µl) and diluted with milliQ grade water (40 µl) prior to analysis. Peptide degradation was monitored by UV detection and mass fragments. The resulting peak areas in the UV-trace (260 nm) were quantified in Thermo Xcalibur Qual Browser using the "Auto Calc Initial Event" option. The time-course LC-MS data of **24** and **29** was imported to GraphPad Prism 9.0.0 and subjected to a "Nonlinear regression (curve fit)". Selecting the "Exponential (one-phase decay)" option provided a trendline including the coefficient of determination (R^2).

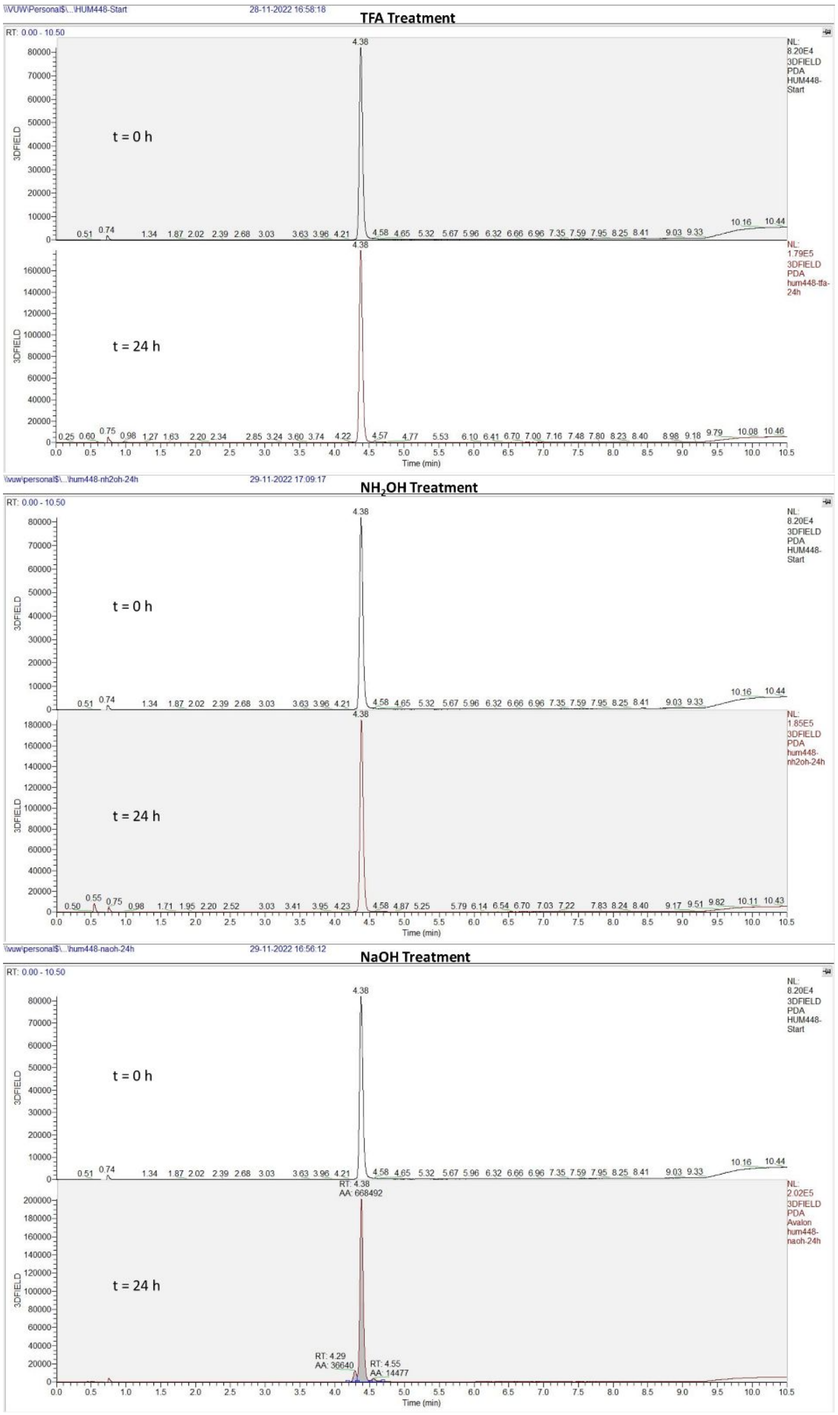

Figure S1. UV-trace (260 nm) of the obtained LC-MS spectra after treating β-configured 1,4-disubstituted triazole **23** with TFA (0.1 M, top) or NH_2OH (0.5 M, mid) or NaOH (0.1 M, bot) at various time points.

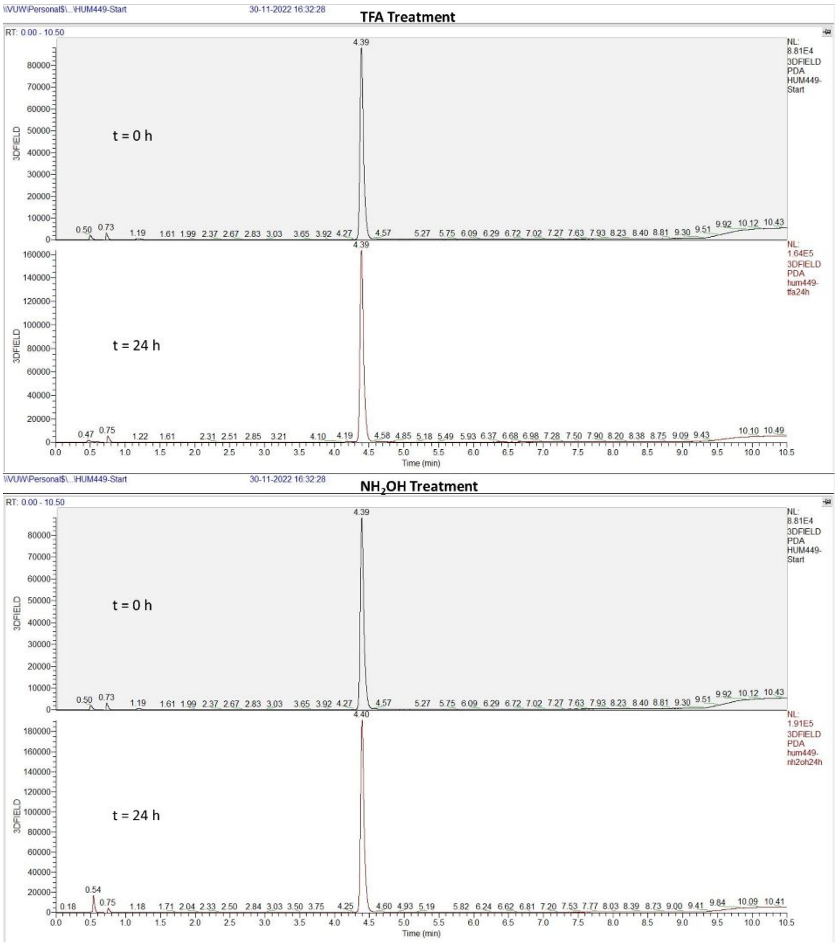

Figure S2. UV-trace (260 nm) of the obtained LC-MS spectra after treating β-configured 1,5-disubstituted triazole **24** with TFA (0.1 M, top) or NH_2OH (0.5 M, mid) at various time points.

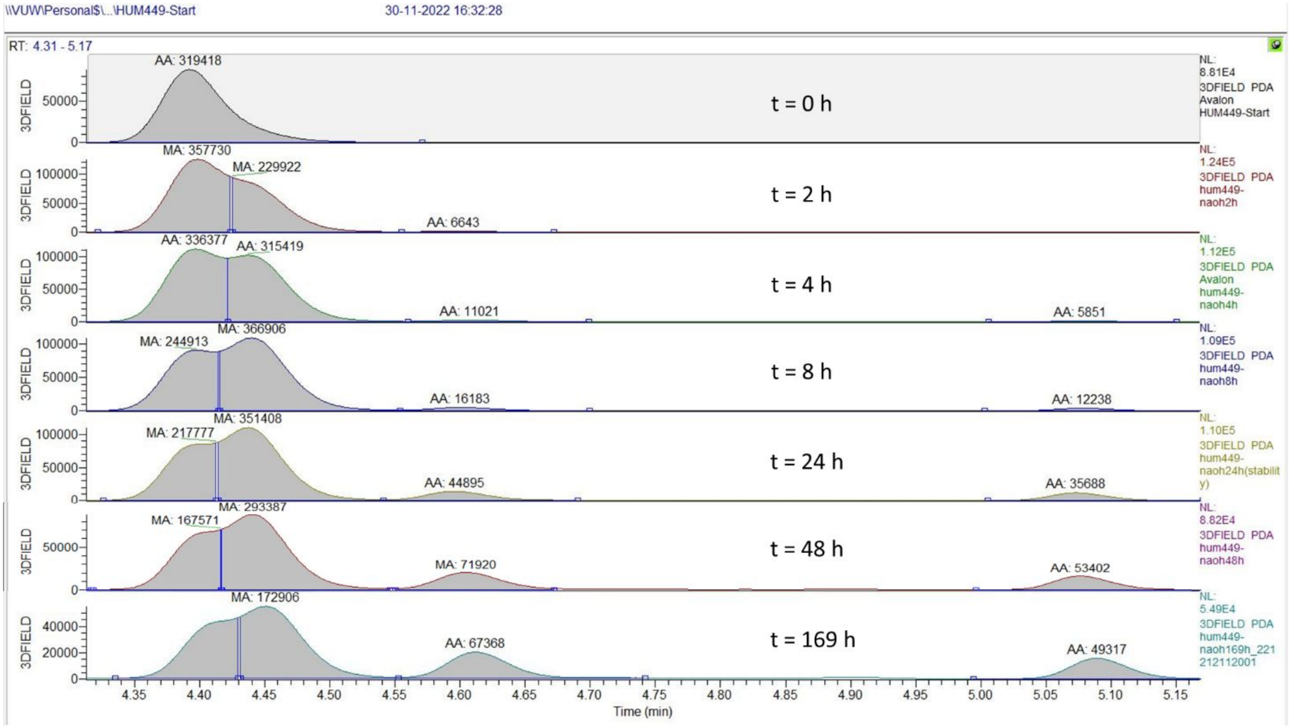

Figure S3. UV-trace (260 nm) of the obtained LC-MS spectra after treating β-configured 1,5-disubstituted triazole **24** with 0.1 M NaOH at various time points.

Table S1. Residual peptide **24** after treatment with NaOH (0.1 M) derived from the data presented in figure S3.

Time (h)	Intact peptide (%)	Accumulated isomers (%)
0	100	0
2	61	39
4	52	48
8	38	52
24	34	56
48	29	71
169	29	71

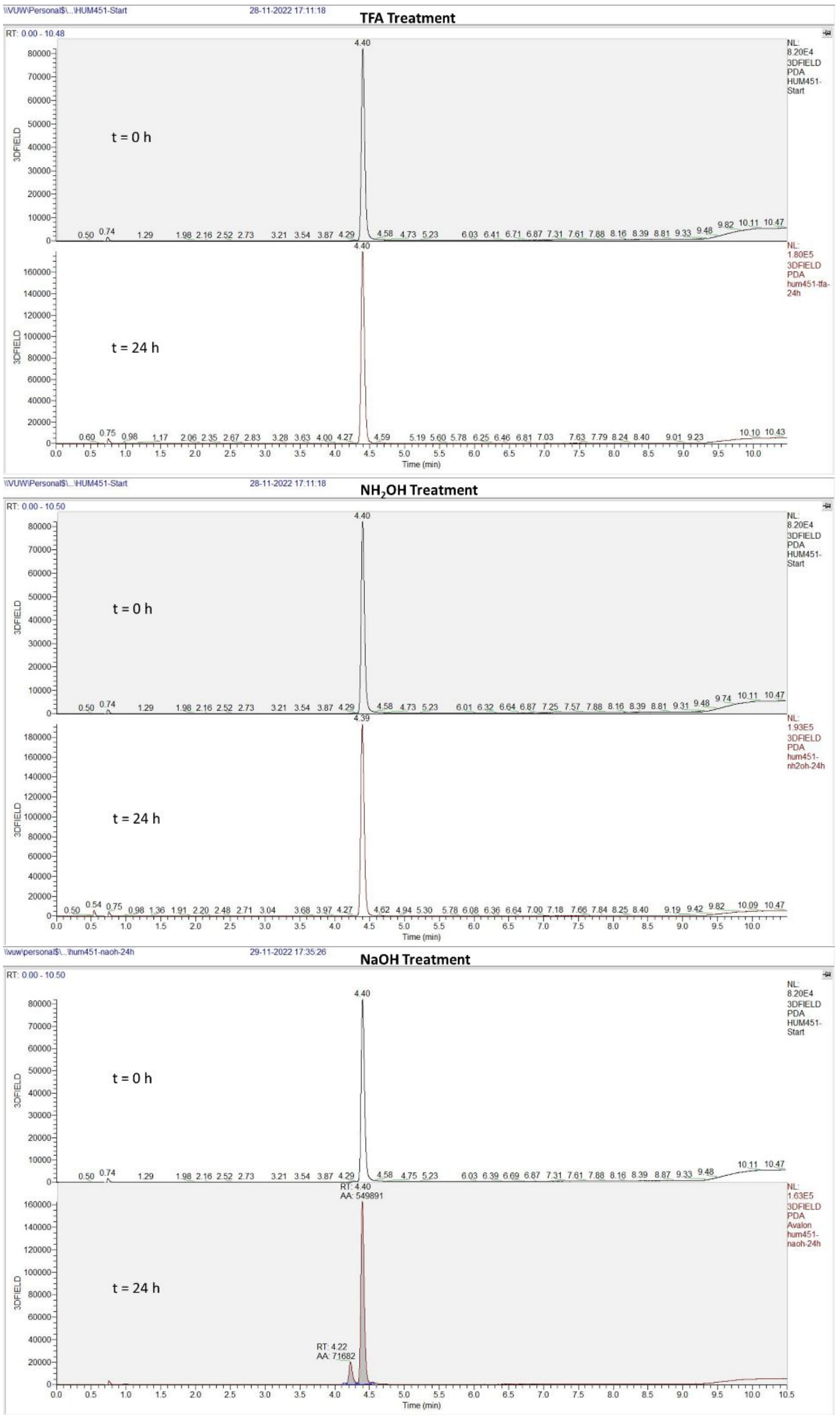

Figure S4. UV-trace (260 nm) of the obtained LC-MS spectra after treating α-configured 1,4-disubstituted triazole **28** with TFA (0.1 M, top) or NH_2OH (0.5 M, mid) or NaOH (0.1 M, bot) at various time points.

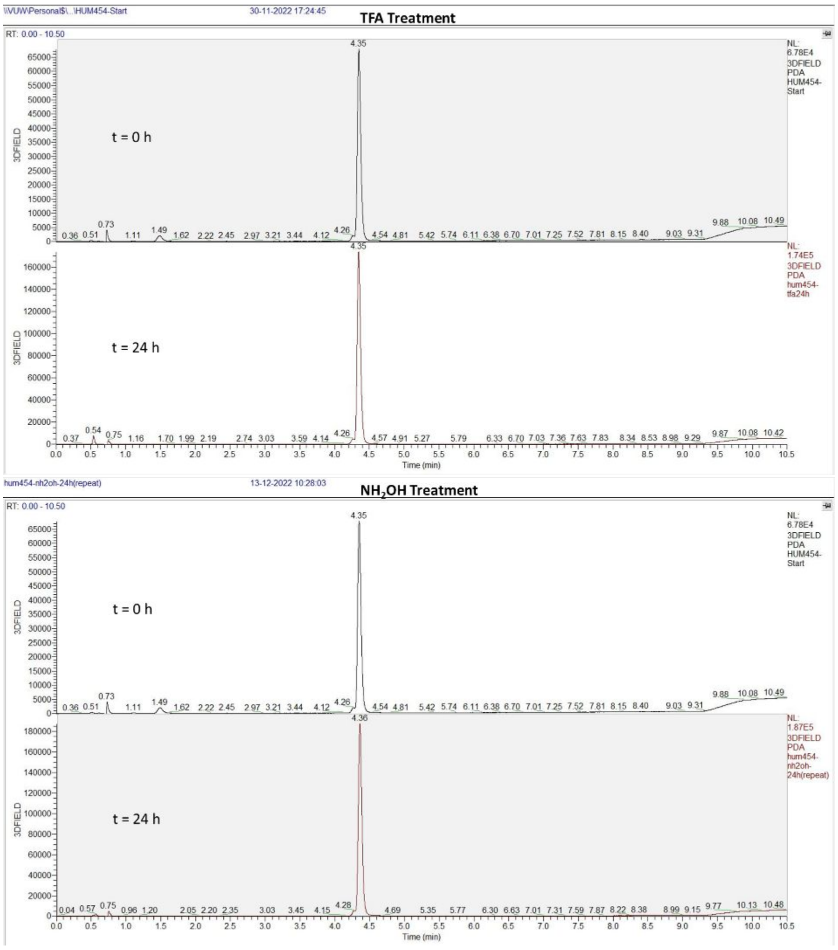

Figure S5. UV-trace (260 nm) of the obtained LC-MS spectra after treating α-configured 1,5-disubstituted triazole **29** with TFA (0.1 M, top) or NH_2OH (0.5 M, bot) at various time points.

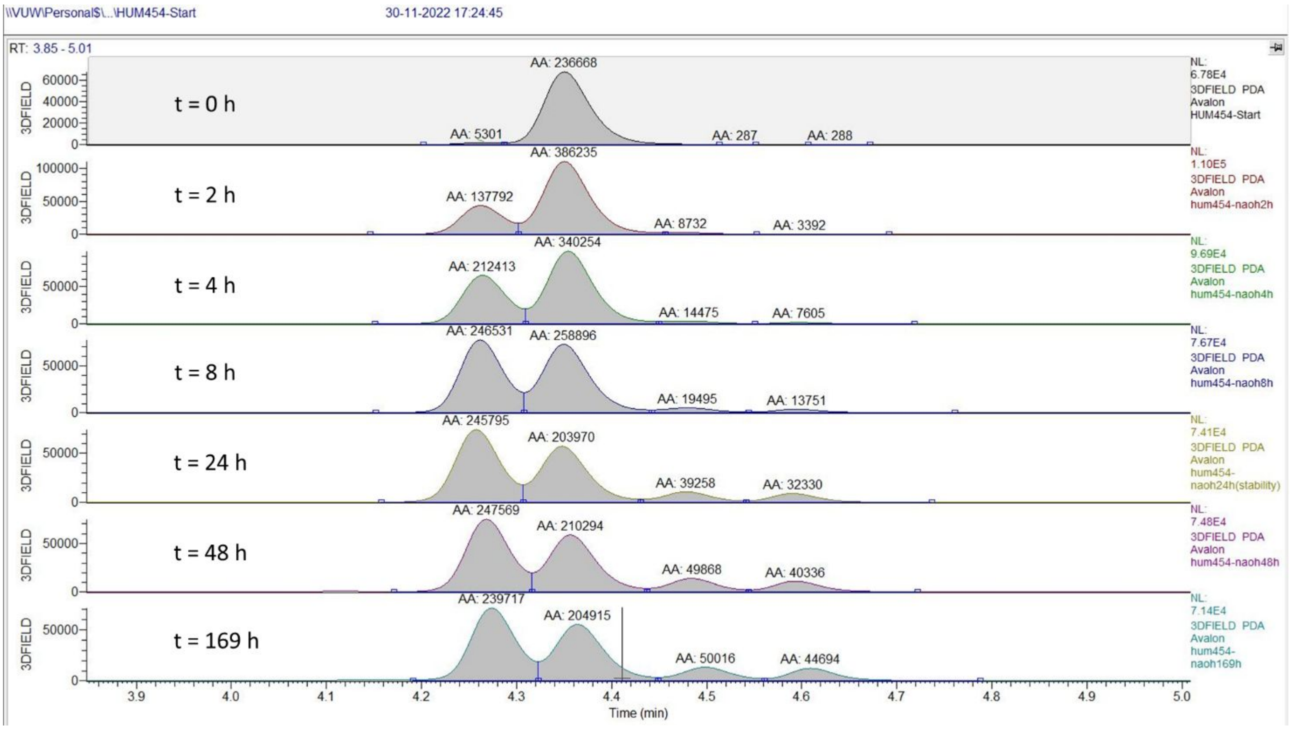

Figure S6. UV-trace (260 nm) of the obtained LC-MS spectra after treating α-configured 1,5-disubstituted triazole **29** with 0.1 M NaOH at various time points.

Table S2. Residual peptide **29** after treatment with NaOH (0.1 M) derived from the data presented in figure S6.

Time (h)	Intact peptide (%)	Accumulated isomers (%)
0	100	0
2	74	26
4	60	40
8	49	51
24	39	61
48	38	62
169	38	62

References

(1) Fontana, P.; Bonfiglio, J. J.; Palazzo, L.; Bartlett, E.; Matic, I.; Ahel, I. Serine ADP-Ribosylation Reversal by the Hydrolase ARH3. *Elife* **2017**, *6* (e28533), 1–20.

(2) Palazzo, L.; Thomas, B.; Jemth, A.-S.; Colby, T.; Leidecker, O.; Feijs, K. L. H.; Zaja, R.; Loseva, O.; Puigvert, J. C.; Matic, I.; Helleday, T.; Ahel, I. Processing of Protein ADP-Ribosylation by Nudix Hydrolases. *Biochem.* **2015**, *468* (2), 293–301.

(3) Slade, D.; Dunstan, M. S.; Barkauskaite, E.; Weston, R.; Lafite, P.; Dixon, N.; Ahel, M.; Leys, D.; Ahel, I. The Structure and Catalytic Mechanism of a Poly(ADP-Ribose) Glycohydrolase. *Nature* **2011**, *477* (7366), 616–620.

(4) Rack, J. G. M.; Ariza, A.; Drown, B. S.; Henfrey, C.; Bartlett, E.; Shirai, T.; Hergenrother, P. J.; Ahel, I. (ADP-Ribosyl)Hydrolases: Structural Basis for Differential Substrate Recognition and Inhibition. *Cell Chem. Biol.* **2018**, *25* (12), 1533-1546.e12.

(5) Voorneveld, J.; Rack, J. G. M.; Ahel, I.; Overkleeft, H. S.; van der Marel, G. A.; Filippov, D. V. Synthetic α- and β-Ser-ADP-Ribosylated Peptides Reveal α-Ser-ADPr as the Native Epimer. *Org. Lett.* **2018**, *20* (13), 4140–4143.

(6) Voorneveld, J.; Rack, J. G. M.; van Gijlswijk, L.; Meeuwenoord, N. J.; Liu, Q.; Overkleeft, H. S.; van der Marel, G. A.; Ahel, I.; Filippov, D. V. Molecular Tools for the Study of ADP-Ribosylation: A Unified and Versatile Method to Synthesise Native Mono-ADP-Ribosylated Peptides. *Chemistry* **2021**, *27* (41), 10621–10627.

(7) Caron, J.; Lepeltier, E.; Reddy, L. H.; Lepêtre-Mouelhi, S.; Wack, S.; Bourgaux, C.; Couvreur, P.; Desmaële, D. Squalenoyl Gemcitabine Monophosphate: Synthesis, Characterisation of Nanoassemblies and Biological Evaluation. *European Journal of Organic Chemistry* **2011**, *2011* (14), 2615–2628.

(8) Kistemaker, H. A. V.; Meeuwenoord, N. J.; Overkleeft, H. S.; Marel, G. A. van der; Filippov, D. V. Solid-Phase Synthesis of Oligo-ADP-Ribose. *Curr. Prot. Nucleic Acid Chem.* **2016**, *64* (1), 4.68.1-4.68.27.

(9) Hananya, N.; Daley, S. K.; Bagert, J. D.; Muir, T. W. Synthesis of ADP-Ribosylated Histones Reveals Site-Specific Impacts on Chromatin Structure and Function. *J. Am. Chem. Soc.* **2021**, *143* (29), 10847–10852.

(10) Štimac, A.; Kobe, J. An Improved Preparation of 2,3,5-Tri-O-Acyl-β-d-Ribofuranosyl Azides by the Lewis Acid-Catalysed Reaction of β-d-Ribofuranosyl Acetates and Trimethylsilyl Azide: An Example of Concomitant Formation of the α Anomer by Trimethylsilyl Triflate Catalysis. *Carbohydr. Res.* **1992**, *232* (2), 359–365.

(11) Minnee, H.; Rack, J. G. M.; van der Marel, G. A.; Overkleeft, H. S.; Codée, J. D. C.; Ahel, I.; Filippov, D. V. Mimetics of ADP-Ribosylated Histidine through Copper(I)-Catalyzed Click Chemistry. *Org. Lett.* **2022**, *24* (21), 3776–3780.

(12) Usuki, T.; Yamada, H.; Hayashi, T.; Yanuma, H.; Koseki, Y.; Suzuki, N.; Masuyama, Y.; Lin, Y. Y. Total Synthesis of COPD Biomarker Desmosine That Crosslinks Elastin. *Chem. Commun.* **2012**, *48* (26), 3233–3235.

(13) Byrne, C.; McEwan, P. A.; Emsley, J.; Fischer, P. M.; Chan, W. C. End-Stapled Homo and Hetero Collagen Triple Helices: A Click Chemistry Approach. *Chem. Commun.* **2011**, *47* (9), 2589–2591.

NMR spectra

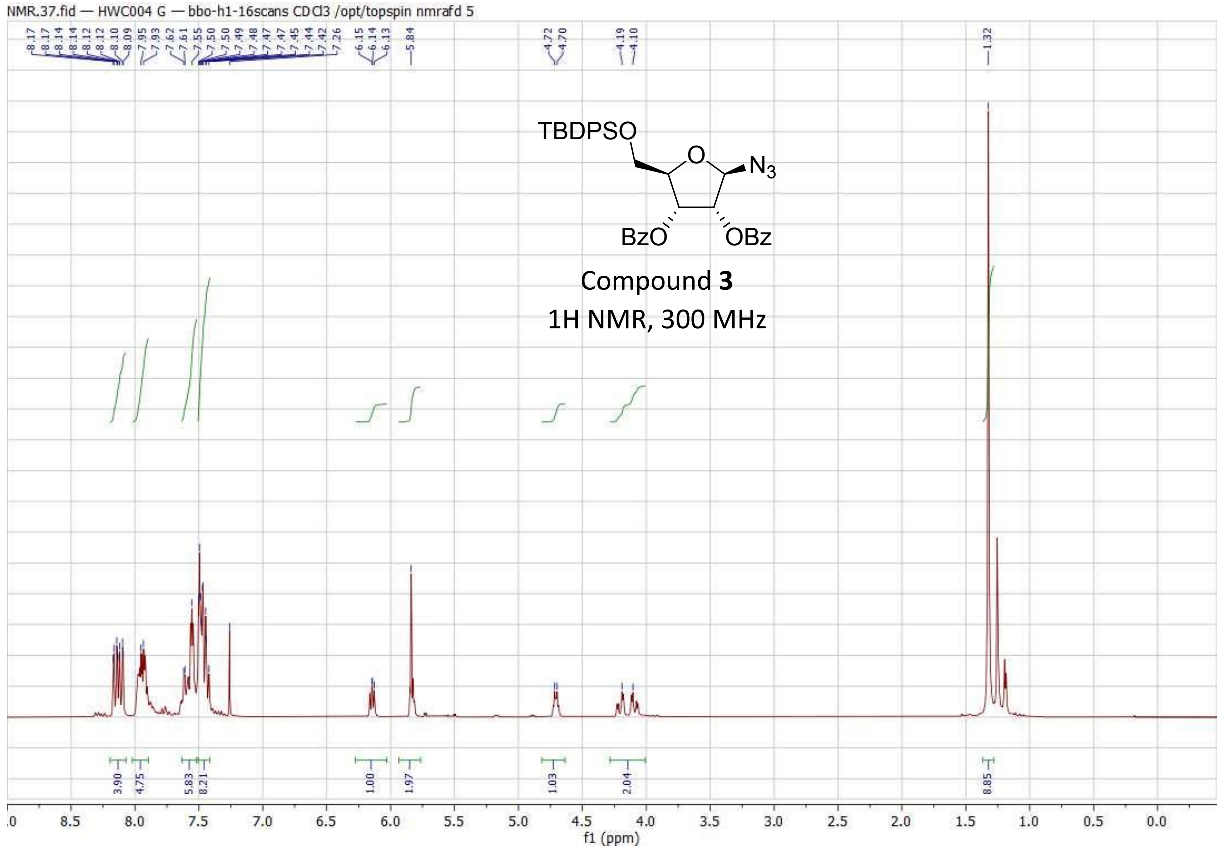
NMR.37.fid — HWC004 G — bbo-h1-16scans CDCl3 /opt/topspin nmrafd 5
TBDPSO
O
N3
BzO
OBz
Compound **3**
1H NMR, 300 MHz
f1 (ppm)

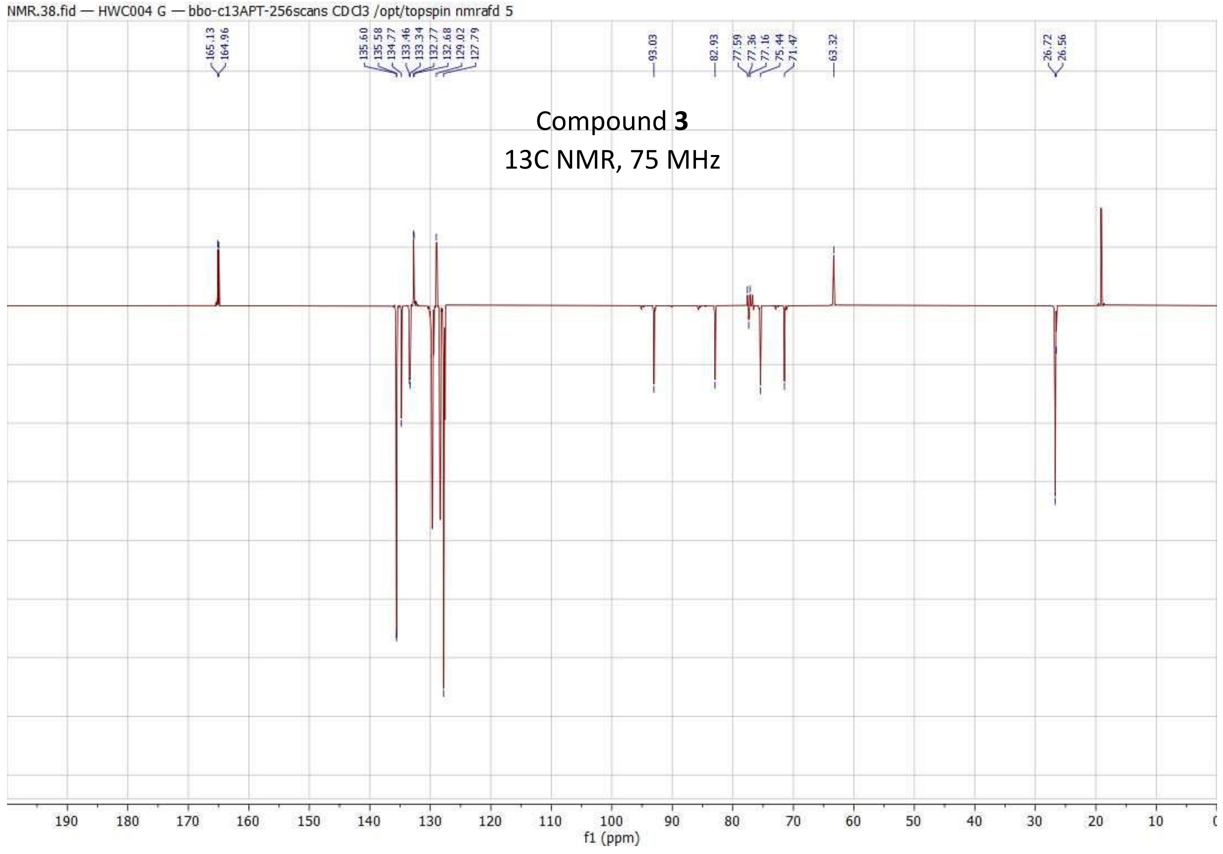
NMR.38.fid — HWC004 G — bbo-c13APT-256scans CDCl3 /opt/topspin nmrafd 5
Compound **3**
13C NMR, 75 MHz
f1 (ppm)

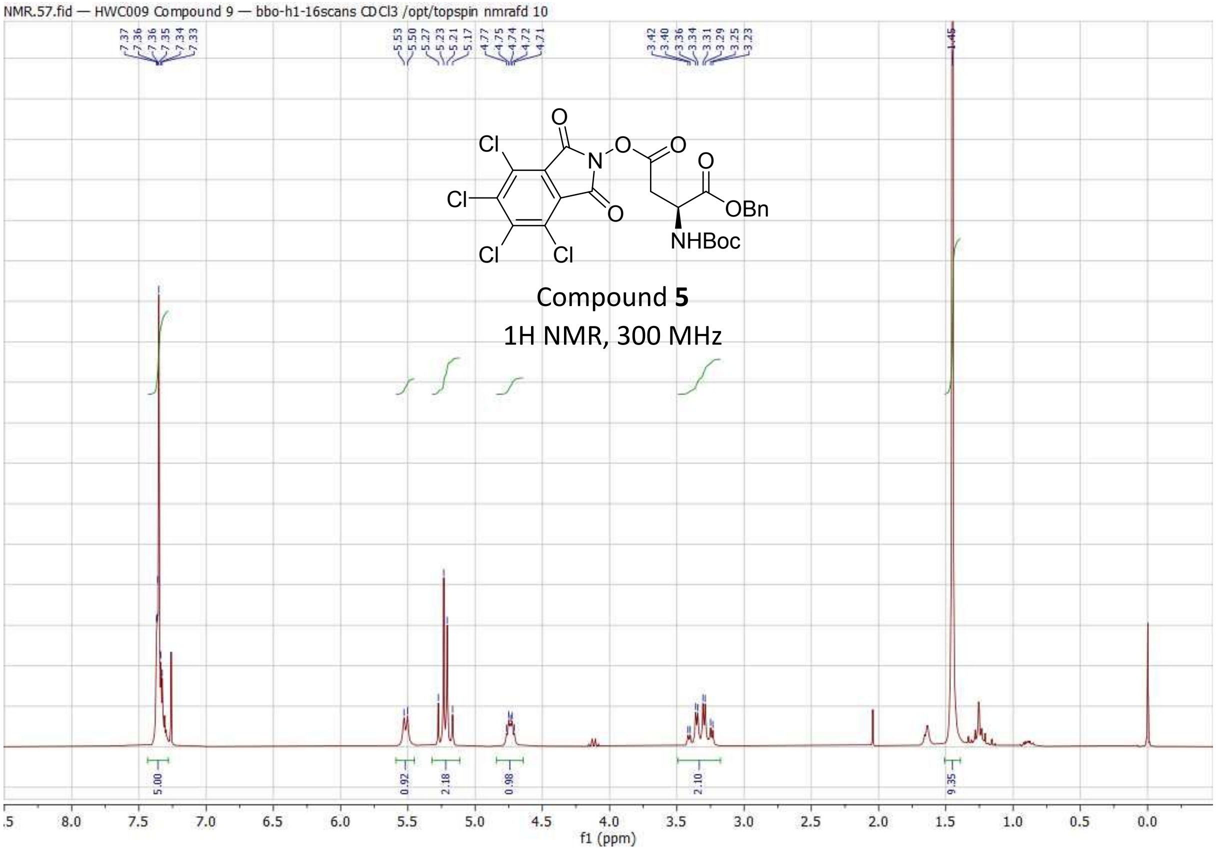
NMR.57.fid — HWC009 Compound 9 — bbo-h1-16scans CDCl3 /opt/topspin nmrafd 10
7.37 7.36 7.36 7.35 7.34 7.33
5.53 5.50 5.27 5.23 5.21 5.17 4.77 4.75 4.74 4.72 4.71
3.42 3.40 3.36 3.34 3.31 3.29 3.25 3.23
1.45
Cl
Cl
Cl
Cl
O
O
N
O
O
O
OBn
NHBoc
Compound **5**
1H NMR, 300 MHz
5.00
0.92
2.18
0.98
2.10
9.35
f1 (ppm)

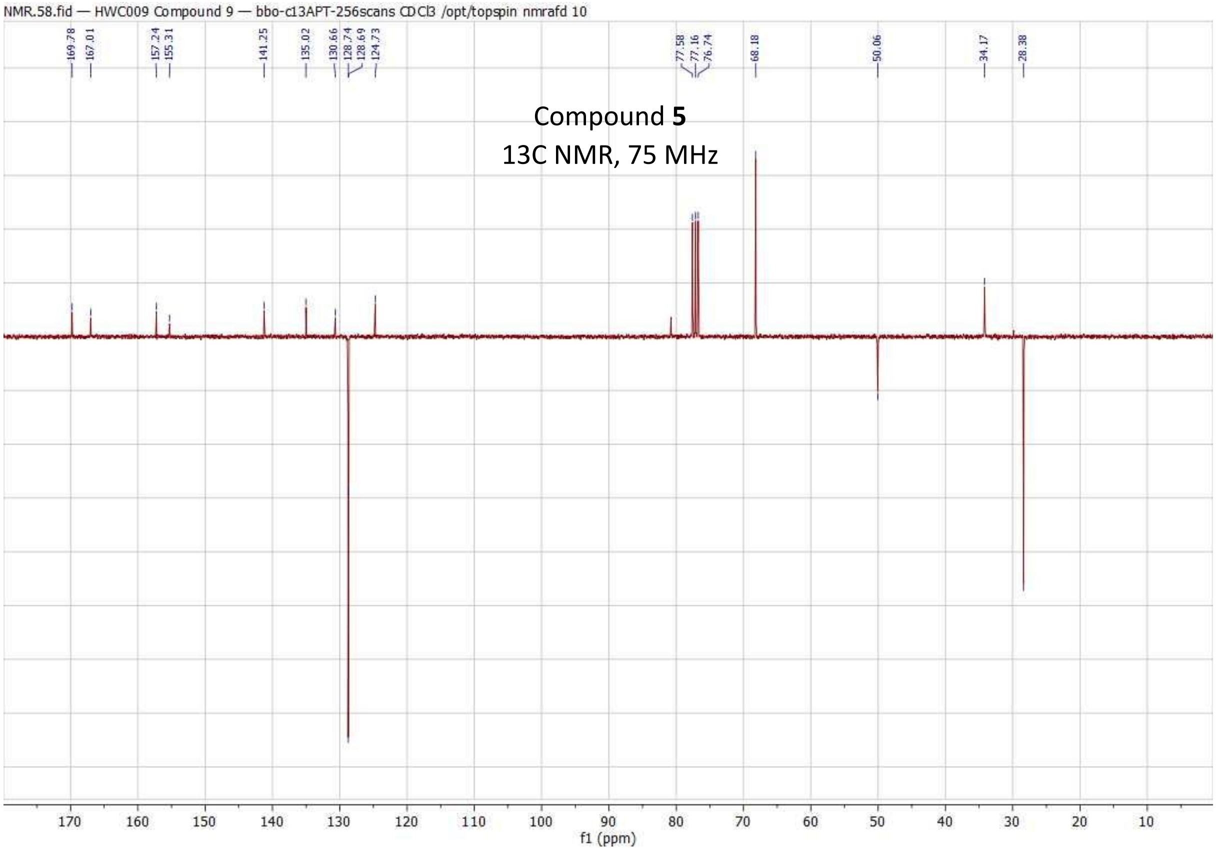
NMR.58.fid — HWC009 Compound 9 — bbo-c13APT-256scans CDCl3 /opt/topspin nmrafd 10
169.78 167.01 157.24 155.31 141.25 135.02 130.66 128.74 128.69 124.73
77.58 77.16 76.74 68.18 50.06 34.17 28.38
Compound **5**
13C NMR, 75 MHz
f1 (ppm)

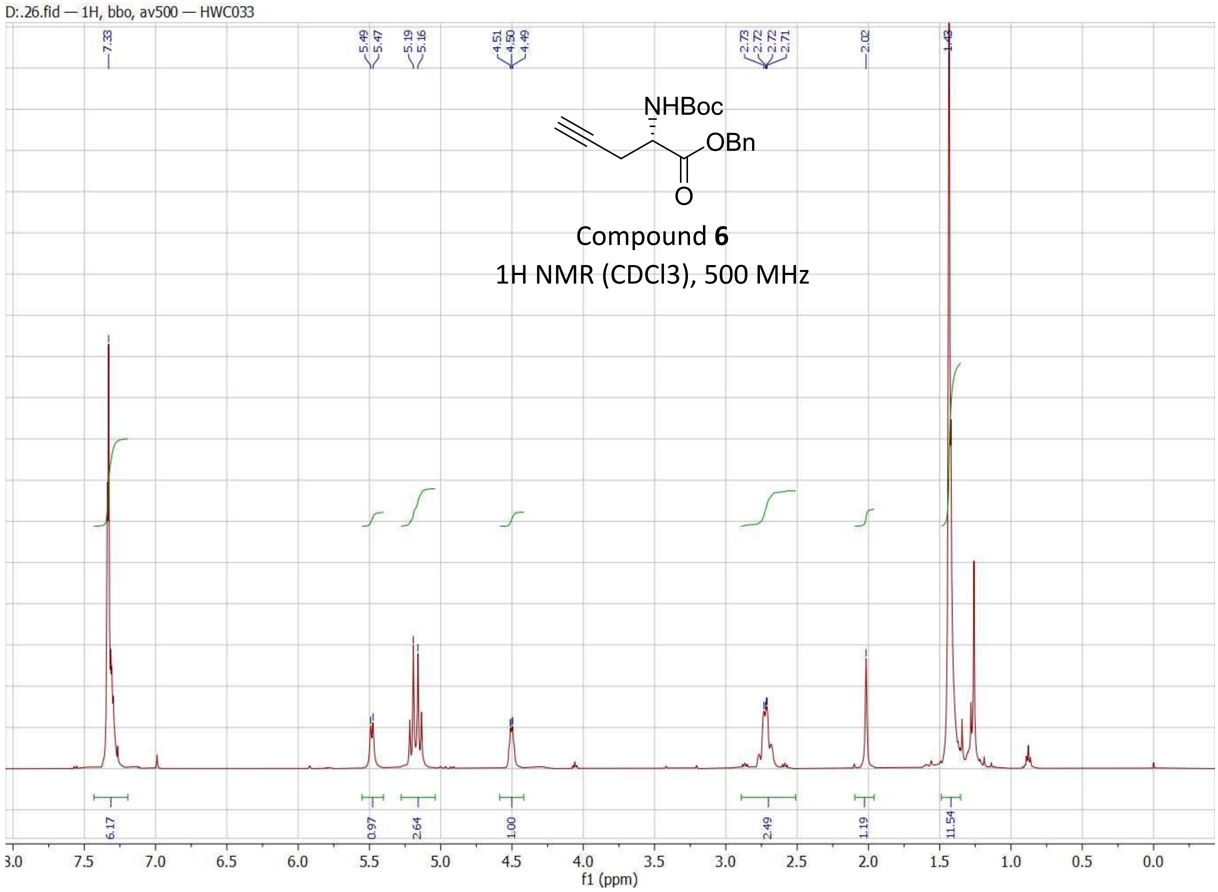
D:.26.fid — 1H, bbo, av500 — HWC033
NHBoc
OBn
O
Compound **6**
1H NMR (CDCl3), 500 MHz
f1 (ppm)

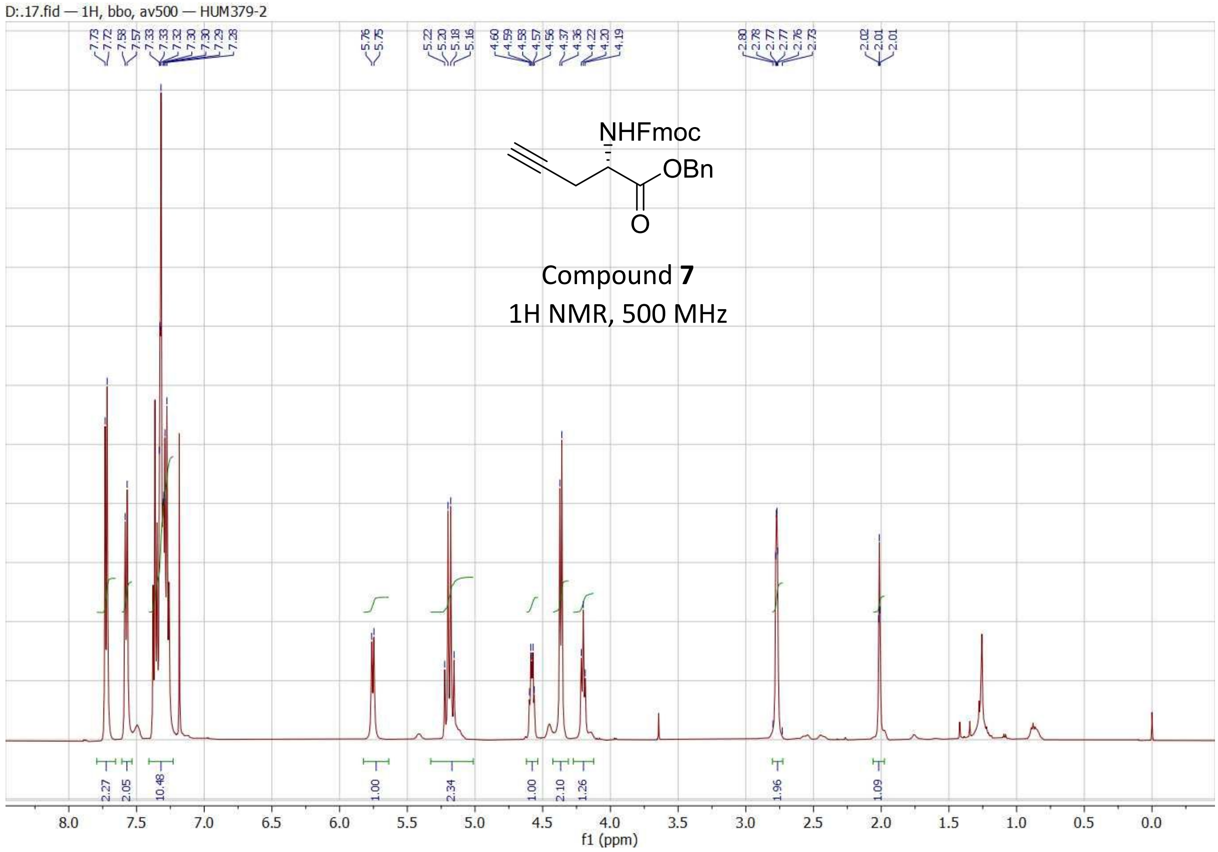
D:.17.fid — 1H, bbo, av500 — HUM379-2
NHFmoc
OBn
O
Compound **7**
1H NMR, 500 MHz
f1 (ppm)

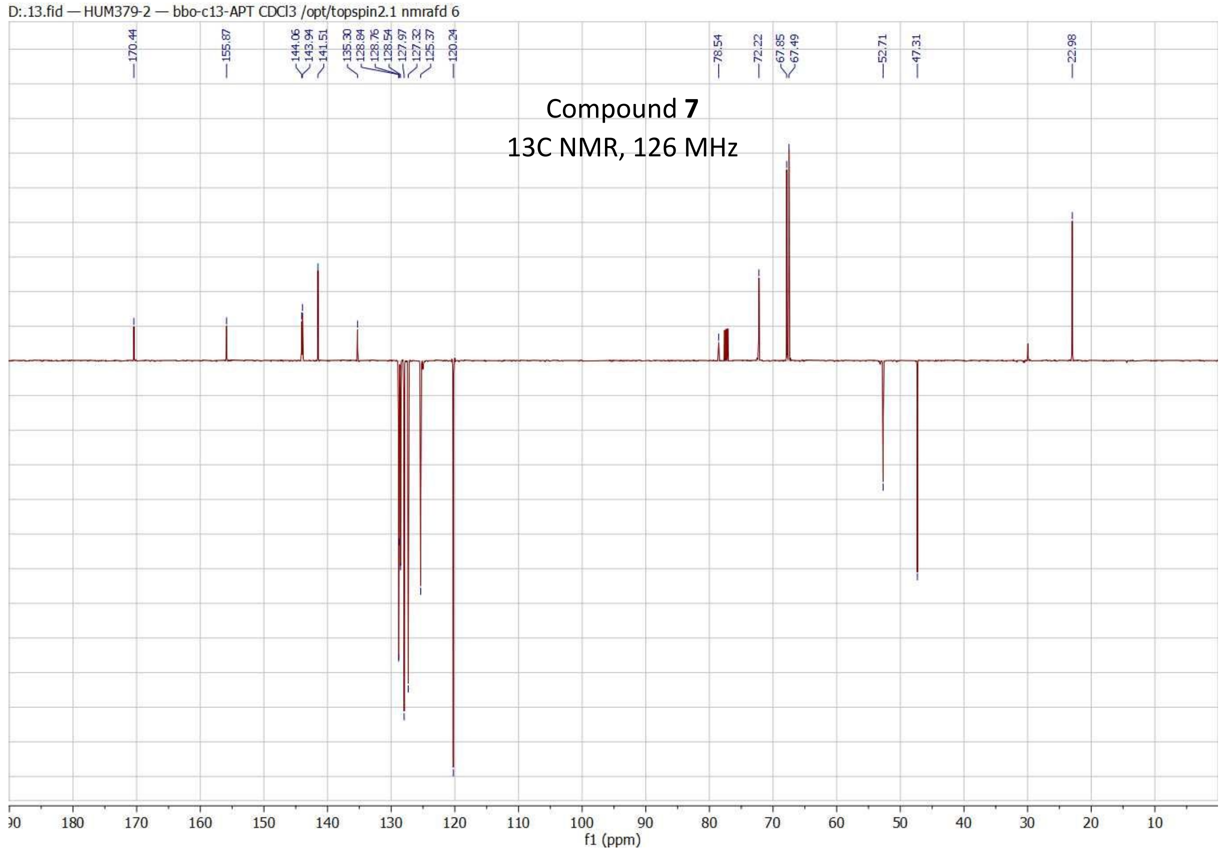
D:.13.fid — HUM379-2 — bbo-c13-APT CDCl3 /opt/topspin2.1 nmrafd 6
170.44
155.87
144.06
143.94
141.51
135.30
128.84
128.76
128.54
127.97
127.32
125.37
120.24
78.54
72.22
67.85
67.49
52.71
47.31
22.98
Compound **7**
13C NMR, 126 MHz
f1 (ppm)

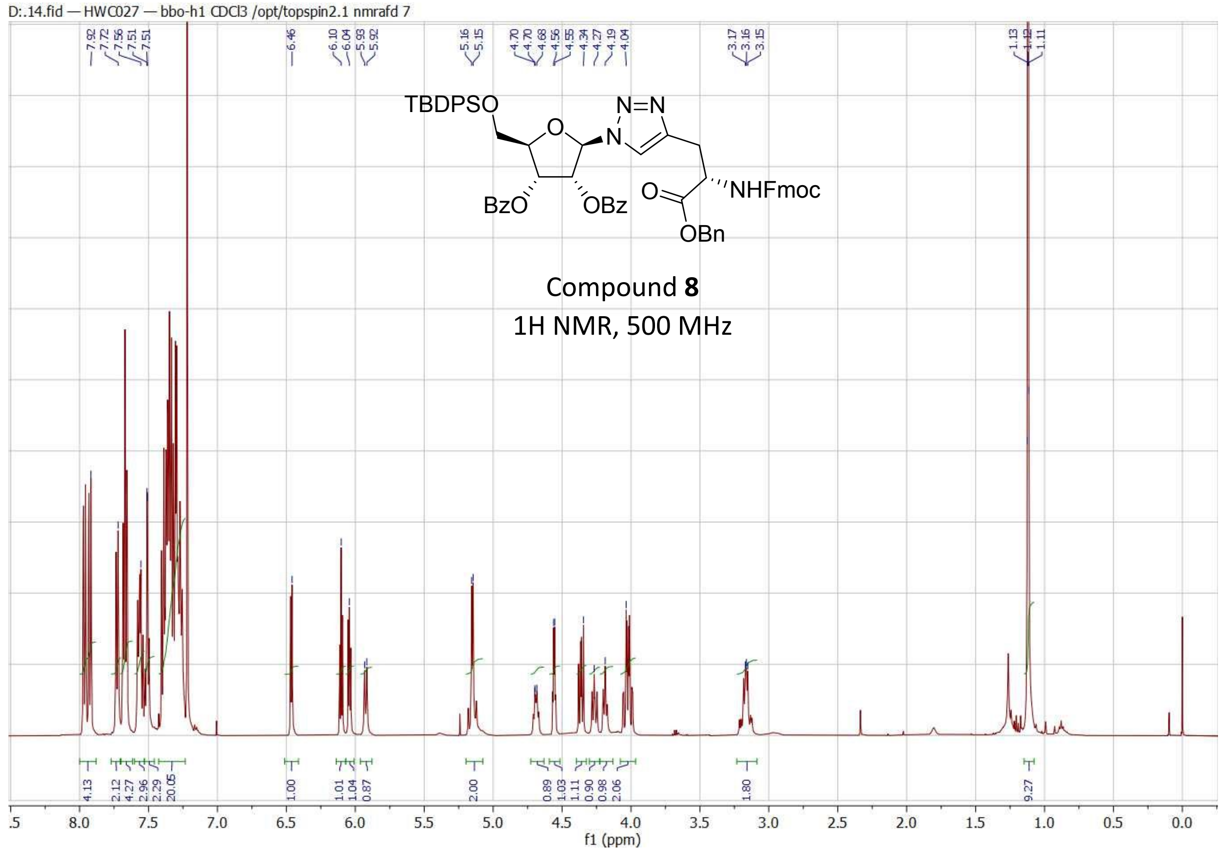
D:.14.fid — HWC027 — bbo-h1 CDCl3 /opt/topspin2.1 nmrafd 7
TBDPSO
O
N=N
N
BzO
OBz
O
NHFmoc
OBn
Compound **8**
1H NMR, 500 MHz
f1 (ppm)

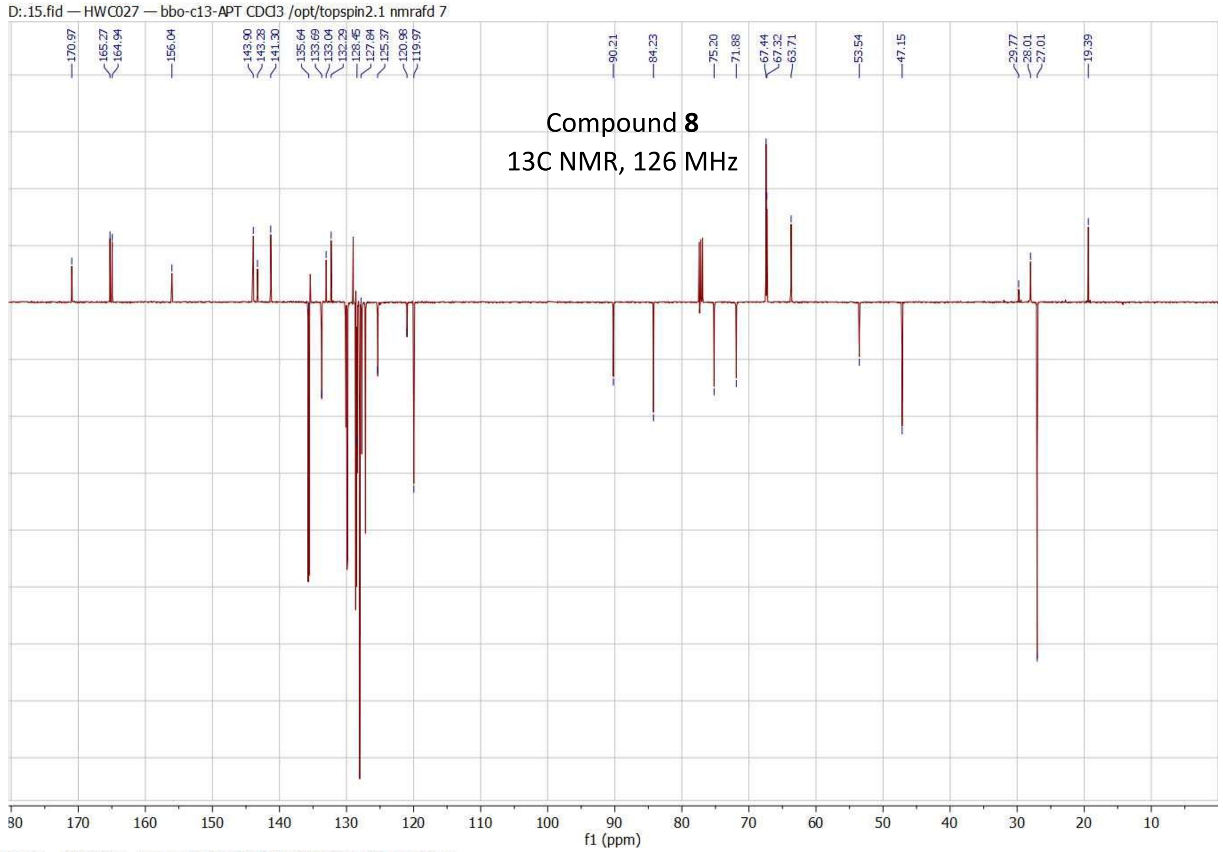

D:.15.fid — HWC027 — bbo-c13-APT CDCl3 /opt/topspin2.1 nmrafd 7
170.97
165.27
164.94
156.04
143.90
143.28
141.30
135.64
133.69
133.04
132.29
128.45
127.84
125.37
120.98
119.97
90.21
84.23
75.20
71.88
67.44
67.32
63.71
53.54
47.15
29.77
28.01
27.01
19.39
Compound **8**
13C NMR, 126 MHz
f1 (ppm)

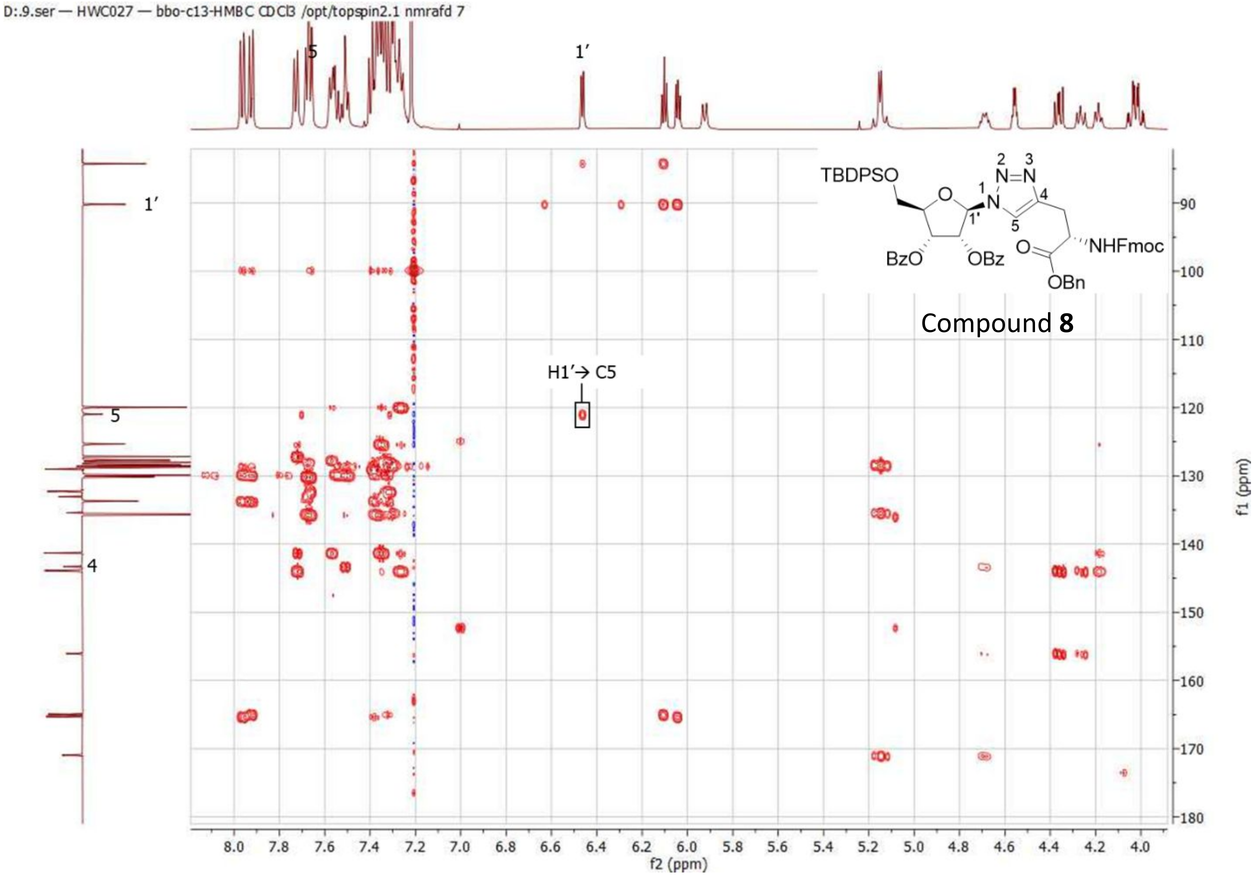

D:.9.ser — HWC027 — bbo-c13-HMBC CDCl3 /opt/topspin2.1 nmrafd 7
5
1′
1′
5
4
H1′→ C5
TBDPSO
BzO
OBz
NHFmoc
OBn
Compound **8**
f2 (ppm)
f1 (ppm)

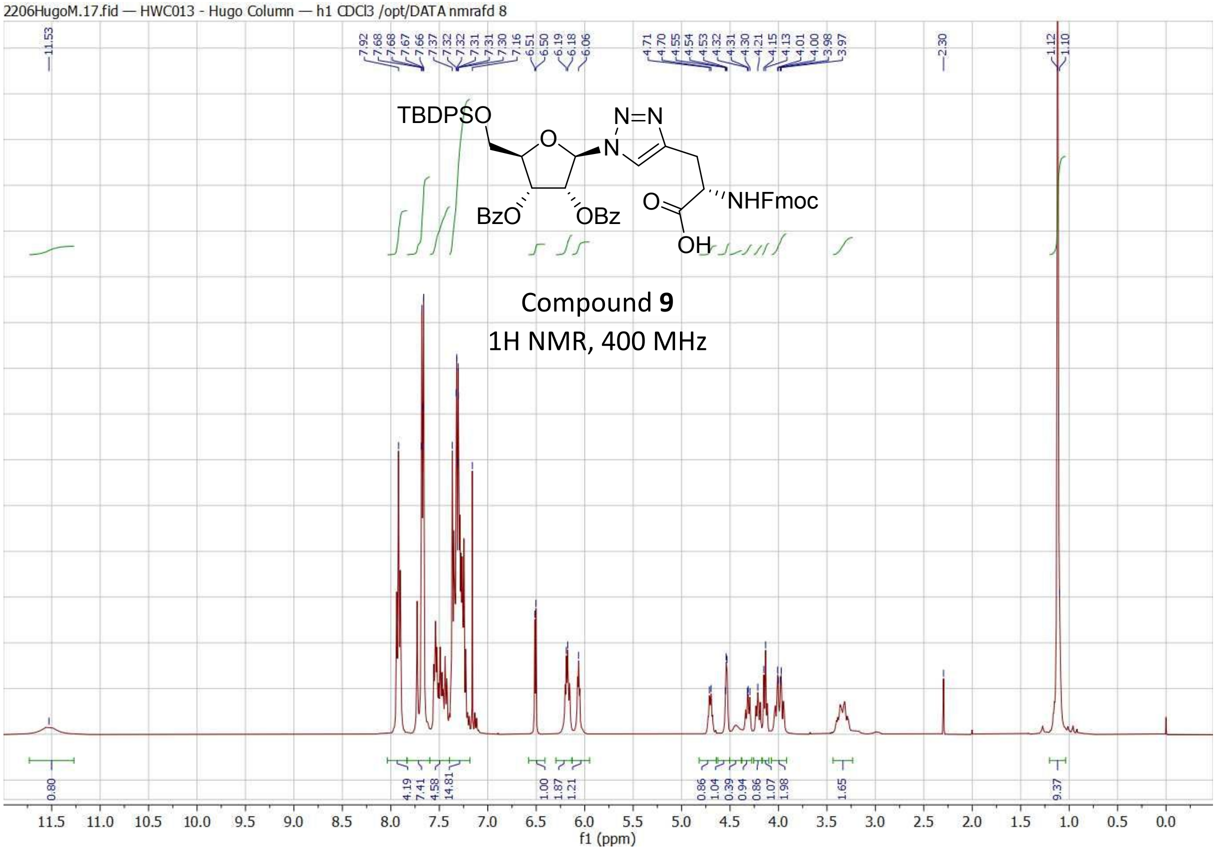
2206HugoM.17.fid — HWC013 - Hugo Column — h1 CDCl3 /opt/DATA nmrafd 8
TBDPSO
BzO
OBz
NHFmoc
OH
Compound **9**
1H NMR, 400 MHz
f1 (ppm)

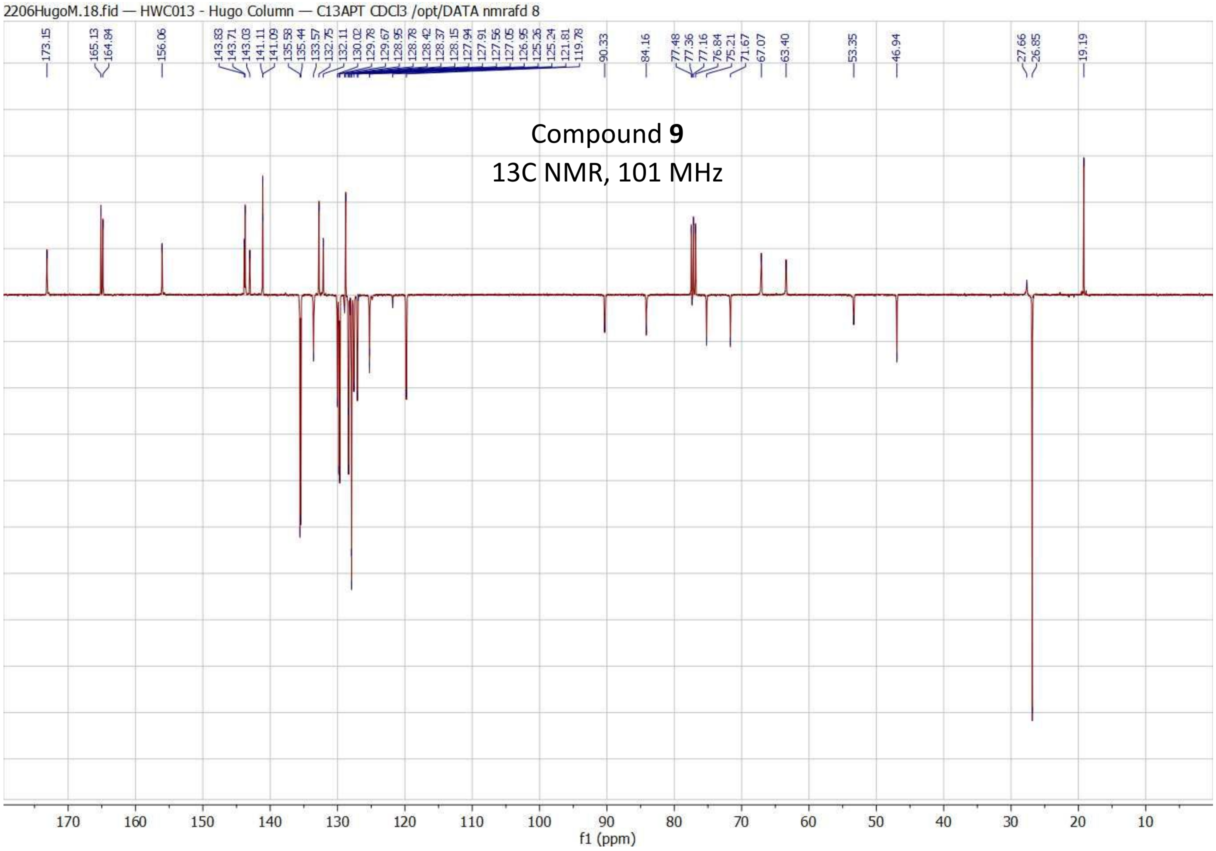
2206HugoM.18.fid — HWC013 - Hugo Column — C13APT CDCl3 /opt/DATA nmrafd 8
Compound **9**
13C NMR, 101 MHz
f1 (ppm)

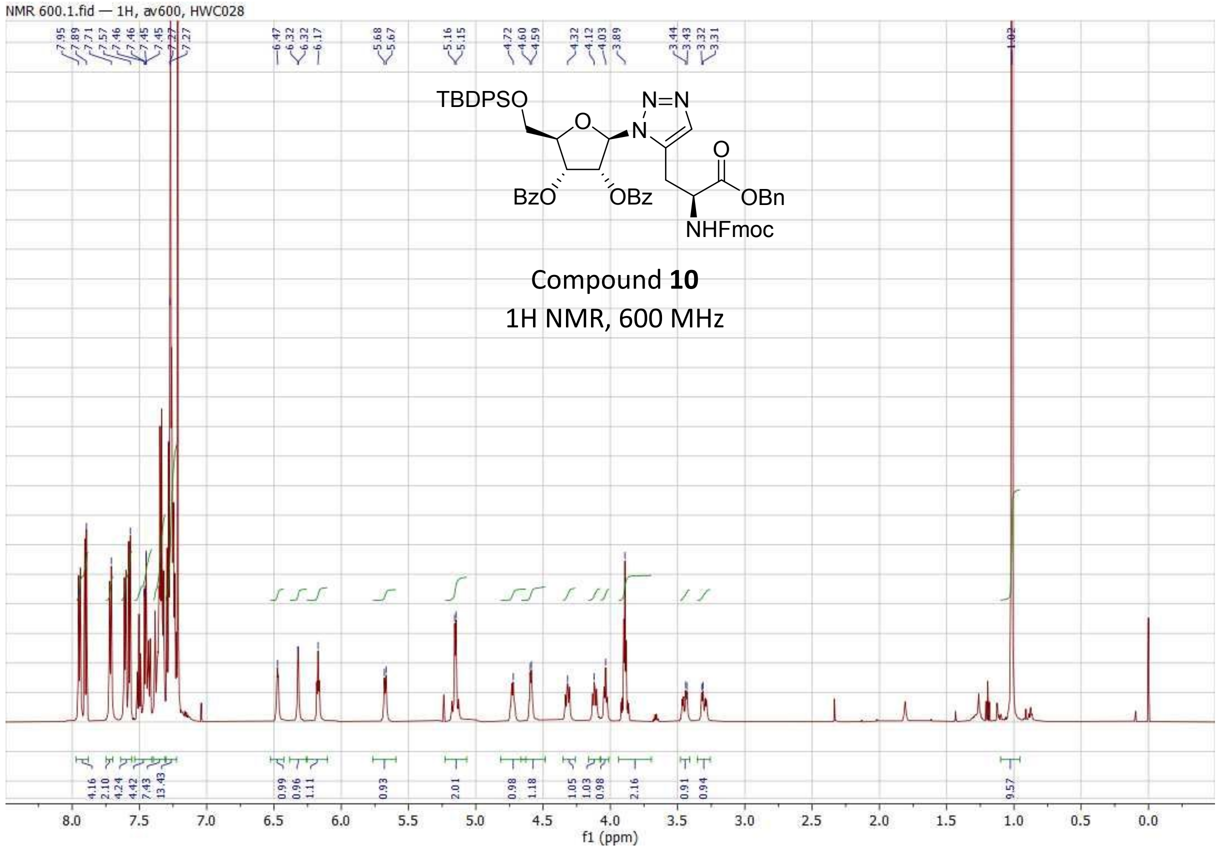
NMR 600.1.fid — 1H, av600, HWC028
7.95 7.89 7.71 7.57 7.46 7.46 7.45 7.45 7.27 7.27 6.47 6.32 6.32 6.17 5.68 5.67 5.16 5.15 4.72 4.60 4.59 4.32 4.12 4.03 3.89 3.44 3.43 3.32 3.31 1.02
TBDPSO
N=N
N
O
BzO
OBz
OBn
NHFmoc
Compound 10
1H NMR, 600 MHz
4.16 2.10 4.24 4.42 7.43 13.43 0.99 0.96 1.11 0.93 2.01 0.98 1.18 1.05 1.03 0.98 2.16 0.91 0.94 9.57
f1 (ppm)

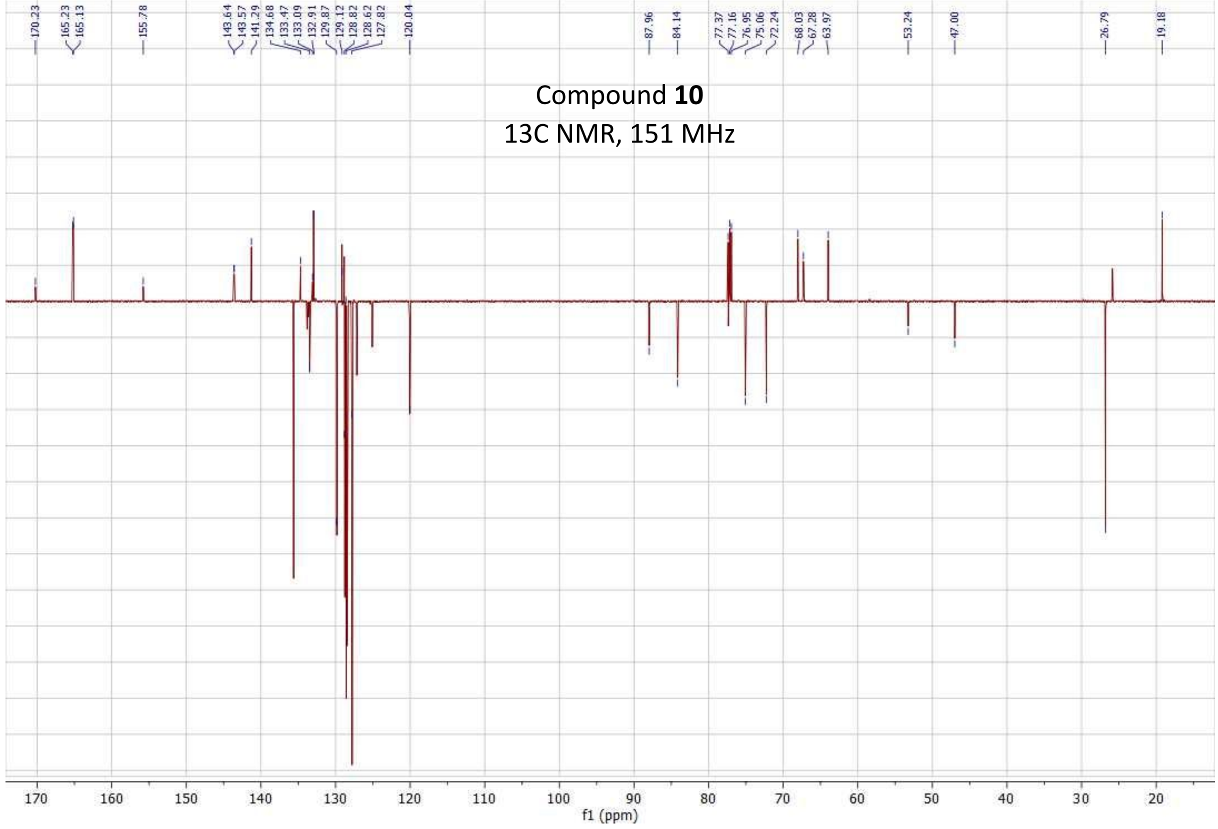
NMR 600.2.fid — HWC028, 13C-APT, av600,
170.23 165.23 165.13 155.78 143.64 143.57 141.29 134.68 133.47 133.09 132.91 129.87 129.12 128.82 128.62 127.82 120.04 87.96 84.14 77.37 77.16 76.95 75.06 72.24 68.03 67.28 63.97 53.24 47.00 26.79 19.18
Compound 10
13C NMR, 151 MHz
f1 (ppm)

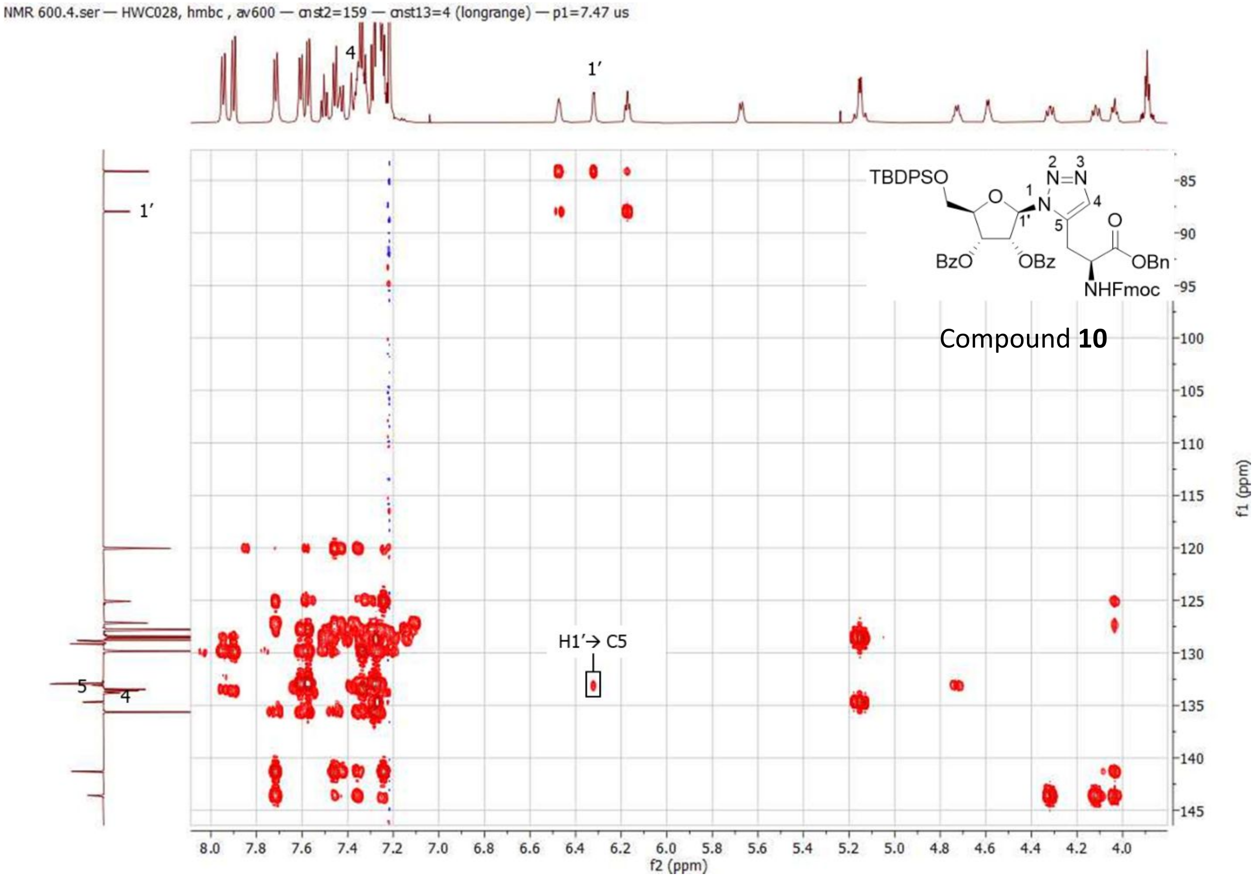
NMR 600.4.ser — HWC028, hmbc , av600 — cnst2=159 — cnst13=4 (longrange) — p1=7.47 us
TBDPSO
BzO
OBz
OBn
NHFmoc
Compound 10
H1'→ C5
f2 (ppm)
f1 (ppm)

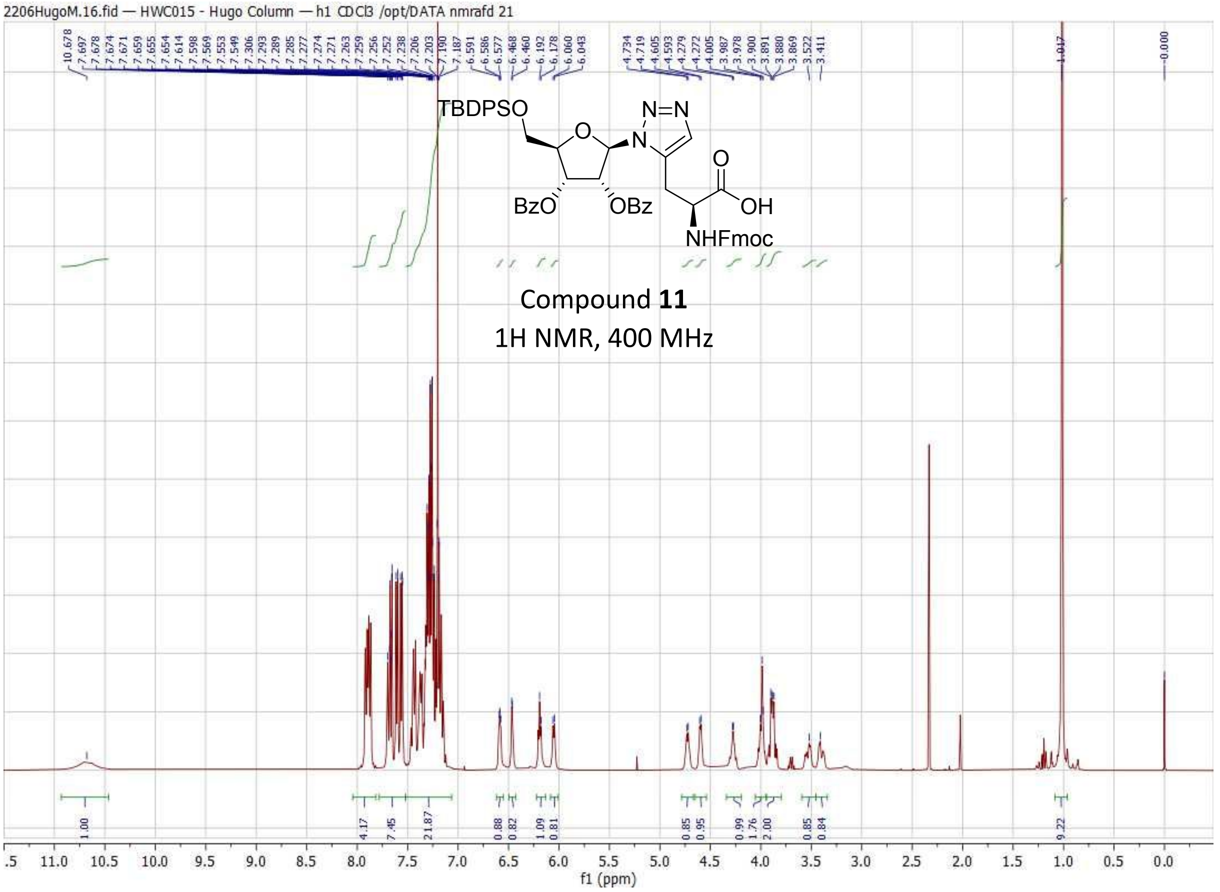
2206HugoM.16.fid — HWC015 - Hugo Column — h1 CDCl3 /opt/DATA nmrafd 21
TBDPSO
BzO
OBz
OH
NHFmoc
Compound 11
1H NMR, 400 MHz
f1 (ppm)

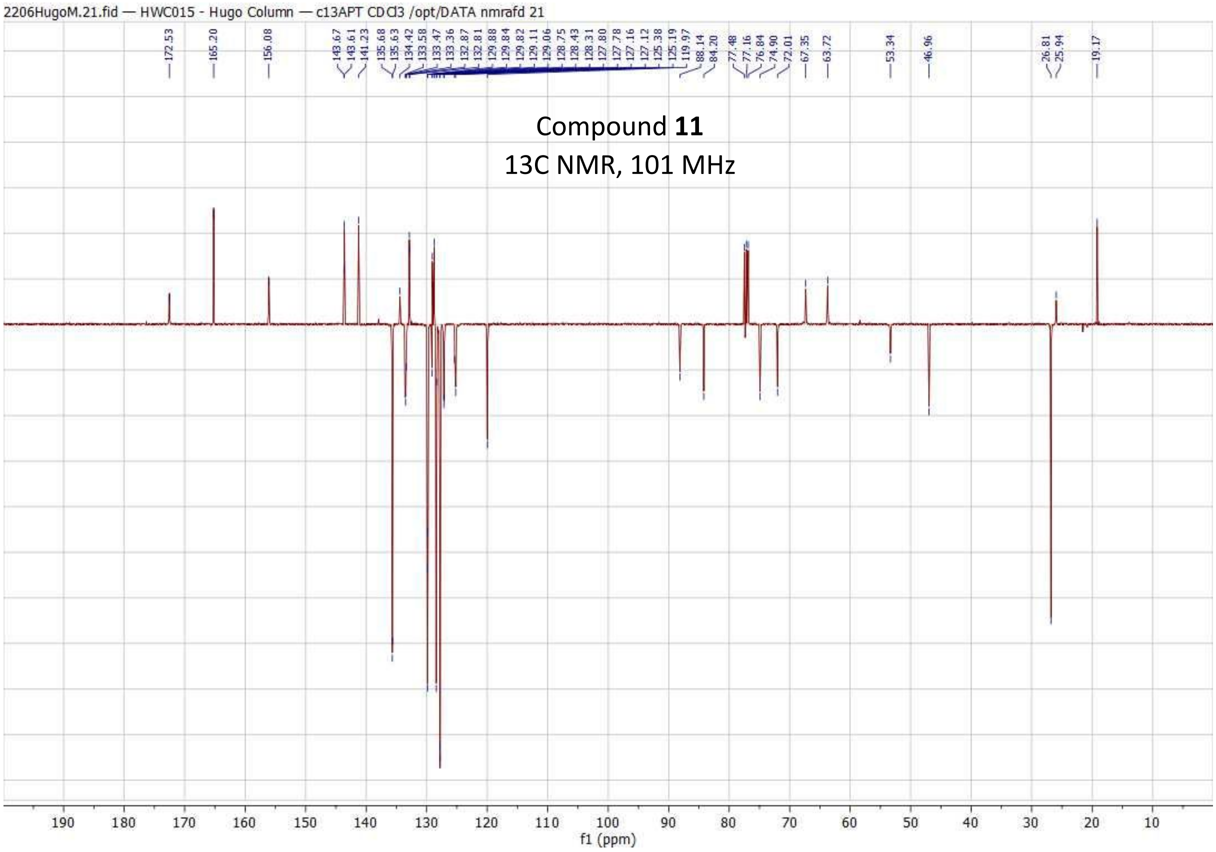
2206HugoM.21.fid — HWC015 - Hugo Column — c13APT CDCl3 /opt/DATA nmrafd 21
172.53
165.20
156.08
143.67
143.61
141.23
135.68
135.63
134.42
133.58
133.47
133.36
132.87
132.81
129.88
129.84
129.82
129.11
129.06
128.75
128.43
128.31
127.80
127.78
127.16
127.12
125.38
125.19
119.97
88.14
84.20
77.48
77.16
76.84
74.90
72.01
67.35
63.72
53.34
46.96
26.81
25.94
19.17
Compound 11
13C NMR, 101 MHz
f1 (ppm)

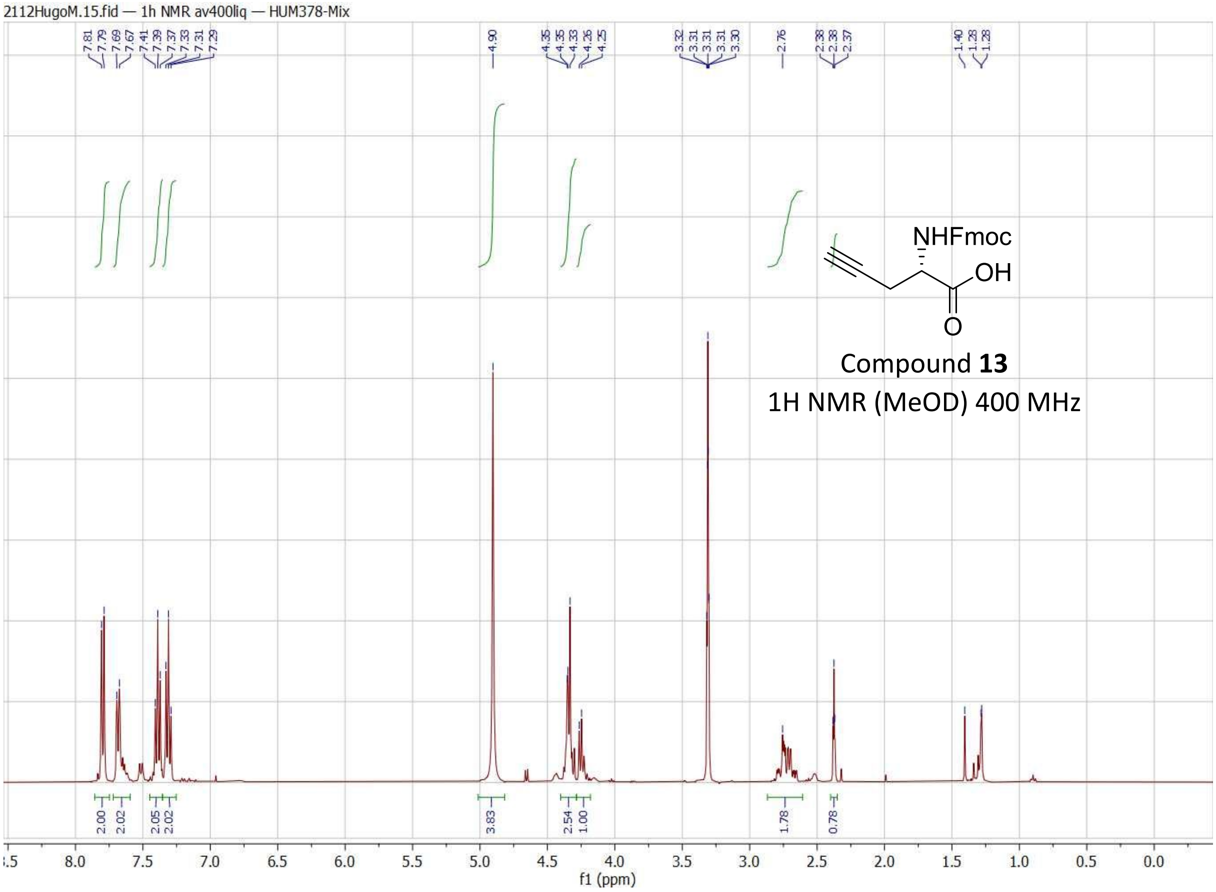
2112HugoM.15.fid — 1h NMR av400liq — HUM378-Mix
7.81
7.79
7.69
7.67
7.41
7.39
7.37
7.33
7.31
7.29
4.90
4.35
4.35
4.33
4.26
4.25
3.32
3.31
3.31
3.31
3.30
2.76
2.38
2.38
2.37
1.40
1.28
1.28
NHFmoc
OH
O
Compound 13
1H NMR (MeOD) 400 MHz
2.00
2.02
2.05
2.02
3.83
2.54
1.00
1.78
0.78
f1 (ppm)

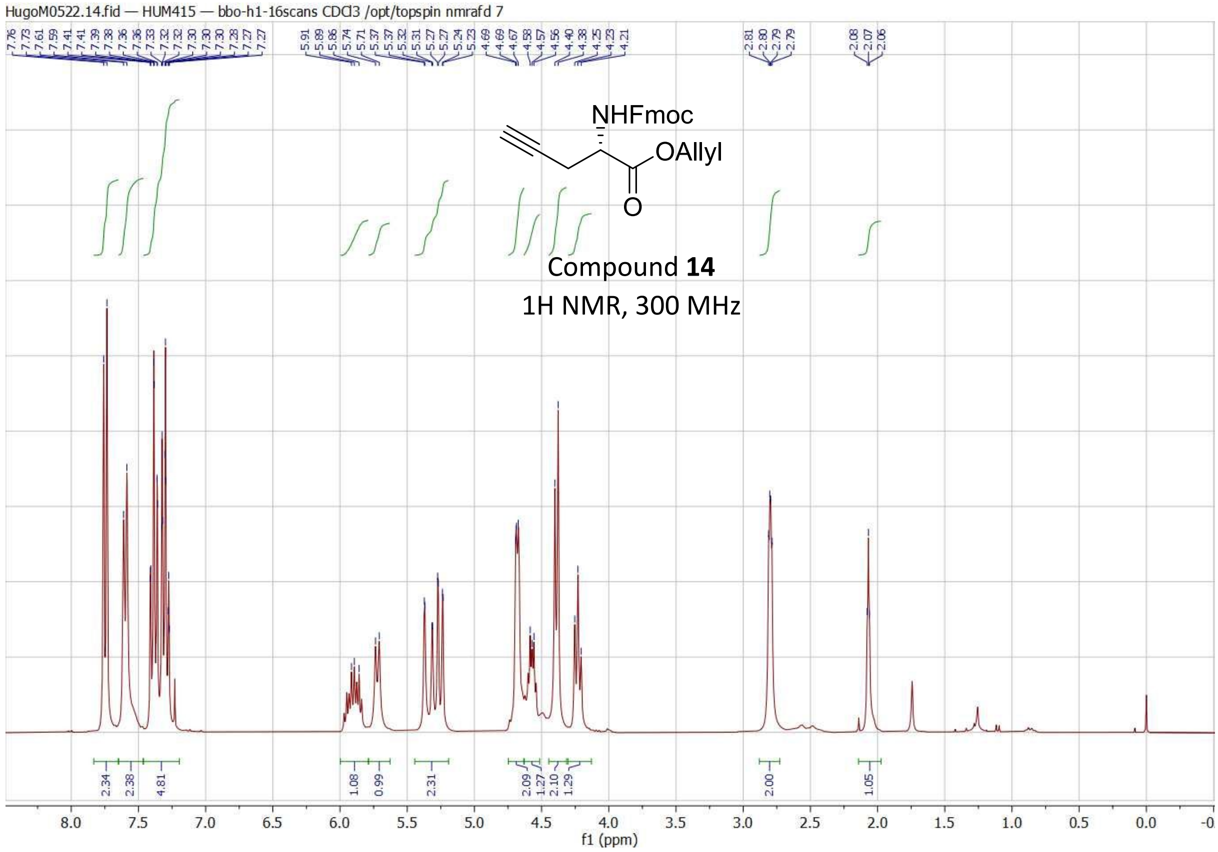
HugoM0522.14.fid — HUM415 — bbo-h1-16scans CDCl3 /opt/topspin nmrafd 7
7.76 7.73 7.61 7.59 7.41 7.41 7.39 7.38 7.36 7.36 7.33 7.32 7.32 7.30 7.30 7.28 7.27 7.27
5.91 5.89 5.86 5.74 5.71 5.37 5.37 5.32 5.31 5.27 5.27 5.24 5.23 4.69 4.67 4.58 4.57 4.56 4.40 4.38 4.25 4.23 4.21
2.81 2.80 2.79 2.79
2.08 2.07 2.06
NHFmoc
OAllyl
O
Compound **14**
1H NMR, 300 MHz
2.34 2.38 4.81 1.08 0.99 2.31 2.09 1.27 2.10 1.29 2.00 1.05
f1 (ppm)

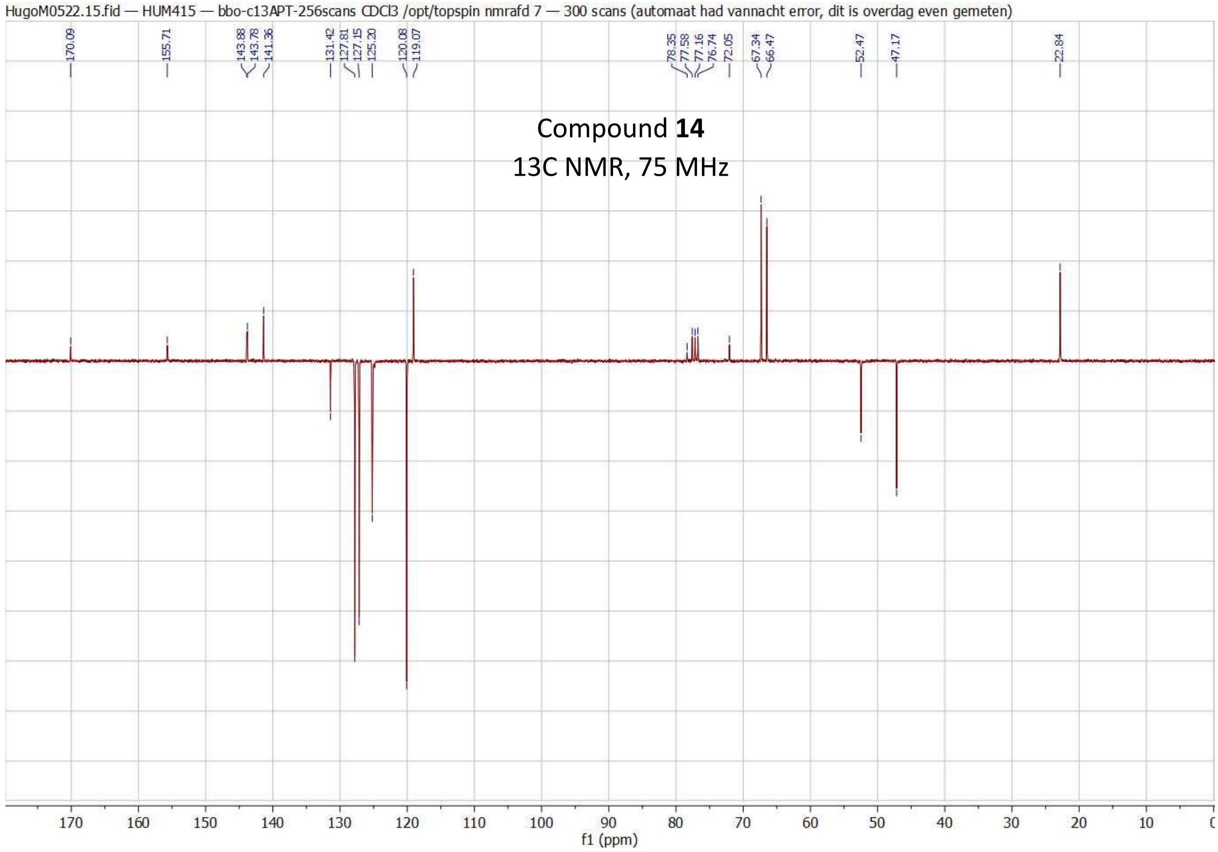
HugoM0522.15.fid — HUM415 — bbo-c13APT-256scans CDCl3 /opt/topspin nmrafd 7 — 300 scans (automaat had vannacht error, dit is overdag even gemeten)
170.09 155.71 143.88 143.78 141.36 131.42 127.81 127.15 125.20 120.08 119.07 78.35 77.58 77.16 76.74 72.05 67.34 66.47 52.47 47.17 22.84
Compound **14**
13C NMR, 75 MHz
f1 (ppm)

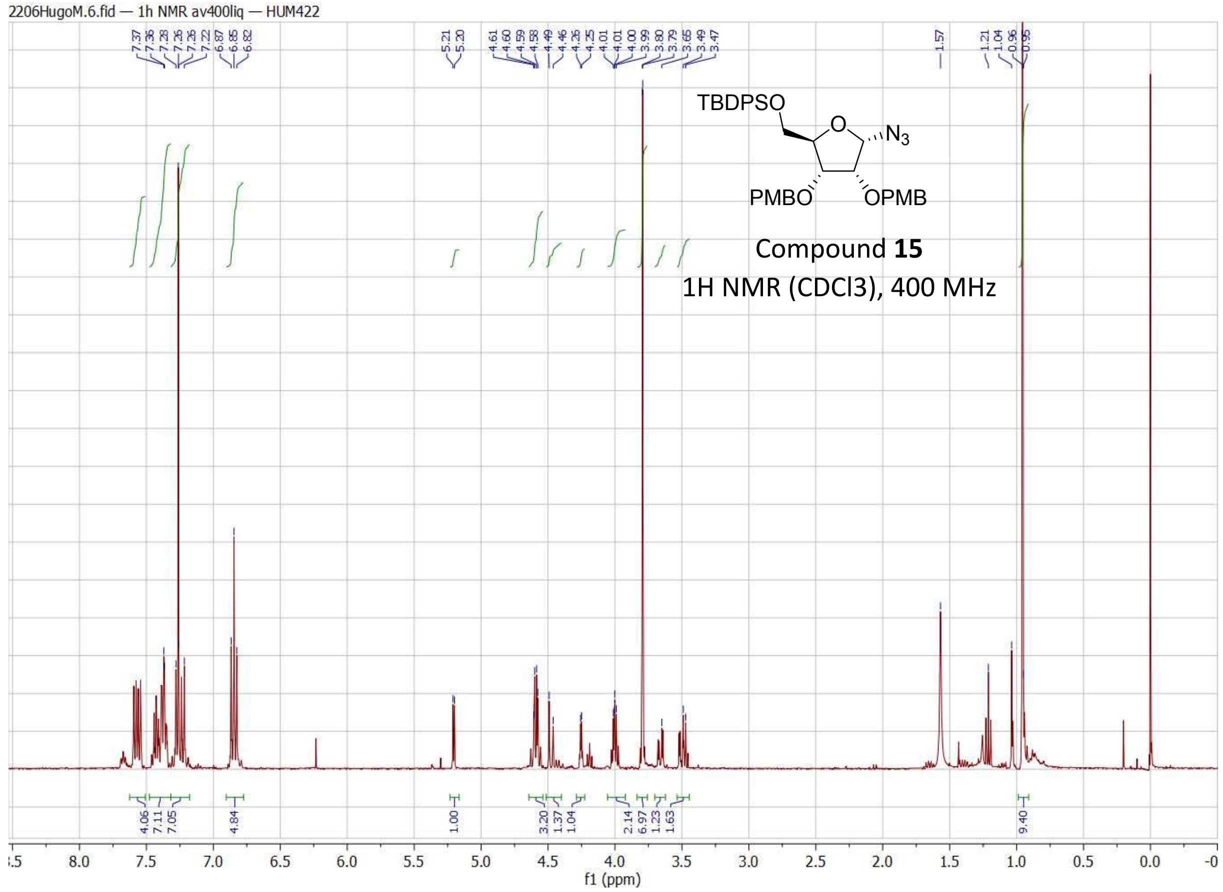
2206HugoM.6.fid — 1h NMR av400liq — HUM422
TBDPSO
O
N3
PMBO
OPMB
Compound 15
1H NMR (CDCl3), 400 MHz
f1 (ppm)

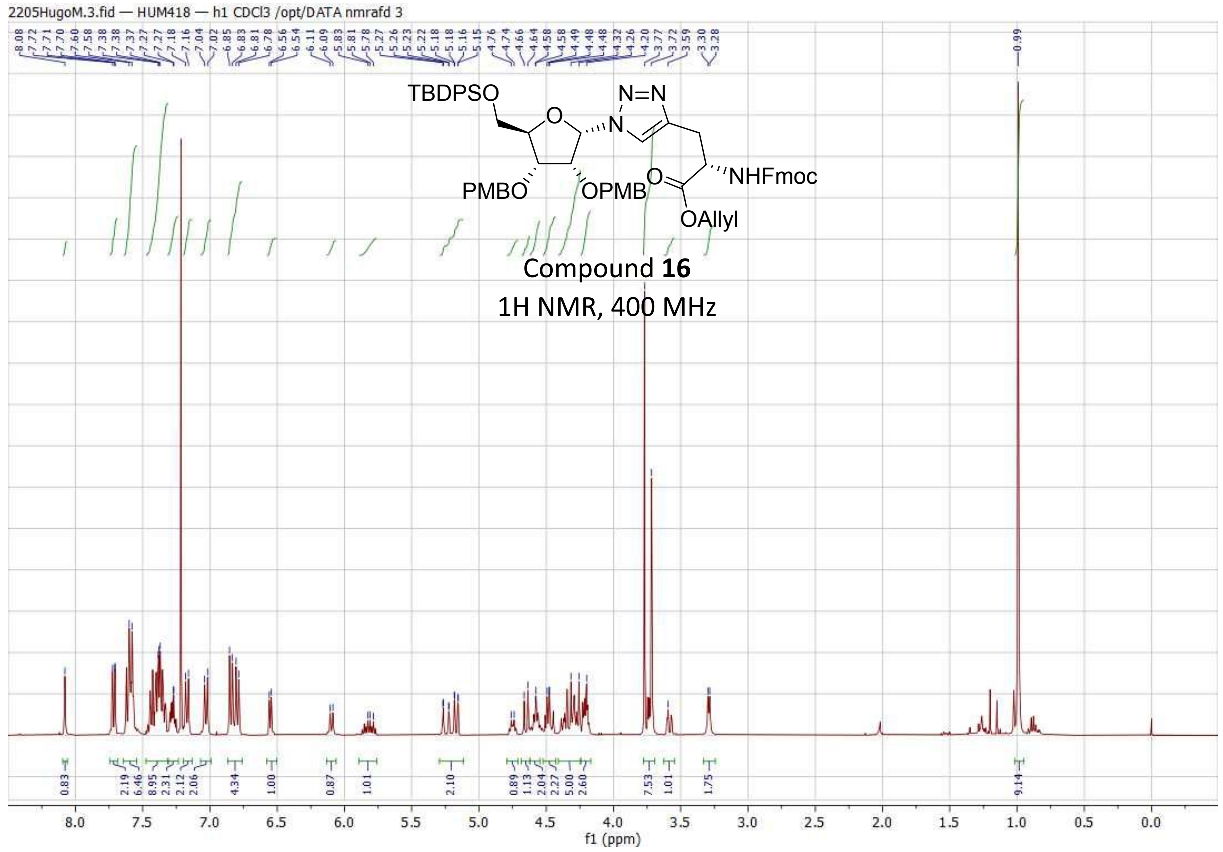
2205HugoM.3.fid — HUM418 — h1 CDCl3 /opt/DATA nmrafd 3
TBDPSO
O
N=N
N
NHFmoc
O
OAllyl
PMBO
OPMB
Compound 16
1H NMR, 400 MHz
f1 (ppm)

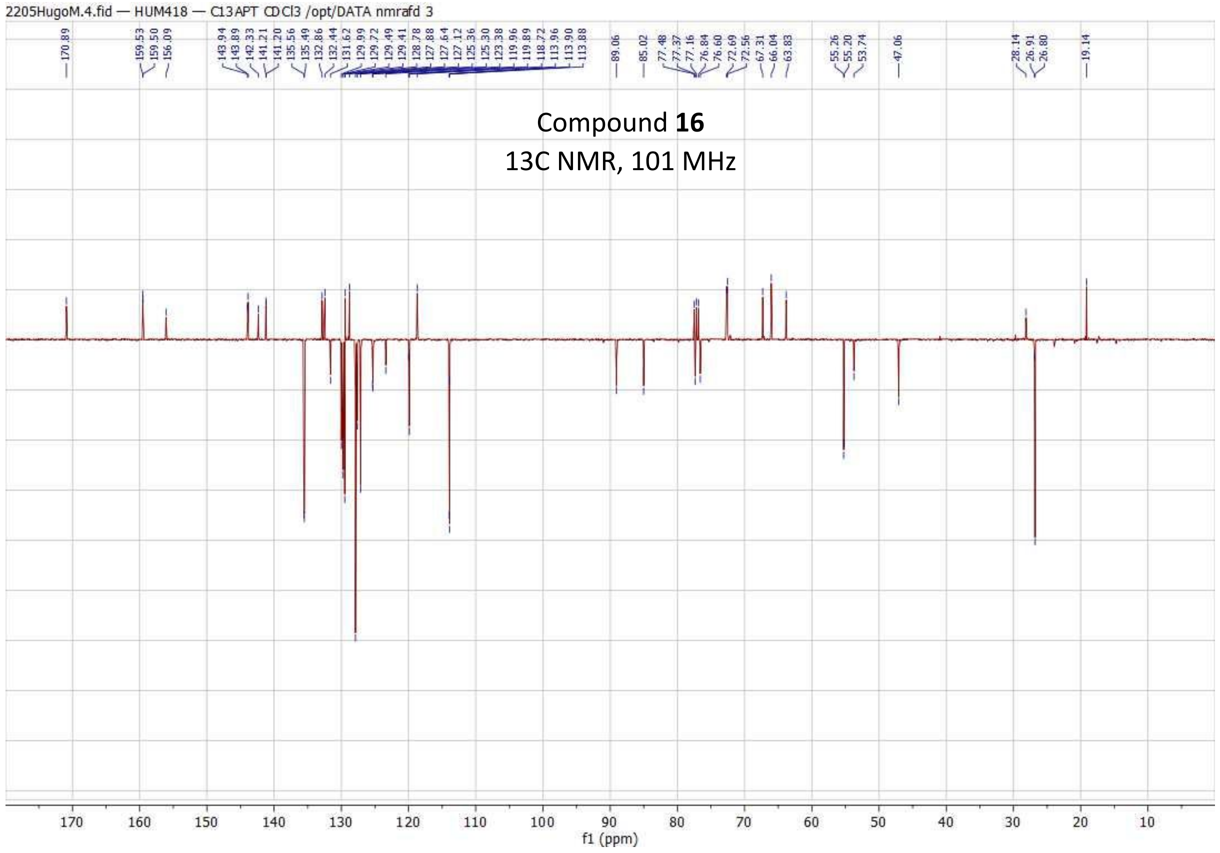
2205HugoM.4.fid — HUM418 — C13APT CDCl3 /opt/DATA nmrafd 3
170.89
159.53
159.50
156.09
143.94
143.89
142.33
141.21
141.20
135.56
135.49
132.86
132.44
131.62
129.99
129.72
129.49
129.41
128.78
127.88
127.64
127.12
125.36
125.30
123.38
119.96
119.89
118.72
113.96
113.90
113.88
89.06
85.02
77.48
77.37
77.16
76.84
76.60
72.69
72.56
67.31
66.04
63.83
55.26
55.20
53.74
47.06
28.14
26.91
26.80
19.14
Compound **16**
13C NMR, 101 MHz
f1 (ppm)

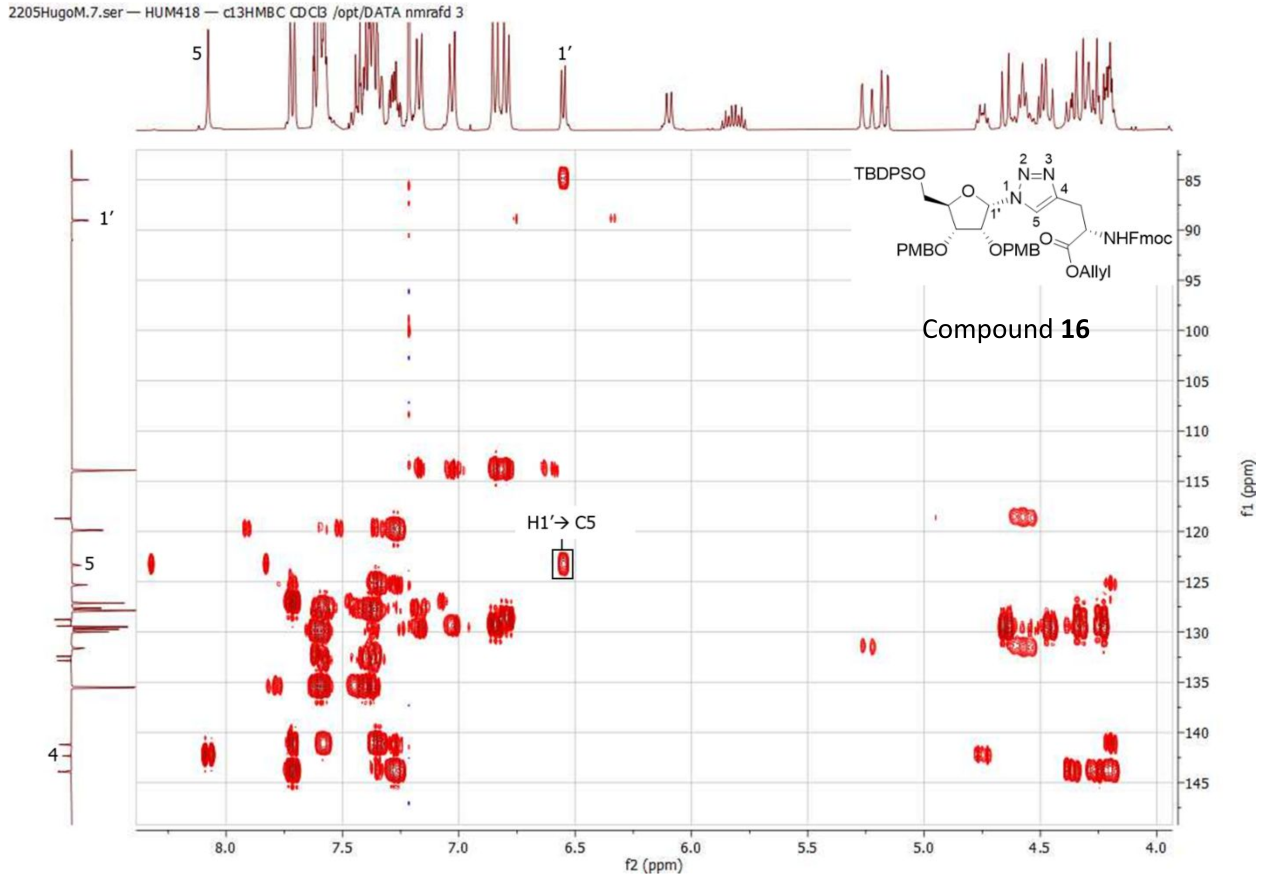
2205HugoM.7.ser — HUM418 — c13HMBC CDCl3 /opt/DATA nmrafd 3
5
1′
TBDPSO
PMBO
OPMB
NHFmoc
OAllyl
Compound **16**
H1′→ C5
4
f2 (ppm)
f1 (ppm)

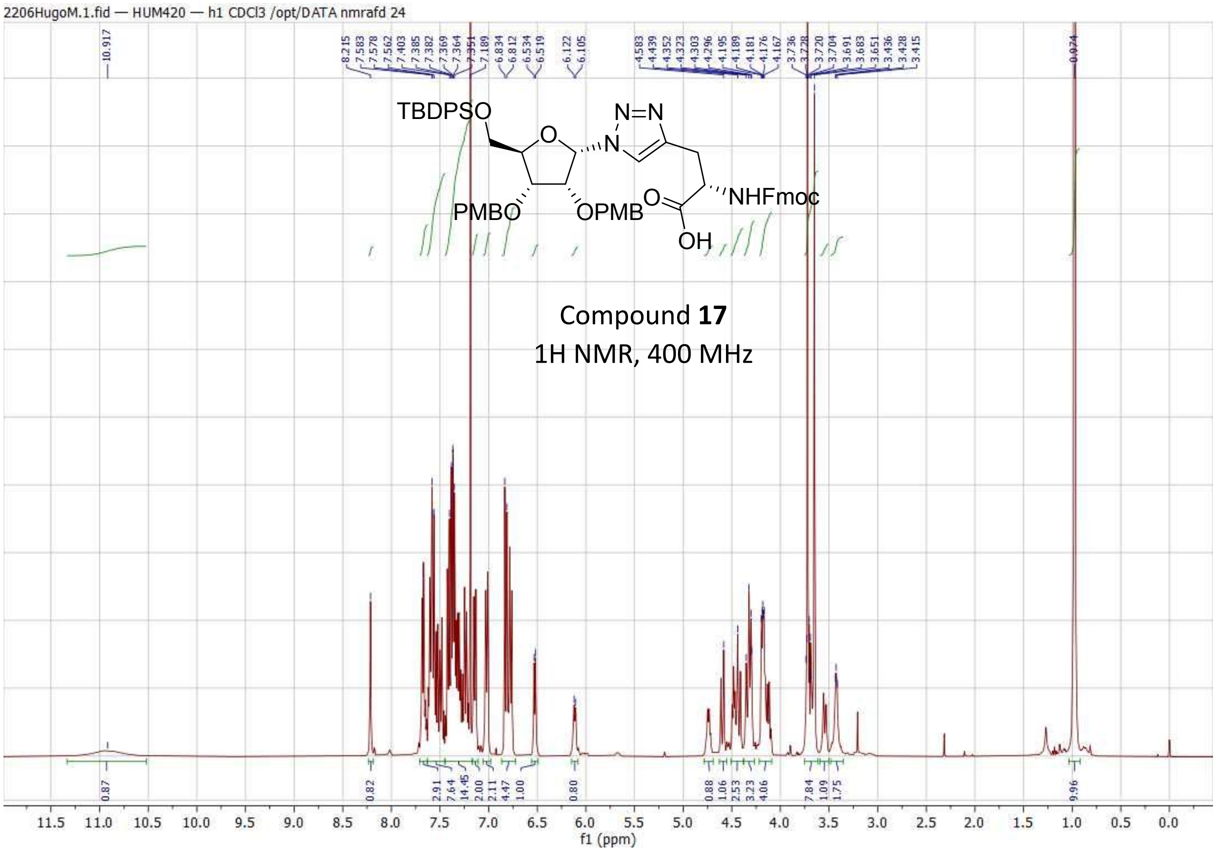
2206HugoM.1.fid — HUM420 — h1 CDCl3 /opt/DATA nmrafd 24
TBDPSO
N=N
N
O
PMBO
OPMB
O
OH
NHFmoc
Compound **17**
1H NMR, 400 MHz
f1 (ppm)

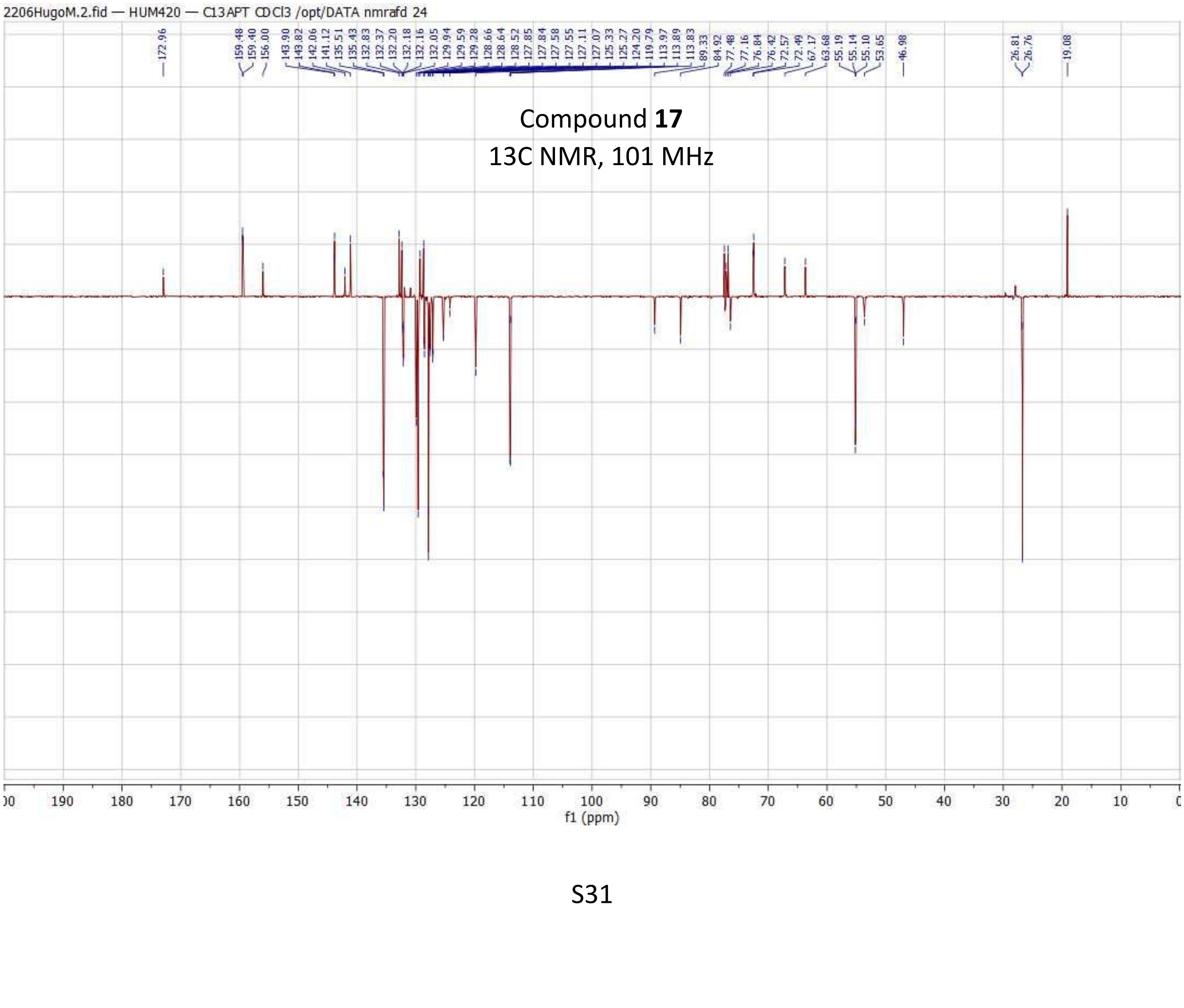
2206HugoM.2.fid — HUM420 — C13APT CDCl3 /opt/DATA nmrafd 24
Compound **17**
13C NMR, 101 MHz
f1 (ppm)

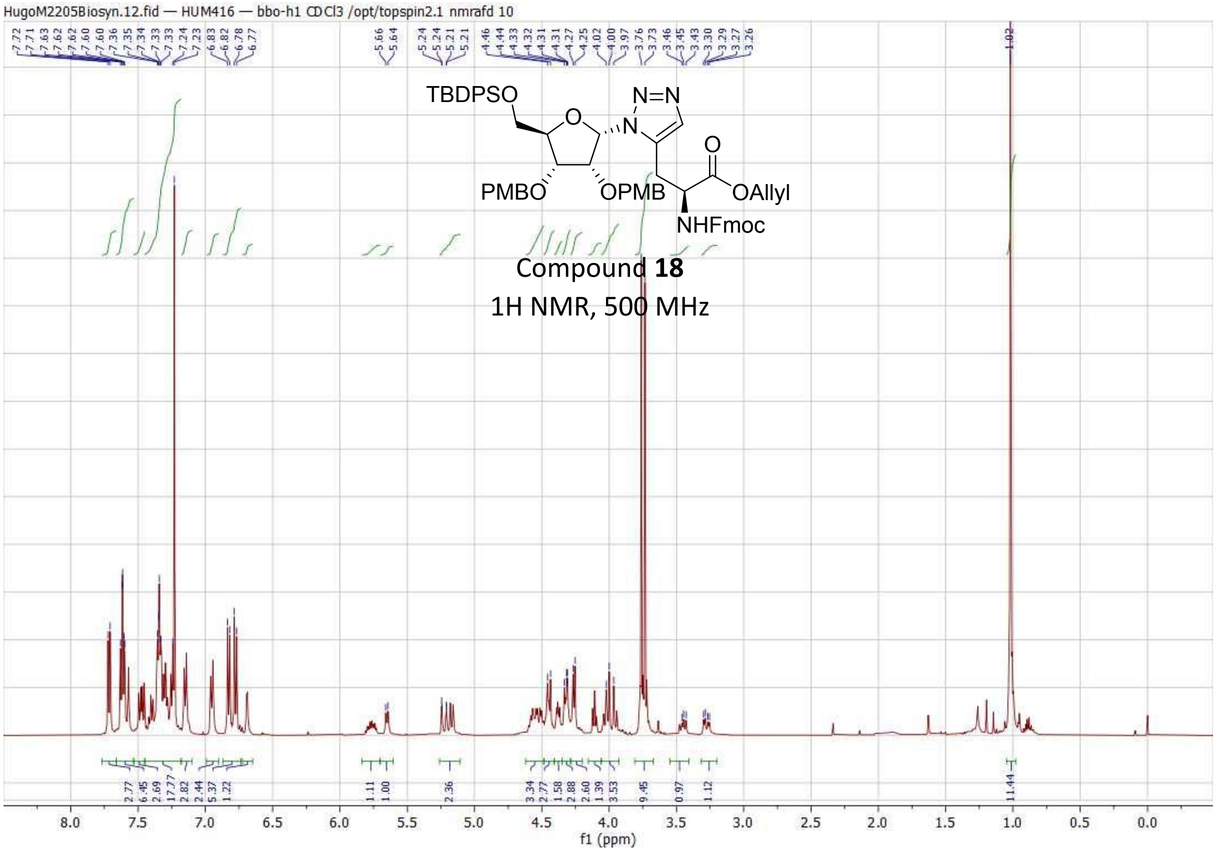

Compound **18**

1H NMR, 500 MHz

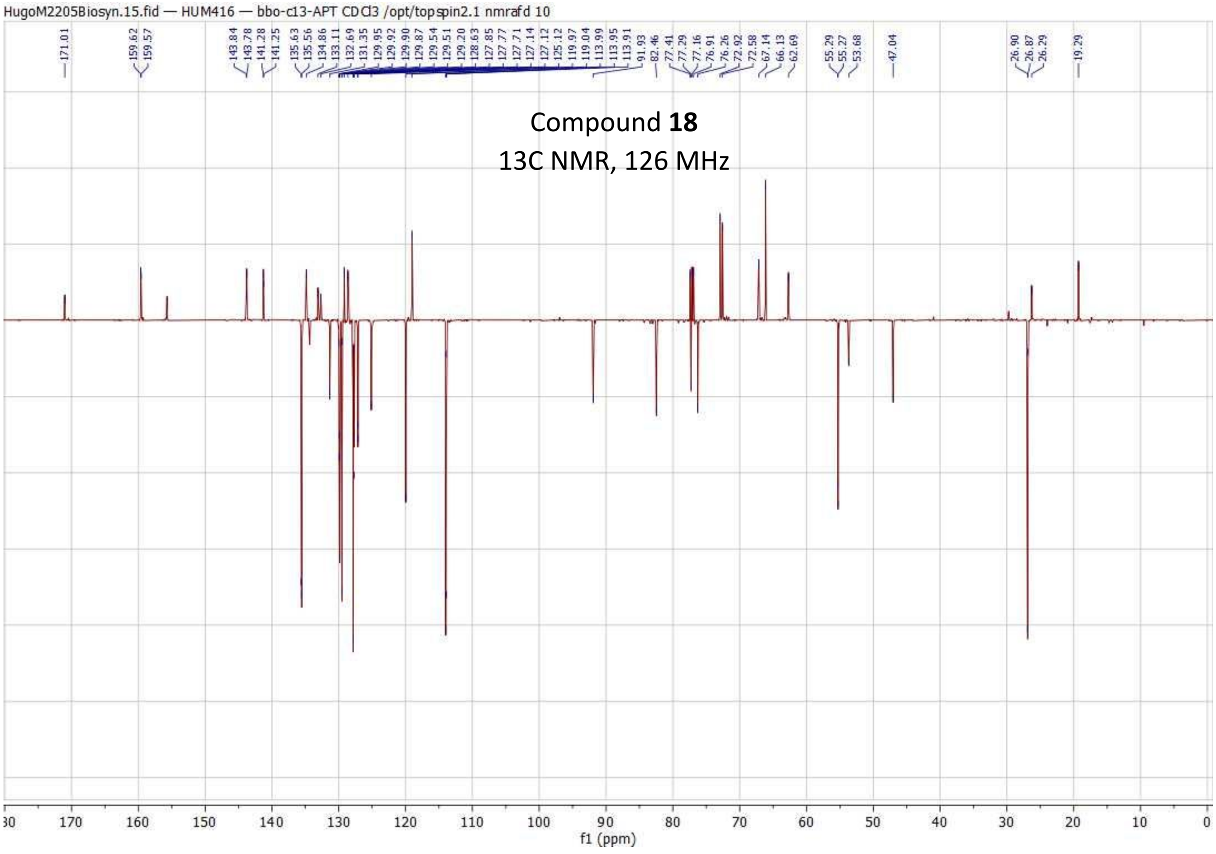

Compound **18**

13C NMR, 126 MHz

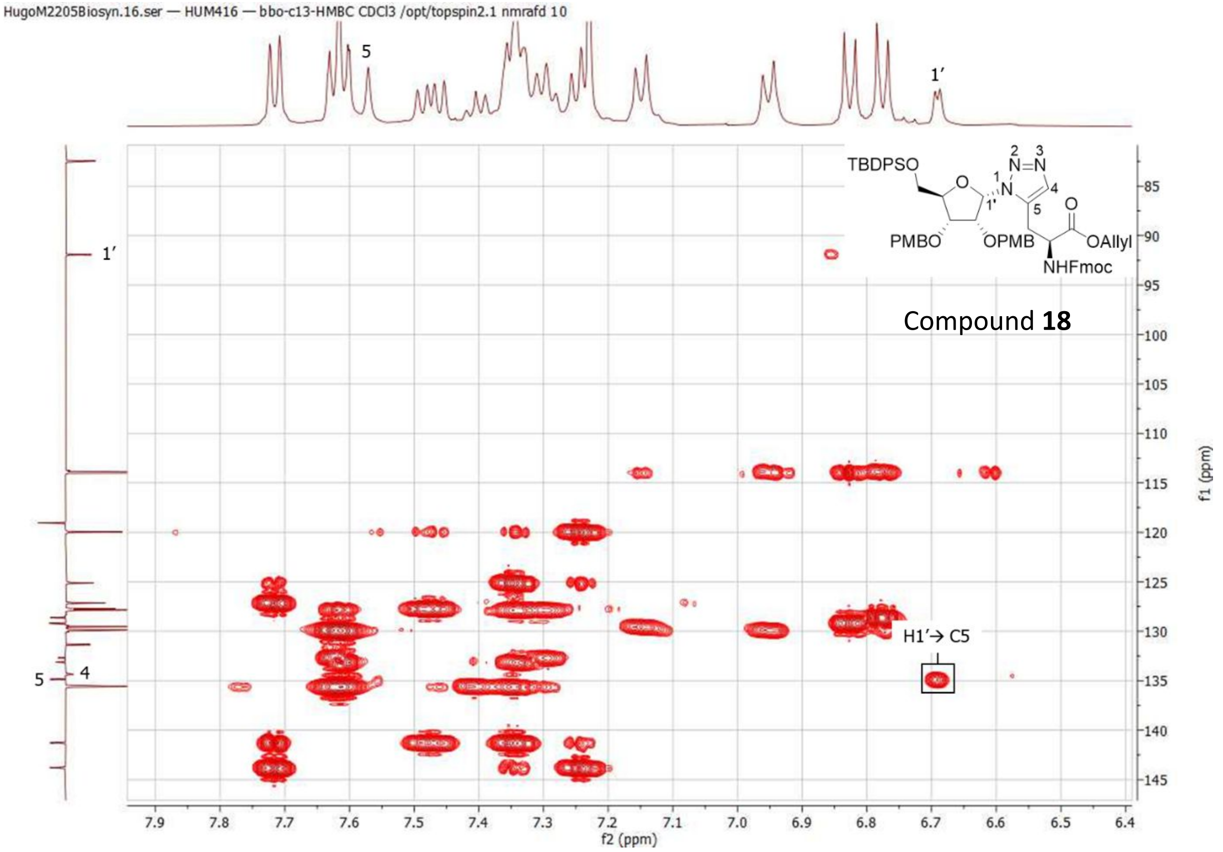

Compound **18**

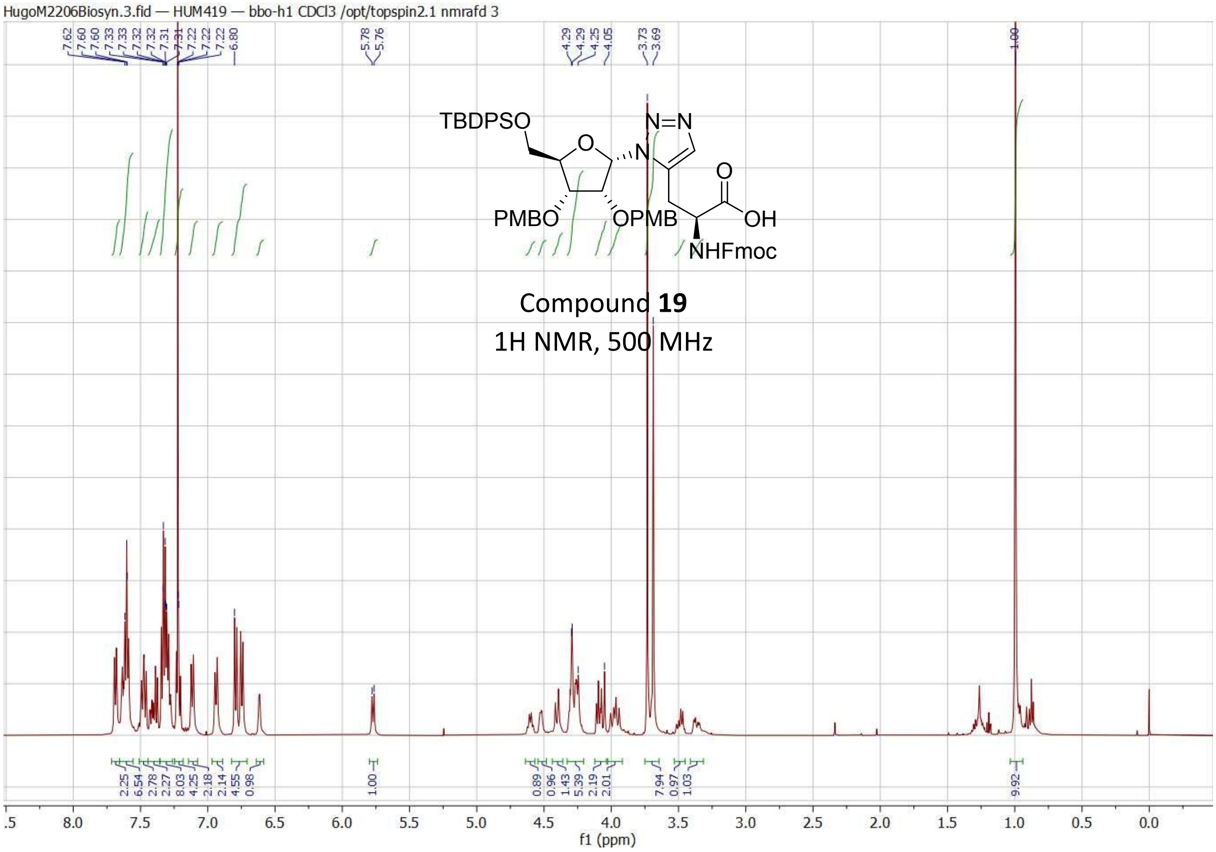

Compound **19**

1H NMR, 500 MHz

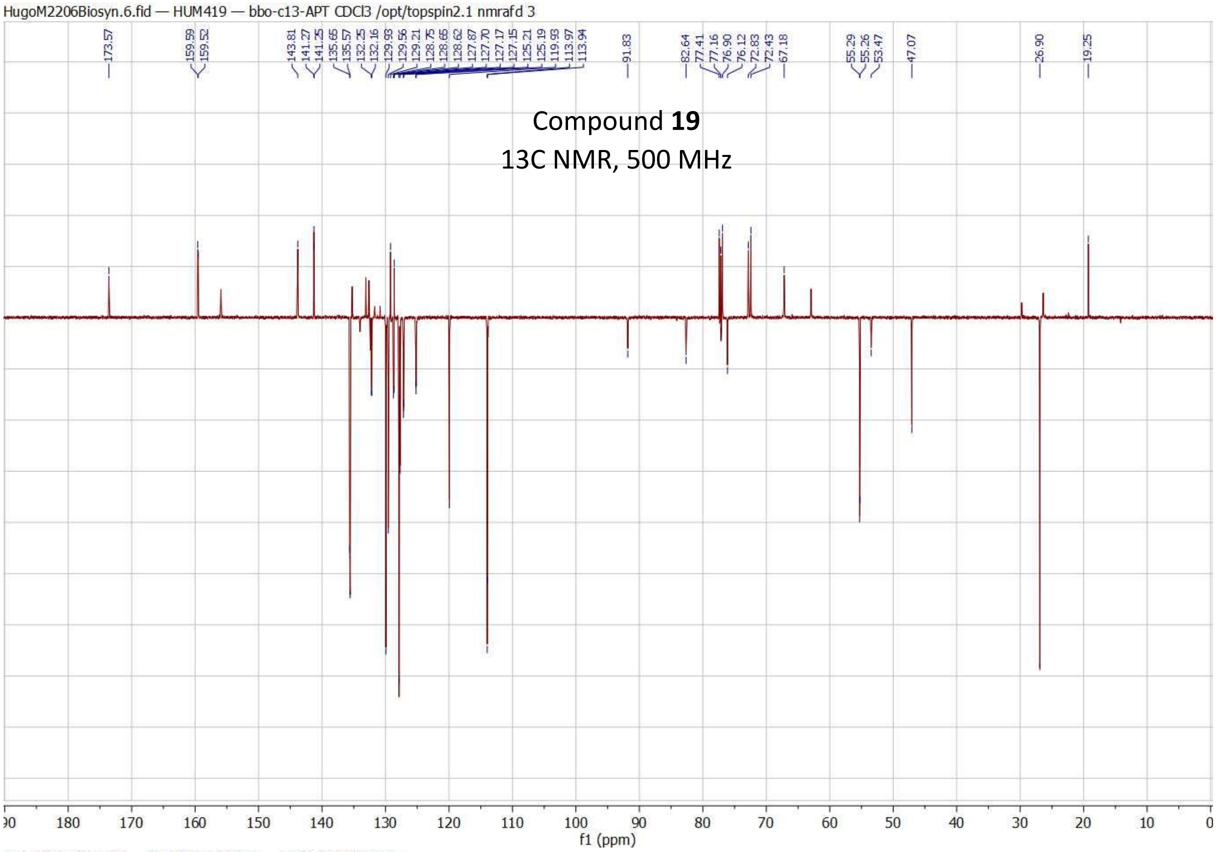
HugoM2206Biosyn.6.fid — HUM419 — bbo-c13-APT CDCl3 /opt/topspin2.1 nmrafd 3
Compound **19**
13C NMR, 500 MHz
f1 (ppm)

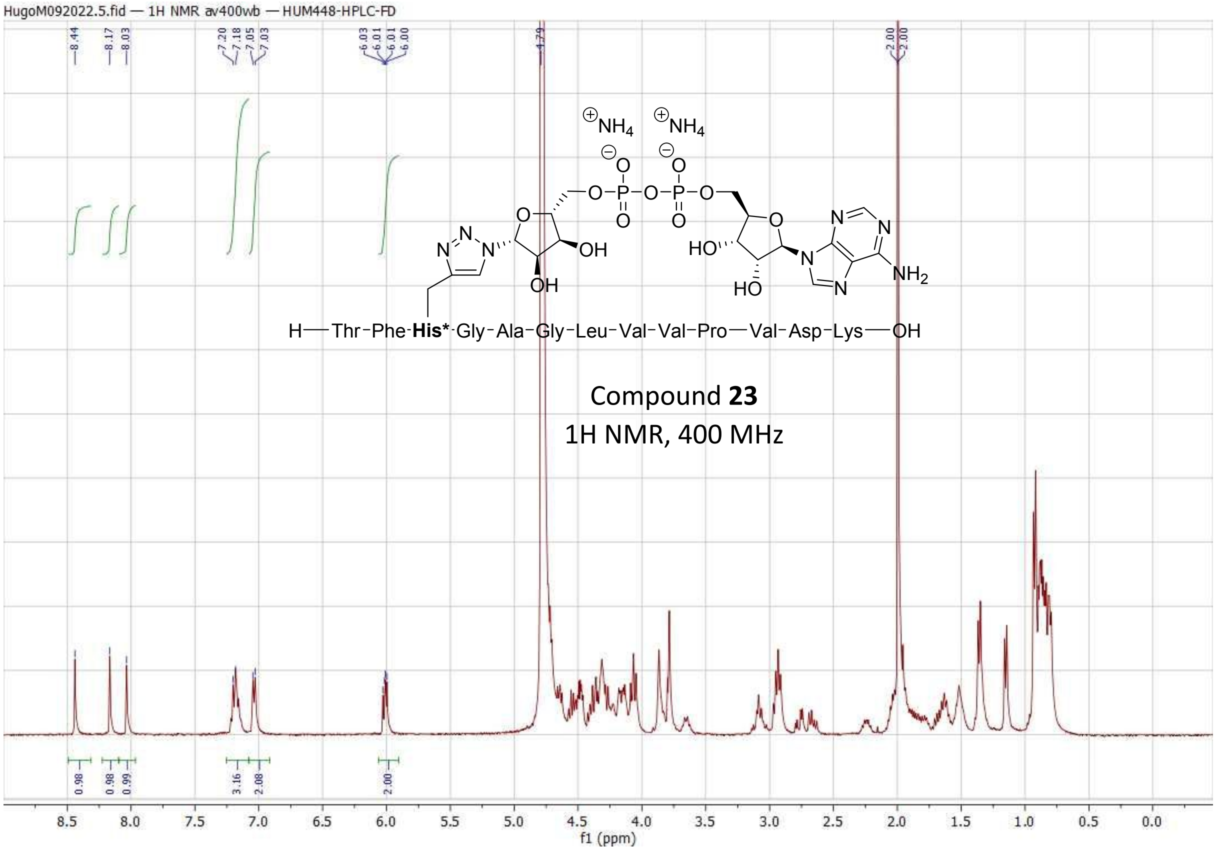
HugoM092022.5.fid — 1H NMR av400wb — HUM448-HPLC-FD
Compound **23**
1H NMR, 400 MHz
H—Thr-Phe-**His***-Gly-Ala-Gly-Leu-Val-Val-Pro—Val-Asp-Lys—OH
f1 (ppm)

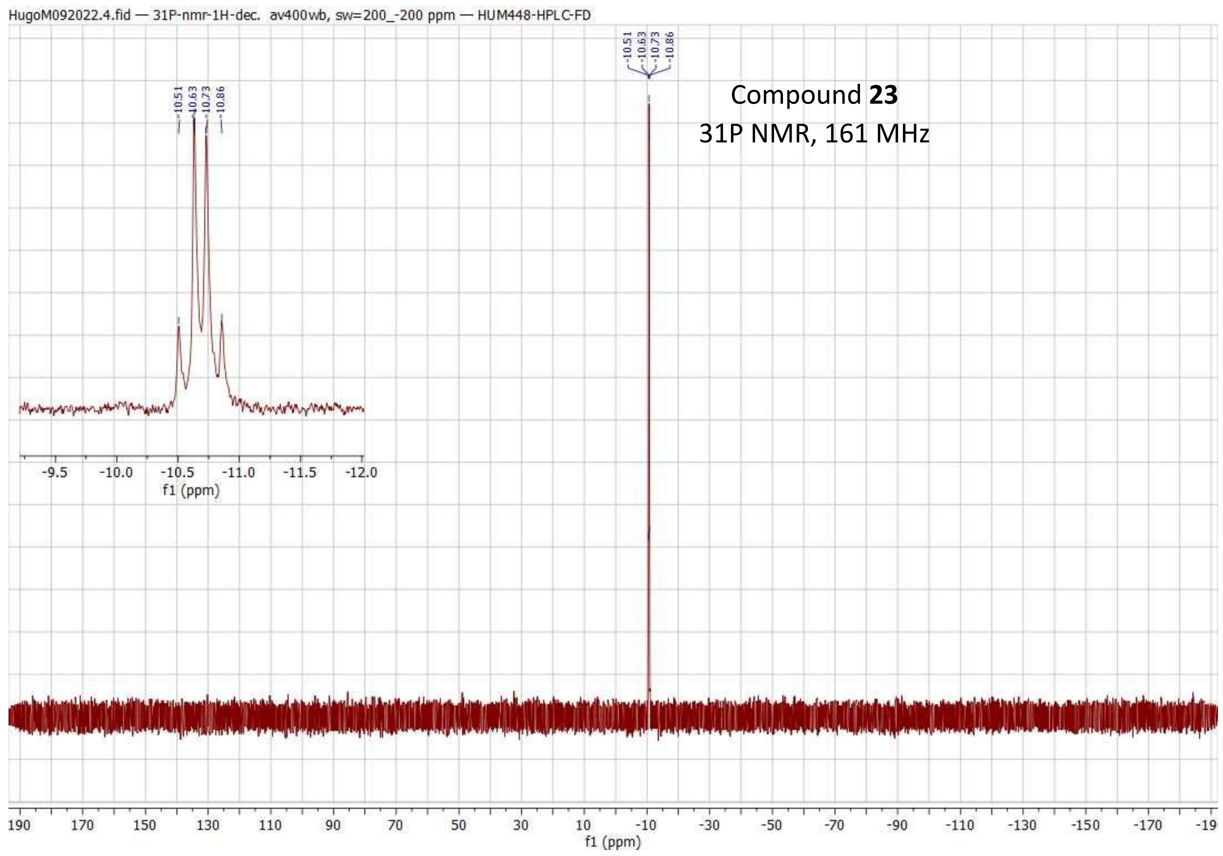
HugoM092022.4.fid — 31P-nmr-1H-dec. av400wb, sw=200_-200 ppm — HUM448-HPLC-FD
Compound **23**
31P NMR, 161 MHz
-10.51
-10.63
-10.73
-10.86
f1 (ppm)

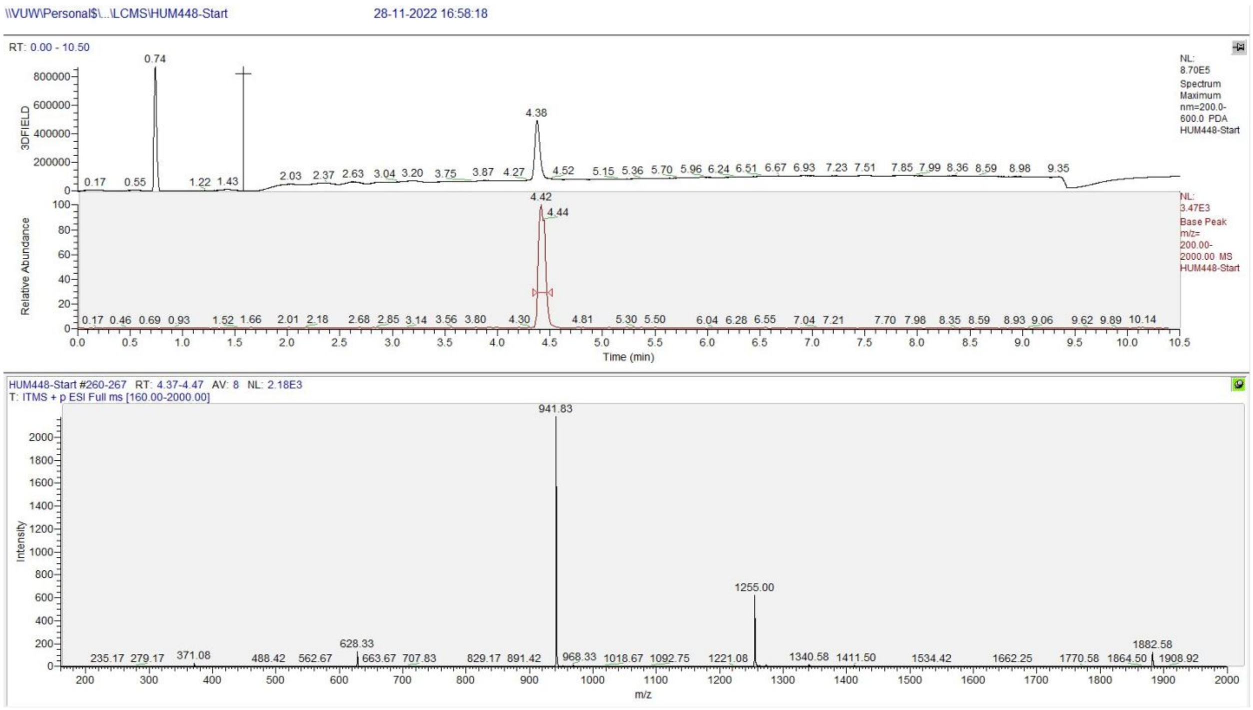
\\VUW\Personal$\...\LCMS\HUM448-Start
28-11-2022 16:58:18
RT: 0.00 - 10.50
NL: 8.70E5 Spectrum Maximum nm=200.0-600.0 PDA HUM448-Start
NL: 3.47E3 Base Peak m/z= 200.00-2000.00 MS HUM448-Start
3DFIELD
Relative Abundance
Time (min)
HUM448-Start #260-267 RT: 4.37-4.47 AV: 8 NL: 2.18E3
T: ITMS + p ESI Full ms [160.00-2000.00]
941.83
1255.00
1882.58
Intensity
m/z

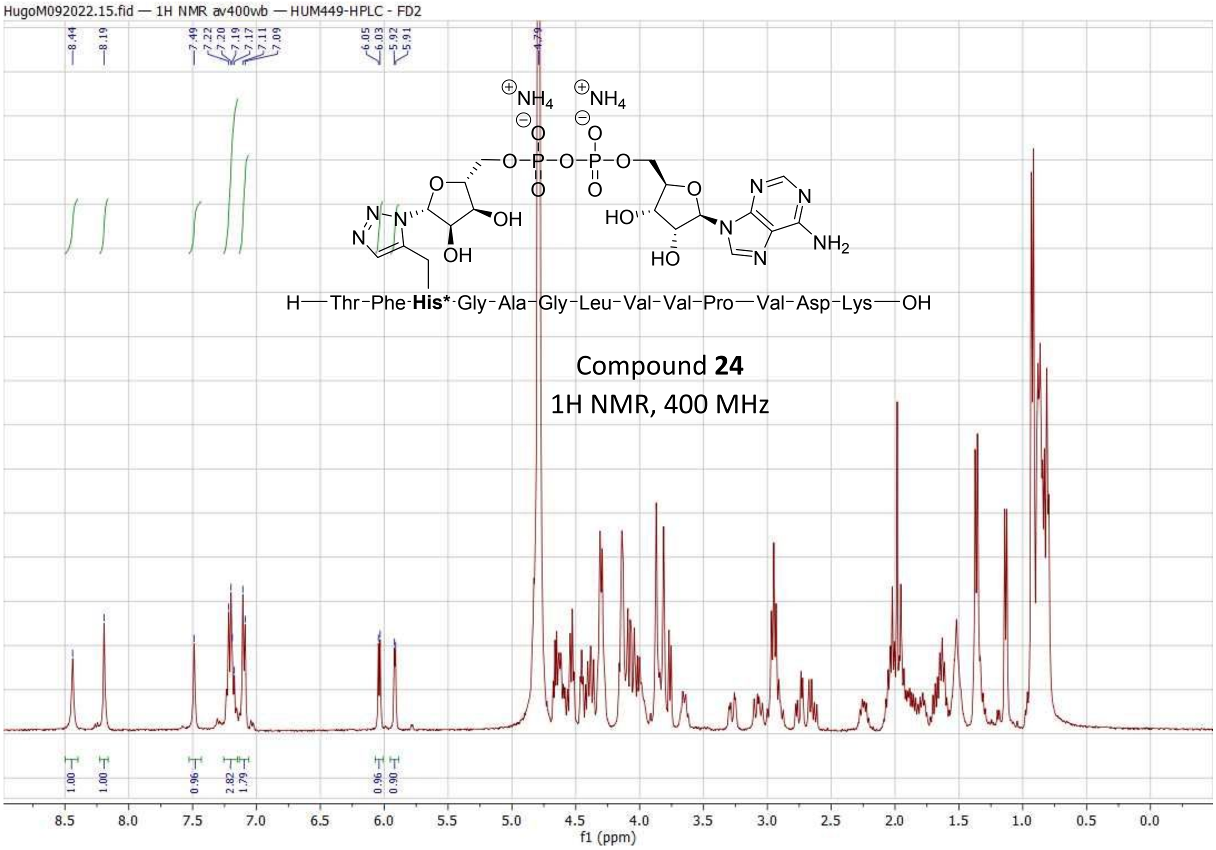
HugoM092022.15.fid — 1H NMR av400wb — HUM449-HPLC - FD2
H—Thr-Phe-His*-Gly-Ala-Gly-Leu-Val-Val-Pro—Val-Asp-Lys—OH
Compound 24
1H NMR, 400 MHz
f1 (ppm)

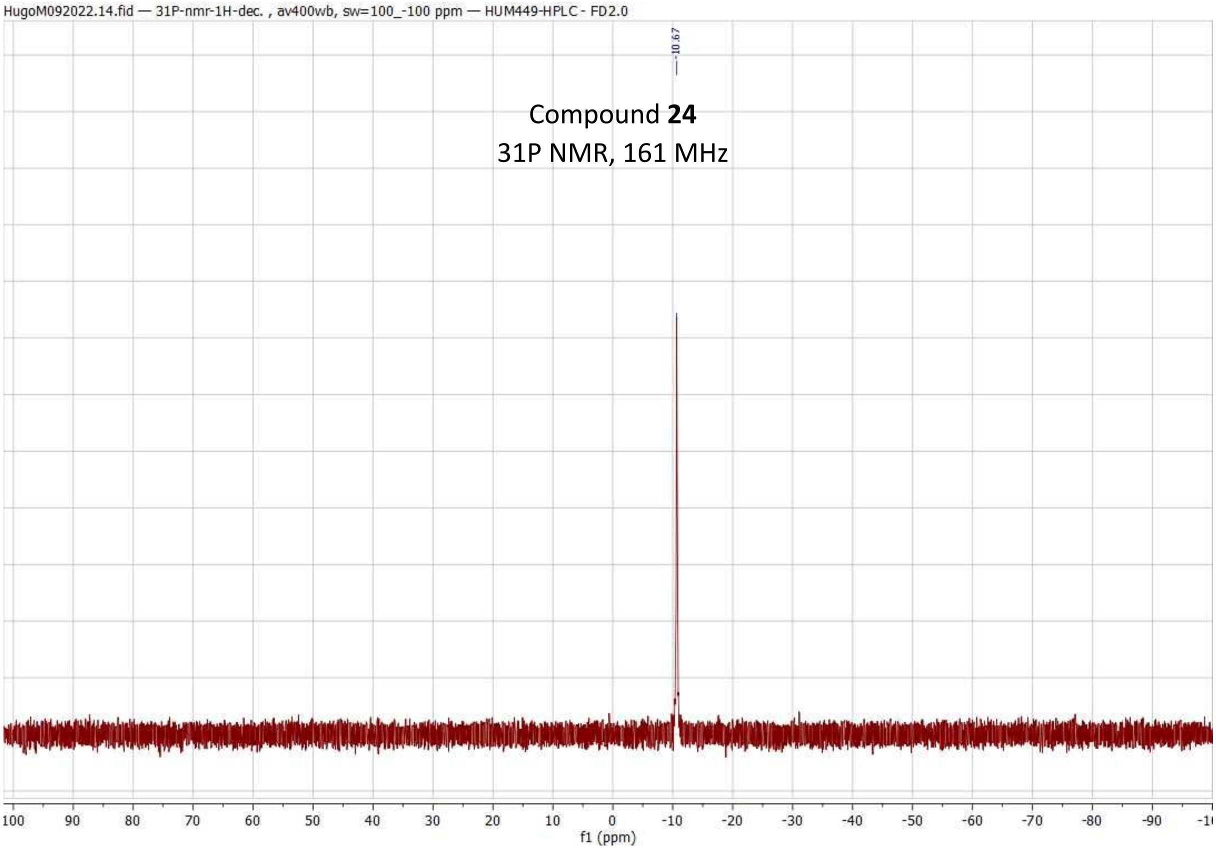
HugoM092022.14.fid — 31P-nmr-1H-dec. , av400wb, sw=100_-100 ppm — HUM449-HPLC - FD2.0
Compound 24
31P NMR, 161 MHz
f1 (ppm)

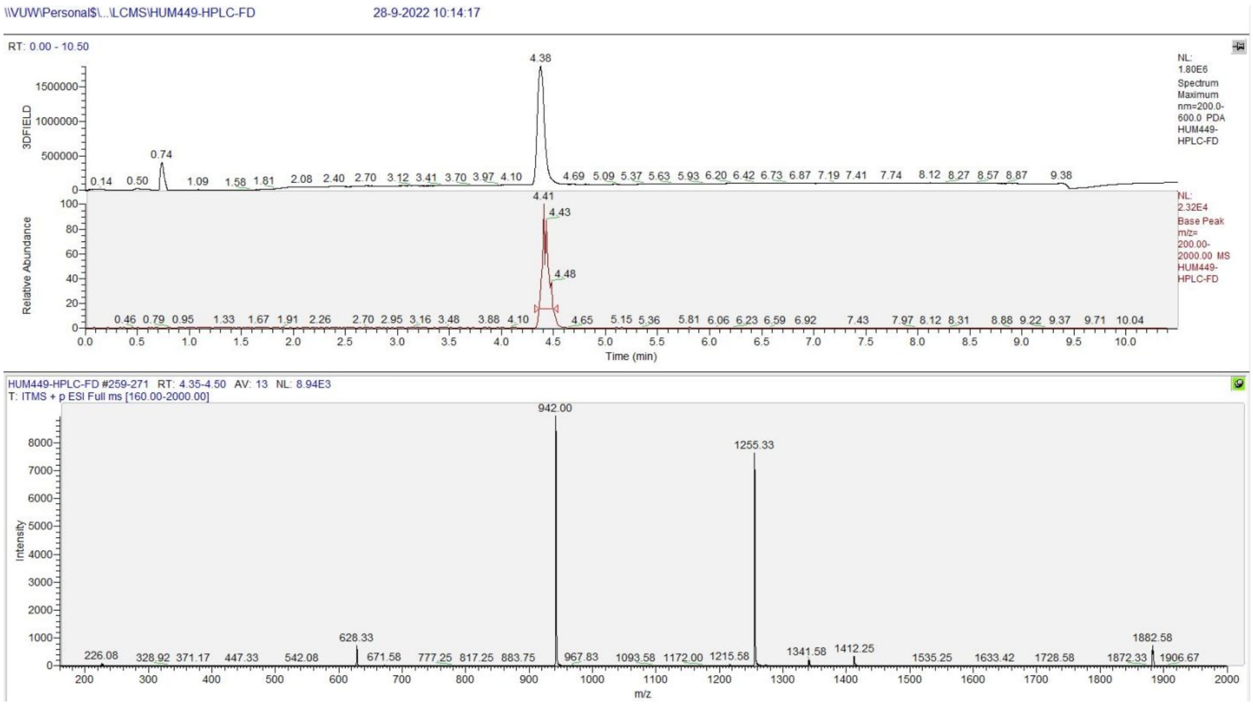
\\VUW\Personal$\...\LCMS\HUM449-HPLC-FD
28-9-2022 10:14:17
RT: 0.00 - 10.50
HUM449-HPLC-FD #259-271 RT: 4.35-4.50 AV: 13 NL: 8.94E3
T: ITMS + p ESI Full ms [160.00-2000.00]
942.00
1255.33
628.33
1882.58
Time (min)
m/z
Relative Abundance
Intensity

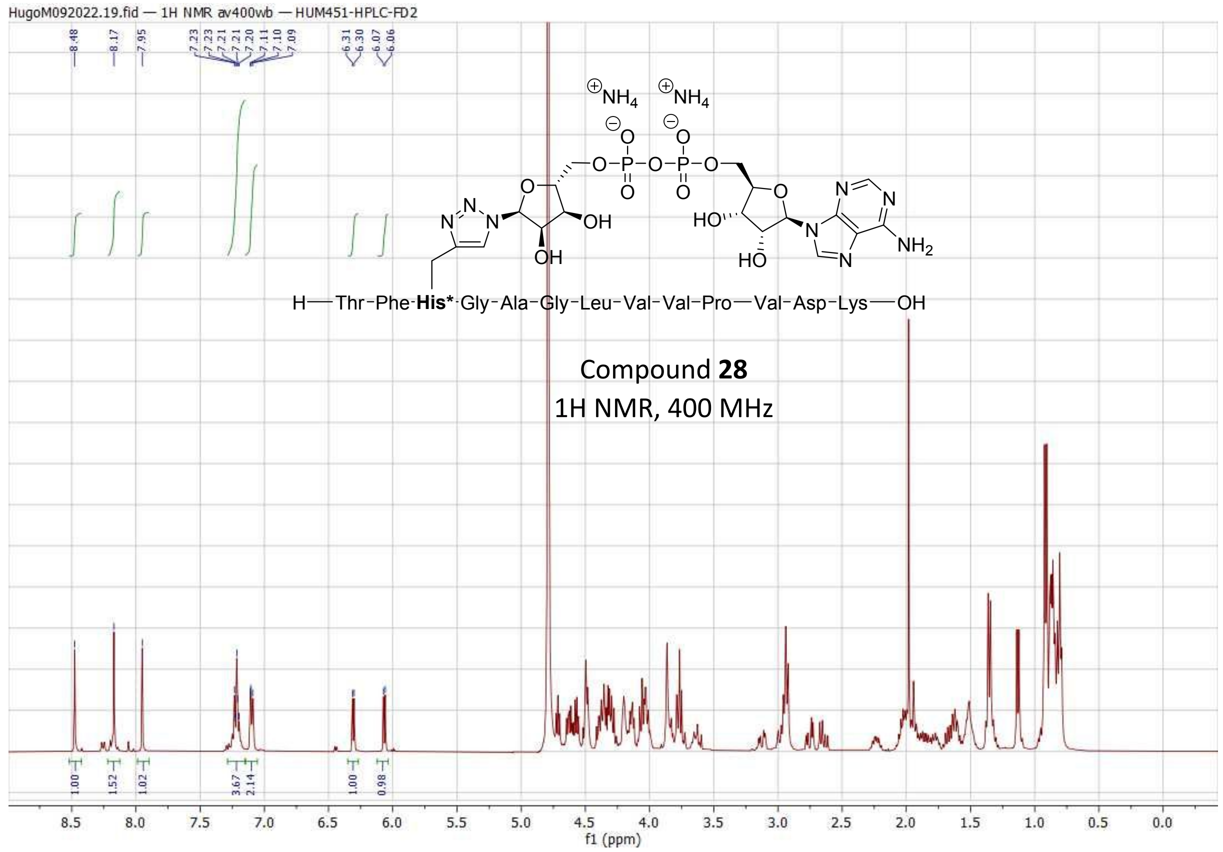
HugoM092022.19.fid — 1H NMR av400wb — HUM451-HPLC-FD2
H—Thr-Phe-His*-Gly-Ala-Gly-Leu-Val-Val-Pro—Val-Asp-Lys—OH
Compound 28
1H NMR, 400 MHz
f1 (ppm)

HugoM092022.20.fid — 31P-nmr-1H-dec. , av400wb, sw=100_-100 ppm — HUM451-HPLC-FD2

Compound **28**

31P NMR, 161 MHz

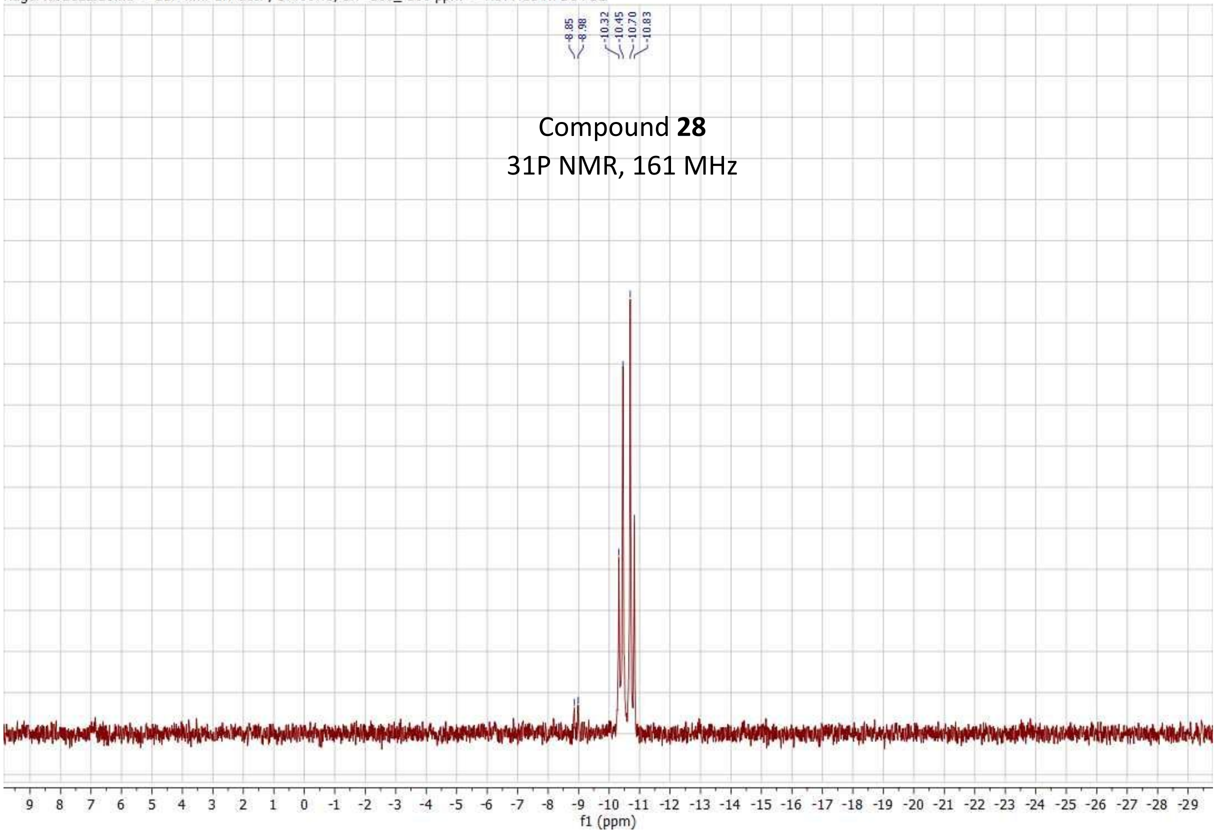

\\VUW\Personal$\...\LCMS\HUM451-Start 28-11-2022 17:11:18

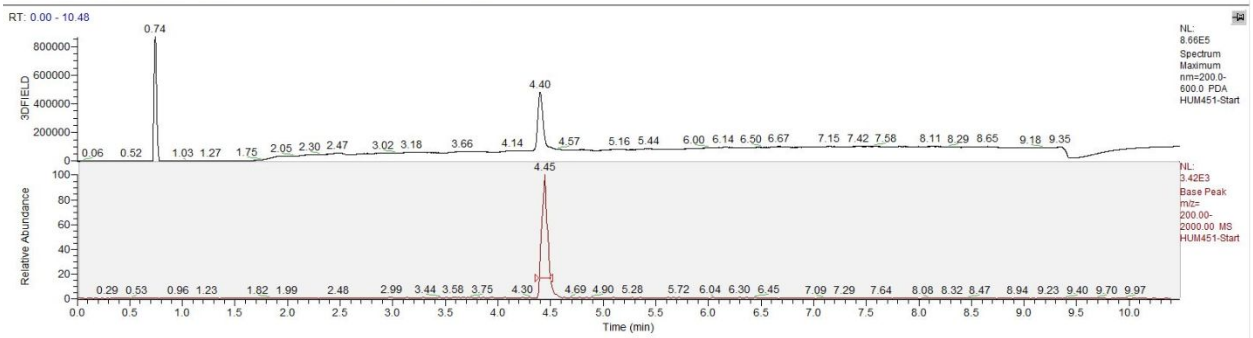

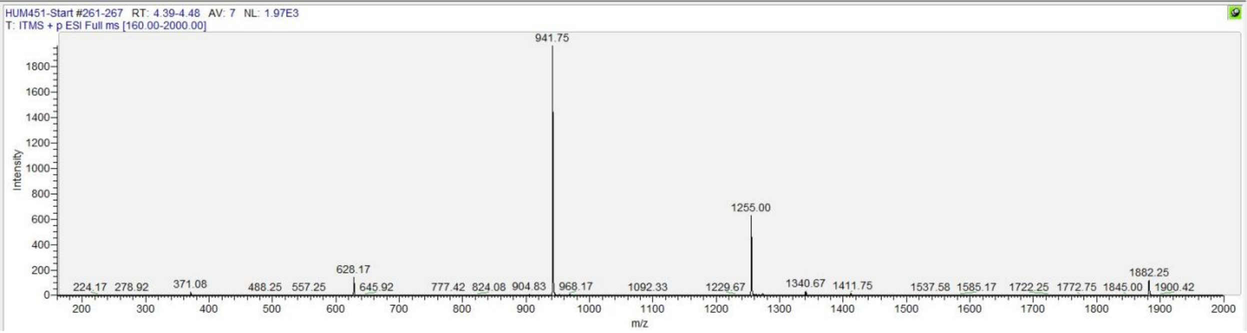

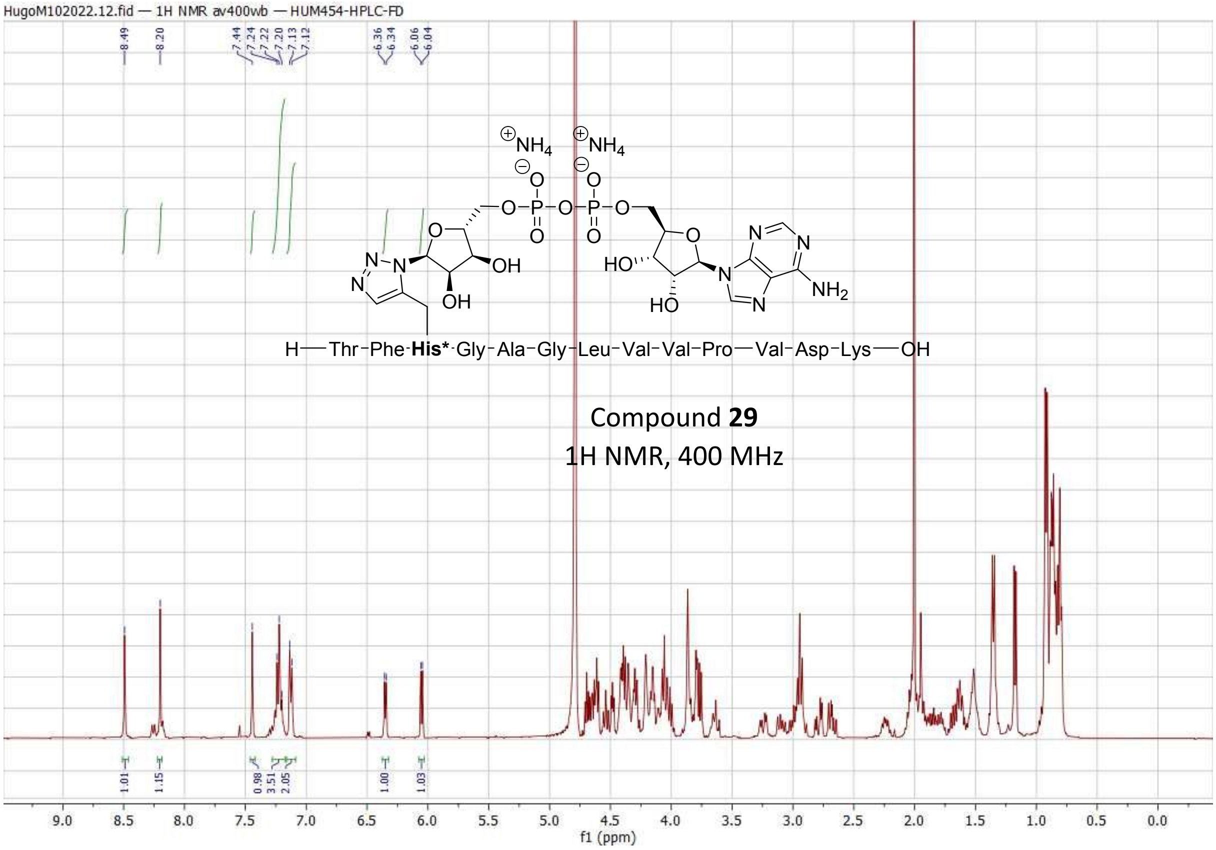
HugoM102022.12.fid — 1H NMR av400wb — HUM454-HPLC-FD
8.49
8.20
7.44
7.24
7.22
7.20
7.13
7.12
6.36
6.34
6.06
6.04
H—Thr-Phe-His*-Gly-Ala-Gly-Leu-Val-Val-Pro—Val-Asp-Lys—OH
Compound 29
1H NMR, 400 MHz
1.01
1.15
0.98
3.51
2.05
1.00
1.03
f1 (ppm)

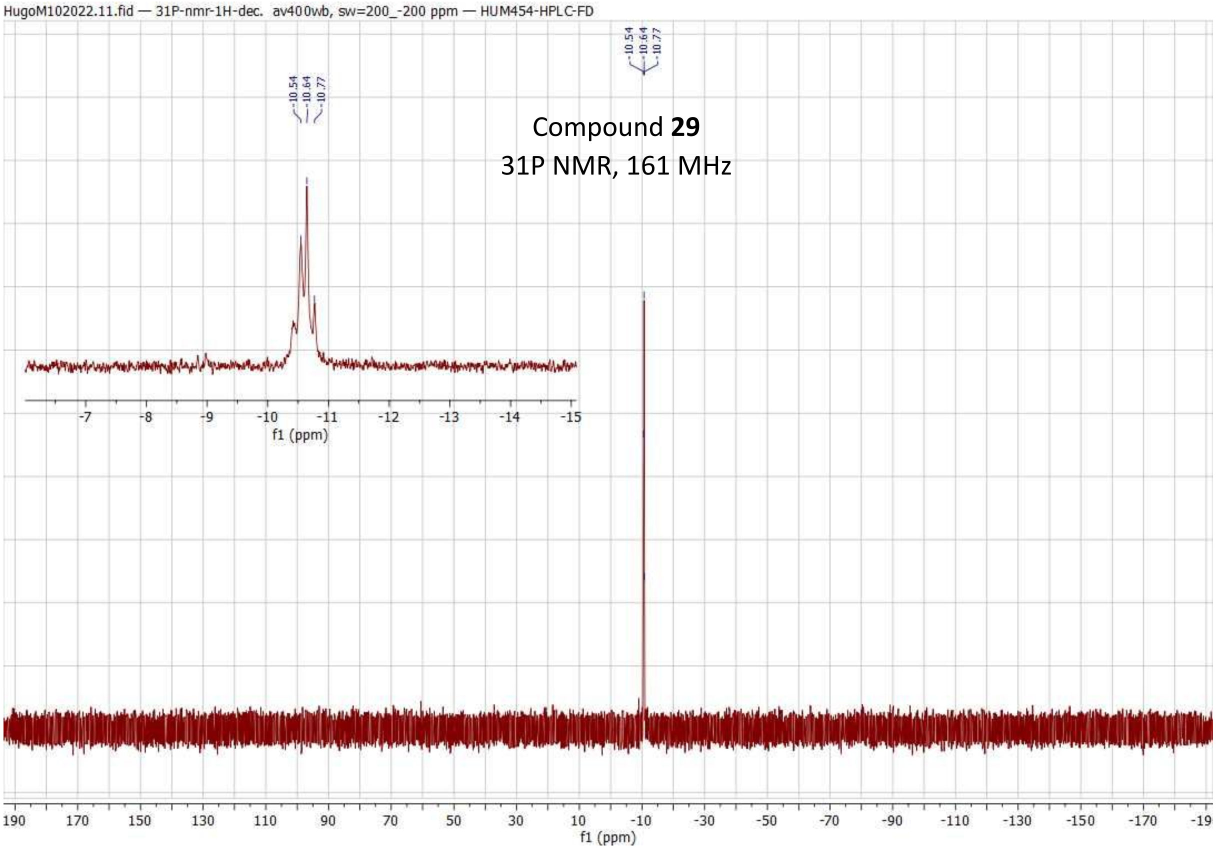
HugoM102022.11.fid — 31P-nmr-1H-dec. av400wb, sw=200_-200 ppm — HUM454-HPLC-FD
-10.54
-10.64
-10.77
Compound 29
31P NMR, 161 MHz
f1 (ppm)

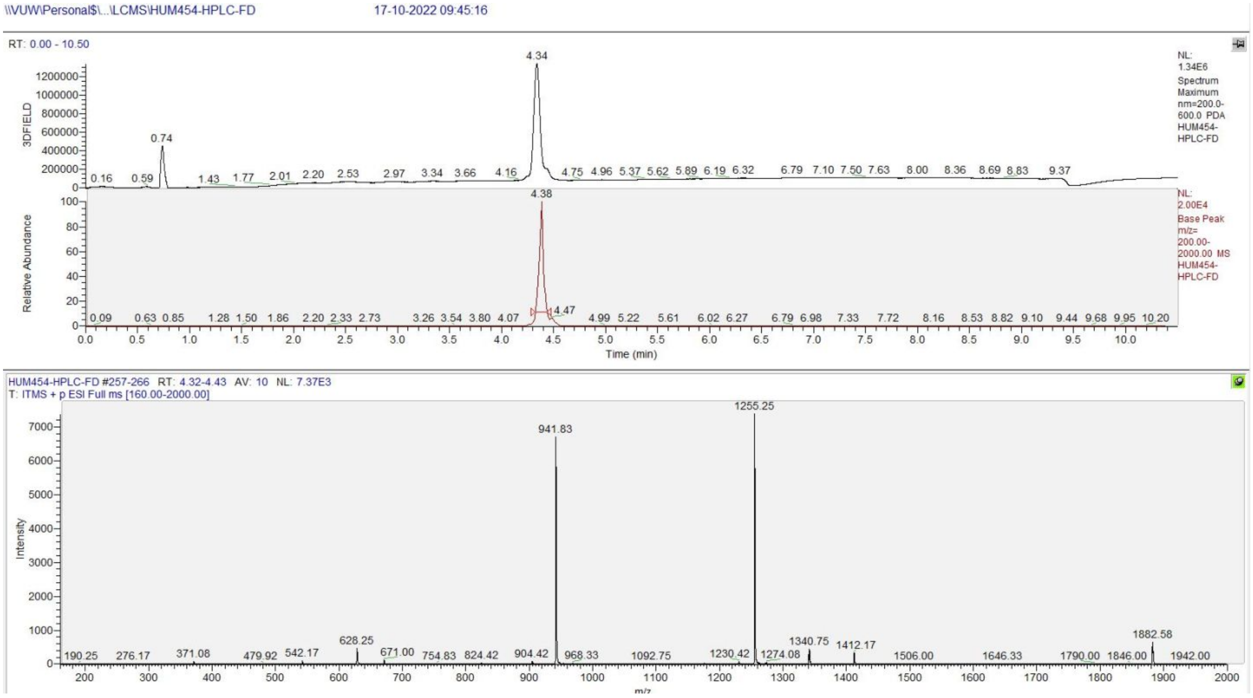
\\VUW\Personal$\...\LCMS\HUM454-HPLC-FD
17-10-2022 09:45:16
RT: 0.00 - 10.50
NL: 1.34E6 Spectrum Maximum nm=200.0-600.0 PDA HUM454-HPLC-FD
NL: 2.00E4 Base Peak m/z= 200.00-2000.00 MS HUM454-HPLC-FD
3DFIELD
Relative Abundance
Time (min)
HUM454-HPLC-FD #257-266 RT: 4.32-4.43 AV: 10 NL: 7.37E3
T: ITMS + p ESI Full ms [160.00-2000.00]
Intensity
941.83
1255.25
1882.58
m/z
